# Sticky Genomes: Using NGS Evidence to Test Hybrid Speciation Hypotheses

**DOI:** 10.1371/journal.pone.0154911

**Published:** 2016-05-17

**Authors:** Mary Morgan-Richards, Simon F. K. Hills, Patrick J. Biggs, Steven A. Trewick

**Affiliations:** 1 Ecology Group, Institute of Agriculture and Environment, Massey University, Palmerston North, New Zealand; 2 Infectious Disease Research Centre, Institute of Veterinary, Animal & Biomedical Sciences, Massey University, Palmerston North, New Zealand; Montana State University Bozeman, UNITED STATES

## Abstract

Hypotheses of hybrid origin are common. Here we use next generation sequencing to test a hybrid hypothesis for a non-model insect with a large genome. We compared a putative hybrid triploid stick insect species (*Acanthoxyla geisovii*) with its putative paternal diploid taxon (*Clitarchus hookeri*), a relationship that provides clear predictions for the relative genetic diversity within each genome. The parental taxon is expected to have comparatively low allelic diversity that is nested within the diversity of the hybrid daughter genome. The scale of genome sequencing required was conveniently achieved by extracting mRNA and sequencing cDNA to examine expressed allelic diversity. This allowed us to test hybrid-progenitor relationships among non-model organisms with large genomes and different ploidy levels. Examination of thousands of independent loci avoids potential problems produced by the silencing of parts of one or other of the parental genomes, a phenomenon sometimes associated with the process of stabilisation of a hybrid genome. Transcript assembles were assessed for evidence of paralogs and/or alternative splice variants before proceeding. Comparison of transcript assemblies was not an appropriate measure of genetic variability, but by mapping reads back to clusters derived from each species we determined levels of allelic diversity. We found greater cDNA sequence diversity among alleles in the putative hybrid species (*Acanthoxyla geisovii*) than the non-hybrid. The allelic diversity within the putative paternal species (*Clitachus hookeri*) nested within the hybrid-daughter genome, supports the current view of a hybrid-progenitor relationship for these stick insect species. Next generation sequencing technology provides opportunities for testing evolutionary hypotheses with non-model organisms, including, as here, genomes that are large due to polyploidy.

## Introduction

**Fig 1:**
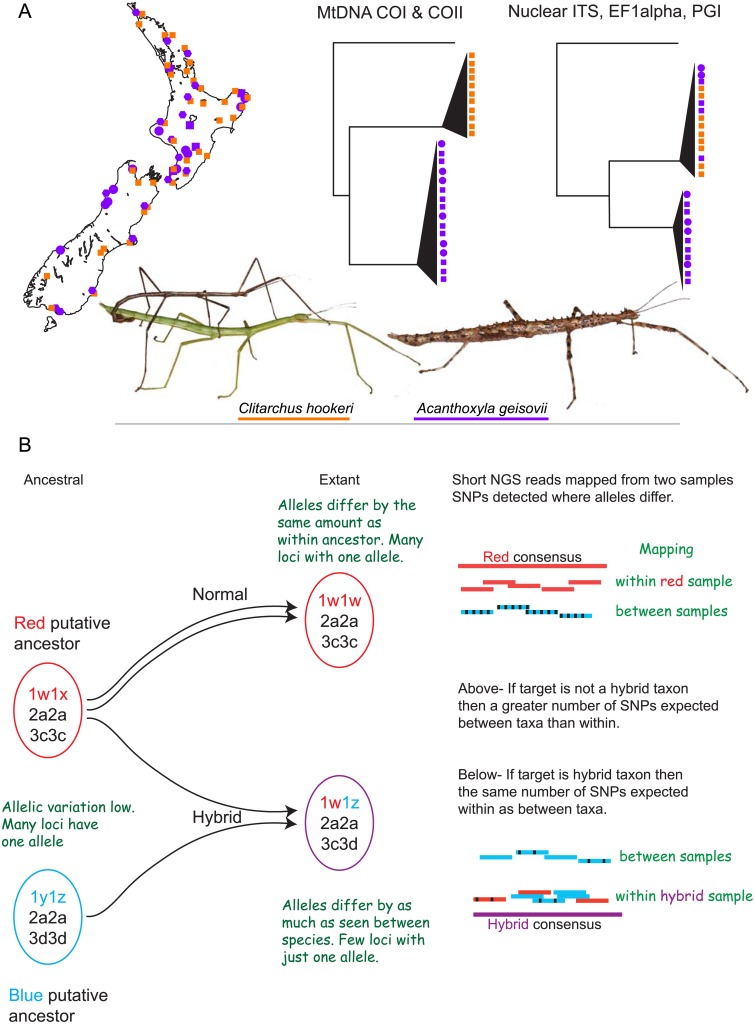
New Zealand stick insects illustrate hybrid speciation hypotheses that arise from evolutionary studies. (A) Two lineages of stick insects have been sampled across their range in New Zealand and by contrasting maternal relationships from mitochondrial DNA sequences with bi-parental multicopy nuclear markers a role for hybridisation has been inferred. Diploid *Clitarchus hookeri* (orange squares) has both sexual and asexual populations. No males of any of the *Acanthoxyla* forms are known (purple squares diploid females, purple circles triploid females). (B) Hybrid species are the product of interspecific mating resulting in genomes that are a mix of the two parental species but are reproductively isolated from both these parent taxa. The resulting allelic diversity is illustrated and compared to the diversity expected within non-hybrids and autopolyploids. When short DNA sequence reads are mapped to parents, related and non-related species, allelic similarities can be used to infer origins.

Hybridisation between species can combine divergent genomes and produce new species when reproductive isolation from parentals accompanies novel genome fusion [1]. Polyploidy and selfing commonly co-occur with hybridisation in plants leading to a high frequency and multiple origins of hybrid plant taxa [2]. In fungi, hyphal fusion generates hybrids when normally geographically isolated species are brought into contact. In animals, the origin of new species via hybridisation might be relatively rare, but in those taxa where parthenogenetic reproduction has evolved many times, as in phasmids [3], geckos [4], and frogs [5], hybrid species are well documented. Hybrid species can be recognized by the presence in a single genome of alleles that are otherwise distinct to two separate evolutionary lineages or species (Fig 1). How hybrid genomes become stabilized and how fitness costs influence hybrid survival is poorly understood [6], but our estimate of hybrid species frequency is improving [7]; [2].

Hypotheses of hybrid origin based on morphologically intermediate traits were, in the past, tested with genetic evidence that relied on universal markers [8] or lengthy development of species’ specific loci [9]. Multicopy genes present additional complexity, and as polyploidy is frequently associated with successful hybrid species [10] this is not a trivial problem. Next generation (high throughput) DNA sequencing provides an opportunity to generate information suitable for testing hybrid origin hypotheses in non-model organisms, an important objective if theory based on model organisms is to be applied to our understanding of Earth’s biodiversity. Here we set out a procedure for evaluating such data using assembled transcripts to compare ‘allelic’ diversity in a putative hybrid lineage and its putative paternal taxon. The process of stabilising a hybrid genome might involve cellular mechanisms such as gene silencing of alleles from one or other parental genome, and this might establish rapidly [11–13]. By sampling a large number of loci and comparing both within and between samples of cDNA, potential problems arising from differential silencing are minimized.

In New Zealand a genus of eight morphologically distinct species of stick insect (Phasmida) have been studied because the entire group lacks males. Each species of *Acanthoxyla* differs in how spiny it is, the presence/absence of abdominal flanges, and the sculpturing of its eggs [14]. Every individual is female and reproduces parthenogenetically producing viable offspring without males. A hybrid origin for the genus involving the ancestor of a related endemic bisexual species, *Clitarchus hookeri*, was inferred from a combination of mtDNA and nuclear markers [15]. A maternal bisexual species has not been identified and is likely to be extinct [16, 17]. In addition, many lineages of *Acanthoxyla* are mosaic triploids [18]. This pattern of polyploidy and hybrid origin has been inferred for many organisms including stick insect lineages in Europe and north Africa [19–21].

The whole *Acanthoxyla* genus has shallow mitochondrial divergence (<3%; COI-COII) and morphological “species” are not reciprocally monophyletic [15]. No partitioning by geography or diet has been suggested for the eight *Acanthoxyla* morphospecies. A hybrid origin for the genus and subsequent loss of heterozygosity might explain the current morphological diversity. Multi-copy nuclear markers (ITS, PGI, EF1a) and chromosome evidence identify *Clitarchus hookeri* as a likely parent taxon of the *Acanthoxyla* lineage (Fig 1a), although hybridisation between *Acanthoxyla* species and introgression from *Clitarchus hookeri* is possible if male *Acanthoxyla* existed in the past [8, 15, 18]. However, the absence of *C*. *hookeri* alleles in some *Acanthoxyla* individuals [8] could be the result of recombination we know occurs [18]. *Clitarchus hookeri* has all-female populations in the south of its range but crucially for hybridisation has males in sexual populations further north [22]. All *Acanthoxyla* lineages are sympatric with *Clitarchus hookeri* [15, 22].

*Acanthoxyla* sticks insects have large genomes, with approximately three times as much DNA per cell as humans (~9 pg; [18, 23]), and have not yet had their genome sequenced. To generate a manageable amount of data we reduced the genome sample by extracting mRNA. We used cDNA sequences generated from this to examine the expressed allelic diversity within one *Acanthoxyla* lineage and compared this to sequences from *Clitarchus hookeri*. The hybrid origin hypothesis predicts that *Acanthoxyla geisovii* will share alleles with the putative parental species *Clitarchus hookeri*, but will also contain alleles unique to the *Acanthoxyla geisovii* genome (inherited from the maternal parent species). The *Acanthoxyla geisovii* lineage investigated here is triploid and *Clitarchus hookeri* is diploid [15, 18]. If *Acanthoxyla geisovii* is not of hybrid origin then at each locus, all alleles within *Acanthoxyla geisovii* will be more similar to each other than they are to *Clitarchus hookeri* alleles. Even if the paternal species has not been correctly identified, the extent of allelic diversity in a parthenogenetic lineage ought to provide evidence of genome fusion. We used approaches to ensure our datasets were robust for assumptions of homology including manual curation of a subset of data to establish that sequence clusters did not contain mixed products of multigene families. We identified and separately analysed the higher GC content components of our data to ensure non-protein coding transcripts were not misleading us, and we searched public databases to confirm our samples were contaminant free. Preliminary functional analyses compared translated sequences with arthropod datasets to identify orthologous groups.

We mapped sequence reads from each stick insect against transcript assemblies of both to reveal asymmetry in allele diversity (Fig 1b). We compared the proportion of putative loci (transcript assemblies) where identical reads were found after mapping total sequence datasets back to putative loci, for each genome. We calculated sequence divergence between assembled transcripts and raw reads to determine relative genetic diversity within each genome. This provided data that supported the existing hybrid origin hypotheses involving these stick insect species.

## Materials and Methods

### Source material

Total RNA was simultaneously but independently extracted from femur muscle samples from two adult female parthenogenetic stick insects using the QIAGEN RNeasy Mini kit. *Acanthoxyla* was represented by a green spiny triploid form (*A*. *geisovii*) collected from the host plant *Podocarpus totara* (totara) in Manawatu, New Zealand (40°24'55.86"S, 175°39'50.05"E; Ax.PN-762). *Clitarchus hookeri* was represented by a brown female collected from the host plant *Rubus fruticosus* (bramble) in Kapiti Coast, New Zealand (40°51'35.37"S, 175° 3'4.62"E; Ch.W-765). Insects were collected from private property with the owner’s permission.

RNA was stored at -80°C until required, and analysed using a BioAnalyser, total RNA samples for mRNA: Quant-iT RNA: 90–100 ug/mL; Quant-iT dsDNA 18–31 ug/mL; Quant-iT ssDNA: 24–87 ug/mL; Quant-iT Protein 714–737 ug/mL. High protein content does not interfere with mRNA sequencing library preparation. The polyA mRNA was extracted using oligo dT magnetic-beads (protein and DNA remained in the supernatant). The BioAnalyzer measurements revealed a low level of RNA degradation in the *Acanthoxyla* sample only.

### Sequence generation

The RNA samples were made into libraries using an Illumina mRNA-Seq Sample Preparation Kit (part no. RS-100-0801) and were indexed using the index sequence 5´CAAGCAGAAGACGGCATACGAGATAAGCTAGTGACTGGAGTTC for *Acanthoxyla geisovii*, and 5´CAAGCAGAAGACGGCATACGAGATGTAGCCGTGACTGGAGTTC for *Clitarchus hookeri*. The libraries were then pooled by equal molarity and loaded at 42.5 pM and run in a single lane on an Illumina GAII_X_ to generate single 100 base reads. The run was processed with RTA v.1.6 and Casava v.1.6 to demultiplex the data and generate the short read sequence files for each species. The short reads were analysed with SolexaQA [24] to assess the quality of the run, and to inform the trimming of reads to the desired quality for subsequent assemblies.

### De novo assembly and clustering

**Fig 2:**
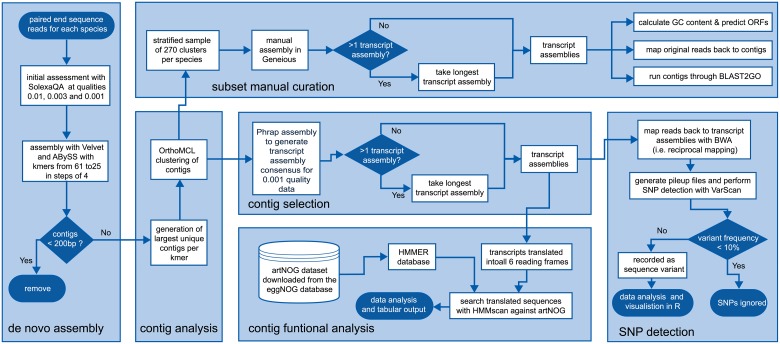
Bioinformatic pipeline for analysis of cDNA from non-model organisms with large polyploid genomes to test hybrid origin hypotheses.

*De novo* assemblers Velvet v.1.2.10; [25] and ABySS v.1.3.2; [26] were used to assemble the short reads from each species. A combination of k-mer assembly parameters (from 25 to 61 in steps of 4), pre-trimming of the data to remove any Illumina TruSeq adapters (resulting from small inserts for example) and quality-trimmed data (DynamicTrim at quality thresholds of 0.01, 0.003 and 0.001) were tried for each species, resulting in 120 combinations of assembly parameters (S1 Table). In addition, Velvet assemblies were performed with a minimum contig output length of 200bp. As this k-mer sweep approach generated many sequences that were almost identical (varying only at the ends due to the k-mers), a custom Perl script was used to generate a unique set of contigs from each k-mer sweep (S2 Table). The trimmed and unique contigs were then used as input for a clustering procedure using OrthoMCL v.2.0 [27, 28] with default parameters to generate clusters of sequence contigs for further analysis (Fig 2). Clustering of sequences was necessary so that each transcript could be treated as an independent locus in downstream analyses; sequences within each cluster are not independent units.

### Manual curation of a subsample of clusters

A stratified random sample of 270 clusters (~10%) was taken from each single species set for manual curation and assessment (a total of 540 clusters). The sample was stratified to favor clusters containing a greater number of sequences as these clusters contained a greater proportion of the useful data and also avoided oversampling from the large number of clusters containing only two sequences. Clusters were assembled using the *de novo* assembly tool in Geneious v.6.1.6 (Biomatters; http://www.geneious.com). The quality of the resulting transcript assemblies was assessed on the number of transcript assemblies returned from the assembly of each cluster, and the quantity and distribution of sequence variability across the transcript assemblies. The number of transcript assemblies provides a measure of the effectiveness on the clustering algorithm; where more than one super-contig is generated for a given cluster this indicates that at least two different transcripts have been incorrectly assigned to the same cluster. Where more than one super-contig was generated for a cluster, the longest was taken for further analysis. Assessment of the degree and distribution of nucleotide variability across the transcript assembly of a given cluster provides an indication of the erroneous clustering of closely related paralogs or splice variants. As such artefacts can be especially misleading when analyzing data from polyploids, dubious sequences were removed to produce transcript assemblies of contig sequences representing single loci. In addition, observed nucleotide disagreements involving the first or last five bases of contributing contig sequences were resolved by deleting these ends. These errors appear to have resulted from miss-calls in the initial assembly of sequence reads into contigs in which the depth of coverage is reduced at the ends of contigs. Consensus sequences of each of the transcript assemblies were generated in Geneious. Open reading frames were predicted for each consensus sequence, and GC content was calculated. In order to confirm the nucleotide variability observed in the transcript assemblies, sequence reads were mapped back to these consensus sequences. The GC content was also calculated for primate protein coding genes with apparent homologs in our stick insect dataset. This was compared to the GC content of the stick insect sequence in order to determine a threshold for GC content of protein coding DNA (S1 Fig). The identity of putative protein coding ORFs was examined by a BLAST homology search using BLAST2GO [29]. The allelic diversity of a subset of genes identified in both species was further tested on a small scale by remapping reads to consensus sequences and calling SNPs (at 10% minimum frequency) in Geneious.

### Contig clustering

For each species, the clustering process generated sets of sequences that overlapped to varying degrees, and it was generally the case that the longest contig in a cluster did not cover the full consensus sequence of that cluster. To generate such a consensus sequence Phrap v.0.990329 [30] was used with default parameters on each cluster from the most stringent dataset combination (data assembled with a quality cut-off of 0.001 on the short reads that had been processed to remove adapters). In the small proportion of cases where this introduced more than one sequence only the longest was analysed further. The identity of the consensus sequences was initially assessed by a BLAST homology search using default parameters in BLAST2GO [29]. Few sequences deposited in Genbank shared similarity with our assembled stick insect transcripts (S2 Fig) so we took a functional orthology approach using the eggNOG (evolutionary genealogy of groups: Non-supervised Orthologous Groups) classification system [31]. The Arthopoda (artNOG) HMM (Hidden Markov Model) files, members and annotations datasets (http://eggnogdb.embl.de/#/app/home) were downloaded to make a local HMMER (http://hmmer.org) database following the recommended procedure on the website. As reading frame was not known, transcript assemblies were translated into all six reading frames using BioPerl to generate amino acid sequences (including stops) with their transcript names appended with the frame. The resulting sequences were then searched with ‘hmmscan’ against the artNOG HMMER reference database to generate tabular output [31]. The highest bitscore was used to select one result per assembled transcript and data summaries were generated using a MySQL database.

### SNP analysis

In order to assess allelic diversity at each locus we used a reciprocal mapping approach with short reads from each species being mapped back to transcript assemblies (loci) from itself, and the other species. It should be noted that the contig assembly process resulted in a consensus sequence from the transcript assemblies, and so some real allelic diversity would have been lost in that process. Short reads were mapped back to the longest contig from each transcript assembly cluster using the short read mapper Bowtie2 (Langmead and Salzberg 2012). We mapped short reads to all transcript assemblies simultaneously, preventing a read mapping to more than one transcript (c.f. sequential mapping). The resulting SAM files were then parsed so that only reads mapped over a certain length (12 nucleotides) were included in the subsequent SNP analysis, and a new SAM file was generated for each transcript assembly. Repeating this using 25 and 50 nucleotide length mappings had no significant effect on our findings (data not shown) so we present data for a single mapping here. The mappings from these individual SAM files were analysed with the variant detector VarScan v.2.3.2; [32], with a conservative threshold of 10% for variant frequency being taken as evidence for different alleles being present at a nucleotide position. The number of SNPs in a given transcript assembly was normalized by the transcript assembly length to get a SNP rate per nucleotide. The results of these analyses were visualised using R software [33].

## Results

**Fig 3:**
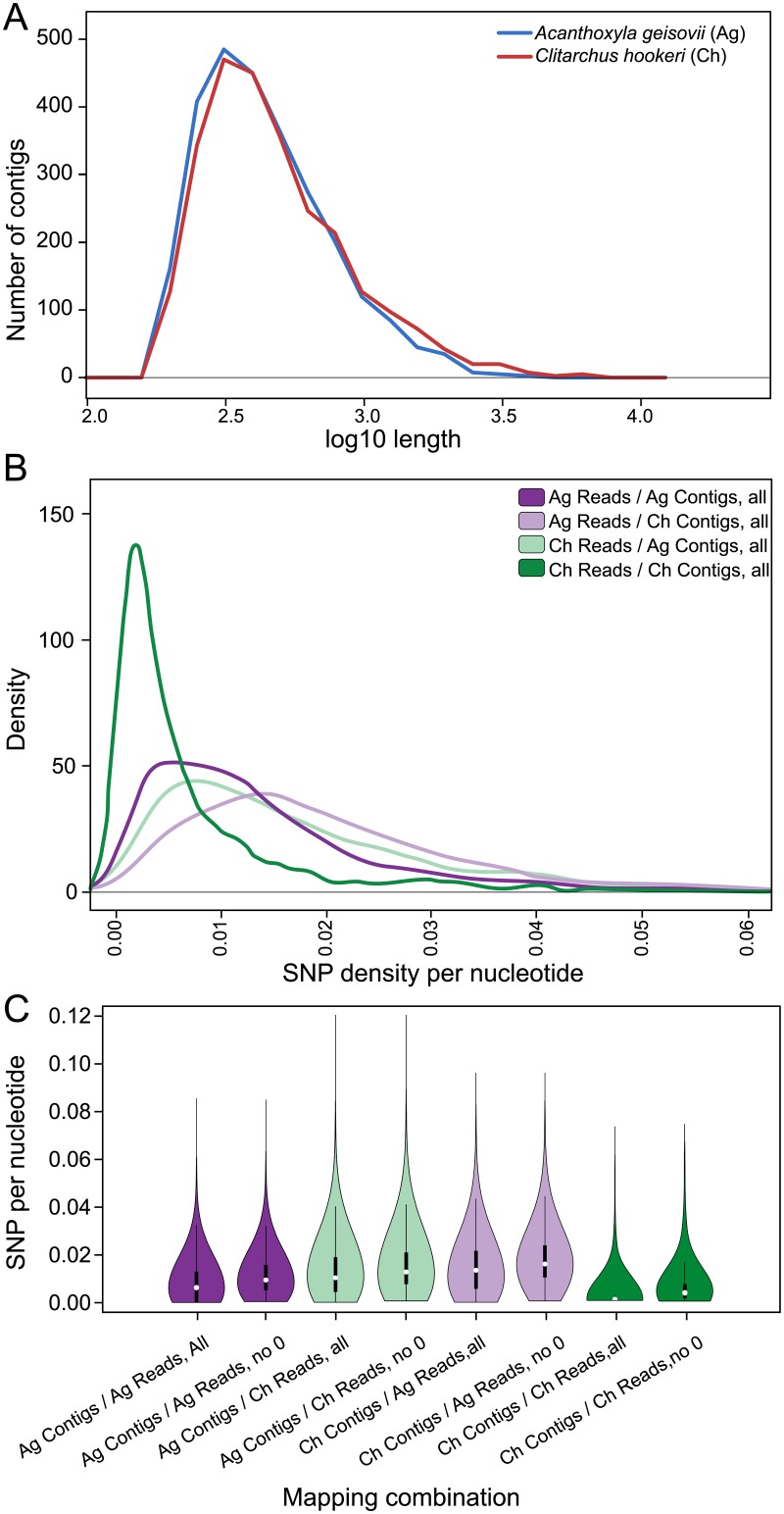
Next Generation sequencing results from mRNA of two New Zealand stick insects (A). Length distributions of transcript assemblies produced from the cDNA sequence of two stick insects were similar. A log length frequency distribution plot used values rounded to 1 decimal place for the longest consensus sequence generated from each cluster. (B) Sequence divergence (measured by SNP density per nucleotide) observed when reads were mapped to ~2,600 loci (transcript assemblies). Loci without variation (SNP-free) were removed. The putative parental *Clitarchus hookeri* genome contains many loci with low allelic diversity. SNPs detected in less than 10% of the short reads were ignored but reads were included whether or not they passed the strand bias filter within VarScan. Only the longest assembled transcripts generated per cluster were included. (C) SNPs detectable on all transcript assemblies by BWA mapping to ~2,600 *Acanthoxyla* and *Clitarchus* transcript assemblies using VarScan with minimum variant frequency of 10% irrespective of strand filter results. The first violin of each color comprises all data, and the second excludes transcript assemblies with no sequence variation (SNP-free). Purple–*Acanthoxyla* reads mapped onto *Acanthoxyla* transcript assemblies; Pale green–*Clitarchus* reads mapped onto *Acanthoxyla* transcript assemblies; Pale purple–*Acanthoxyla* reads mapped onto *Clitarchus* transcript assemblies; Green–*Clitarchus* reads mapped onto *Clitarchus* transcript assemblies.

Data quantity and quality were similar for each stick insect species following Illumina high throughput DNA sequencing (Table 1). After adaptor and quality trimming, clustering of cDNA sequences resulted in more than 2,500 transcript assemblies per species (Table 1; Fig 2; S1 and S2 Tables). These transcript assemblies had similar length distributions (Fig 3a) meeting our assumptions of equal coverage in the two taxa. Data are deposited with the Dryad Digital Repository and publically available at http://datadryad.org (doi:10.5061/dryad.h5g60), or can be downloaded from http://evolves.massey.ac.nz/DNA_Toolkit.htm.

**Table 1:** Summary of the cDNA sequences obtained from two New Zealand stick insects using the Illumina pipeline with the length distribution of transcript assemblies following trimming (at a quality of 0.001) and subsequent clustering.

	*Clitarchus hookeri*	*Acanthoxyla geisovii*
Yield (kb)	1,124,720	721,022
Raw clusters per tile	104,803 +/- 9655	65,469 +/- 3946
PF clusters per tile (bp)	60,085 +/- 3433	60,085 +/- 3433
Minimum contig length (bp)	200	167
Maximum contig length (bp)	12,855	4,504
Average contig length (bp)	608.334	532.730
Total assembled contig length (bp)	1,579,843	1,410,670
Number of Contigs	2,597	2,648

### Manually curated subsample

A subsample comprising 10% of the >2,500 clusters from each species was manually curated to identify anomalies within our pipeline. We determined that the clustering algorithm was at least 97% accurate; only 7 *Acanthoxyla geisovii* and 4 *Clitarchus hookeri* clusters generated more than one transcript assembly. Alternate splice variants were included in many transcript assemblies, but these comprise <50 bp of the alternative exon. No instances of paralogous transcript assemblies within the mismatch parameters were identified. Mapping short sequence reads back to transcript assembly consensus sequences revealed more nucleotide variability than was seen in the short read assembled transcripts. Thus, comparison of the transcript assemblies was an inappropriate measure of the genetic variability between the two stick insect species. Open reading frame (ORF) prediction and GC content calculation indicated a positive relationship between ORF length and GC content (S1 Fig). By considering sequences with a GC content of 48% or more, it was possible to exclude almost all sequences that were not dominated by predicted ORF, and thus consider a dataset consisting predominantly of protein coding DNA (S3 Fig). Manually curating a subset of the data allowed us to identify genes from conserved families such as alpha-actinin (Table 2). Expression of this actin binding protein in muscle tissue revealed greater cDNA sequence variation in *Acanthoxyla geisovii* than in *Clitarchus hookeri*. Diploid *Clitarchus hookeri* had three nucleotide polymorphisms with equal frequency of reads, while *Acanthoxyla geisovii* had 26 substitutions where frequency of reads was close to 33%, as expected of a triploid (Table 2).

**Table 2:** Alpha-actinin cDNA sequence diversity illustrates that allelic diversity of the stick insect *Acanthoxyla geisovii* is greater than within *Clitarchus hookeri* as expected of a hybrid. Within *Acanthoxyla geisovii* the SNP variant (allele) most similar to *Clitarchus hookeri* has approximately one third the expression level (inferred from read coverage) because this genome is triploid.

	*Clitarchus hookeri*			*Acanthoxyla geisovii*		
SequencePosition	SNP (primary/variant)	Read coverage	Variant frequency (%)	SNP (primary/variant)	Read coverage	Variant frequency (%)
375	A	1031	-	G / A	824	**40.8**
462	A	1281	-	G / A	704	**38.9**
492	C	1159	-	T / C	631	**35.0**
561	T	1109	-	C / T	543	**28.7**
633	G	1223	-	T / G	548	**31.0**
672	T	1081	-	C / T	569	**29.3**
828	T	787	-	C / T	600	**32.8**
928	T	1290	-	C / T	773	**30.8**
1014	G / T	802	**47.1**	G / T	719	**35.9**
1032	G / A	729	**48.1**	A	637	-
1044	G	650	-	A / G	594	**33.2**
1158	G	950	-	T / G	671	**25.5**
1164	T	940	-	G / T	707	**23.1**
1251	G	1272	-	A / G	998	**34.6**
1285	T / G	1398	**13.1**	T	1158	-
1395	C	1437	-	A / C	878	**37.7**
1404	A	1335	-	T / A	845	**35.3**
1497	T	957	-	C / T	598	**29.1**
1524	G	1260	-	C / G	787	**31.3**
1545	C	980	-	T / C	712	**30.3**
1656	T	1067	-	C / T	707	**33.9**
1662	G	1088	-	A / G	666	**36.3**
1749	G / A	1025	**50.0**	G / A	952	**32.4**
1809	C	1353	-	T / C	1216	**27.1**
1833	A	1426	-	G / A	1362	**24.1**
1875	A	1260	-	G / A	1092	**26.4**
2019	T	1032	-	C / T	644	**45.8**
2025	C	1030	-	G / C	657	**45.1**
2127	T	874	-	C / T	649	**32.2**
2136	T	929	-	C / T	706	**33.4**
2181	C	914	-	A / C	793	**34.8**
2208	C	856	-	G / C	704	**35.8**
2256	G	1087	-	A / G	755	**29.0**
2433	A	1016	-	G / A	541	**37.9**
2484	T	1034	-	C / T	506	**36.0**
2487	T	1084	-	A / T	522	**35.8**

### Full dataset

Only 13.3% of the assembled transcripts matched sequences in the National Center for Biotechnology Information (NCBI) non-redundant (nr) protein database, but 2/3 of these had their closest match to an arthropod sequence (S2 Fig, S3 and S4 Tables). A limited number of stick insect sequences readily matched published data using this approach [34]. The longest assembled transcript from both species was Twitchin as expected of mRNA derived from insect muscle tissue [35]. No human contamination was detected (S3 and S4 Tables). Our preliminary functional assessment using eggNOG found orthologous groups for 52.77% of the transcript assemblies (Table 3). For *Acanthoxyla geisovii* 1446 transcripts (55.19%) and for *Clitarchus hookeri* 1294 (50.31%) transcripts were assigned to functional groups. The individual eggNOG hits for the two species are available at http://evolves.massey.ac.nz/DNA_Toolkit.htm (Tables “domainSummary_Ac.txt” and “domainSummary_Cl.txt”). There was a unique set of 127 combinations of Cluster of Orthologous Groups (COG) codes in the artNOG dataset. Of these, 26 were the single category codes (18,077 of 18,837 (95.97%)) and the remaining 101 were non-single category codes (760 of 18,837 (4.23%)), indicating that nearly all the annotations were categorised into single category codes. An overview of the functional categories found from the assembled transcripts set was obtained using the COG classifications. In comparison to the full artNOG set, the stick insect transcript assemblies showed about 4-fold underrepresentation of categories L (Replication, recombination and repair), D (Cell cycle control, cell division, chromosome partitioning) and K (Transcription), and >4-fold overrepresentation of J (Translation, ribosomal structure and biogenesis), Z (Cytoskeleton) and C (Energy production and conversion) (Table 3). A higher frequency of genes involved in the cytoskeleton and energy production in our transcript assemblies than in the arthropod database is compatible with the source of mRNA being leg muscle.

**Table 3:** Functional groups of assembled transcripts from stick insect cDNA inferred from similarity to an arthropod dataset (Reference) using evolutionary genealogy of groups: Non-supervised Orthologous Groups classification system (eggNOG). COG = Cluster of Orthologous Groups.

COG		Reference	Reference fraction	*Clitarchus*transcripts	*Clitarchus*fraction	*Acanthoxyla*transcripts	*Acanthoxyla*fraction
S	Function unknown	8136	0.4319	296	0.2287	306	0.2116
T	Signal transduction mechanisms	1733	0.092	128	0.0989	131	0.0906
K	Transcription	1360	0.0722	44	0.034	43	0.0297
O	Posttranslational modification, protein turnover, chaperones	1122	0.0596	129	0.0997	140	0.0968
	non-singleCOG	760	0.0423	46	0.0356	47	0.0327
G	Carbohydrate transport and metabolism	631	0.0335	45	0.0348	44	0.0304
U	Intracellular trafficking, secretion, and vesicular transport	535	0.0284	36	0.0278	39	0.027
E	Amino acid transport and metabolism	503	0.0267	23	0.0178	35	0.0242
I	Lipid transport and metabolism	486	0.0258	46	0.0355	54	0.0373
A	RNA processing and modification	438	0.0233	32	0.0247	44	0.0304
J	Translation, ribosomal structure and biogenesis	416	0.0221	121	0.0935	142	0.0982
C	Energy production and conversion	395	0.021	134	0.1036	152	0.1051
P	Inorganic ion transport and metabolism	394	0.0209	32	0.0247	29	0.0201
Z	Cytoskeleton	378	0.0201	105	0.0811	131	0.0906
L	Replication, recombination and repair	374	0.0199	4	0.0031	6	0.0041
Q	Secondary metabolites biosynthesis, transport and catabolism	301	0.016	16	0.0124	31	0.0214
D	Cell cycle control, cell division, chromosome partitioning	195	0.0104	8	0.0062	4	0.0028
F	Nucleotide transport and metabolism	149	0.0079	2	0.0015	6	0.0041
W	Extracellular structures	131	0.007	21	0.0162	25	0.0173
B	Chromatin structure and dynamics	121	0.0064	5	0.0039	7	0.0048
H	Coenzyme transport and metabolism	107	0.0057	4	0.0031	11	0.0076
M	Cell wall/membrane/envelope biogenesis	74	0.0039	9	0.007	9	0.0062
V	Defense mechanisms	73	0.0039	7	0.0054	8	0.0055
N	Cell motility	13	0.0007	1	0.0008	1	0.0007
Y	Nuclear structure	12	0.0006	0	0	1	0.0007
	Total	18837		1294		1446	

A hybrid genome is expected to have higher heterozygosity than a parental genome. Thus we determined how many loci (transcript assemblies) contained no sequence variation (Single Nucleotide Polymorphism; SNPs) when raw reads were mapped back to the assembled transcripts compiled from the respective genome (Fig 2). The putative parental species *Clitarchus hookeri* had 1900/2572 (73.87%) loci lacking SNPs, whereas the putative hybrid *Acanthoxyla geisovii* had only 728/2620 (28.3%) of loci without SNPs. The low level of allelic variation within *Clitarchus hookeri* was evident in a frequency analysis (data not shown).

A parental genome is expected to nest within the diversity present in the hybrid genome. Thus when the putative hybrid (*Acanthoxyla geisovii*) reads were mapped back to the gene sequences from the putative parent (*Clitarchus hookeri*) we expected a similar proportion of loci to be homozygous as observed within the putative hybrid (28% SNP free loci). Our data did not meet these expectations as the proportion of homozygous loci was significantly lower (568/2572; 22.1%; chi-squared p < 0.0001), however, similarity will naturally decline over time since hybridisation. When sequence reads from the putative parent (*Clitarchus hookeri*) were mapped back to the assembled transcripts from the putative hybrid (*Acanthoxyla geisovii*) there were fewer SNP free loci than expected (456/2620; 17.4%; chi-squared p < 0.0001). This might be a product of the triploid genome of *Acanthoxyla* (Myers *et al*. 2013) and could be assessed with additional taxon sampling.

Repeating these mapping procedures using only putative protein coding sequences (transcript assemblies passing a 48% GC content threshold), produced in near identical results except for a reduction in the size of density peaks at 0 SNPs per nucleotide, and a general shift of the *Clitarchus hookeri* reads vs. *Acanthoxyla geisovii* transcript assembly curve on the x-axis towards slightly larger SNPs per nucleotide densities (S2 Fig).

**Fig 4:**
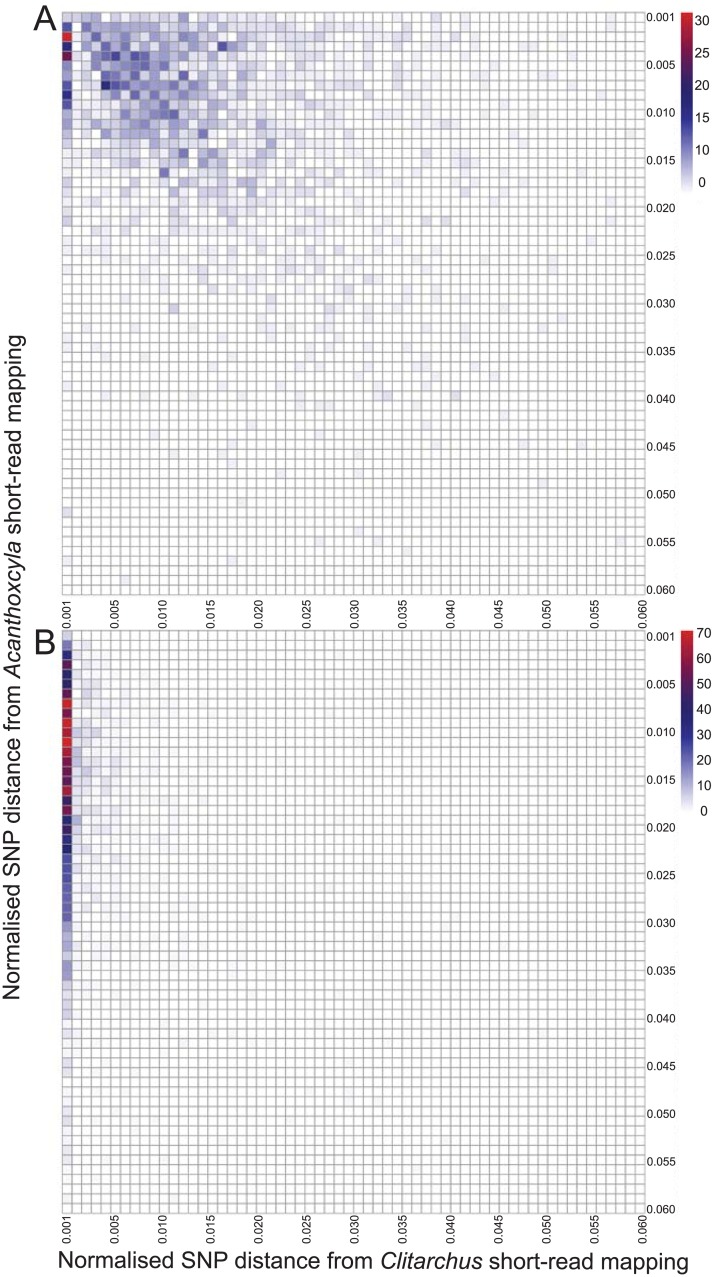
A similar level of genetic divergence within a hybrid stick insect genome as is observed between parental alleles when alleles are compared. Heat maps of sequence similarity between short-read cDNA sequences mapped to transcript assemblies. (A) *Acanthoxyla* transcript assemblies with *Clitarchus* reads (upper right) and *Acanthoxyla* reads (lower left). (B) *Clitarchus* transcript assemblies with *Clitarchus* reads (upper right) and *Acanthoxyla* reads (lower left).

The degree to which the sequences of alleles differed within a hybrid represents the genetic divergence of the two parental species. Therefore, allelic diversity between the maternal alleles and the paternal alleles can be observed within a single hybrid genome. This leads to the prediction that the difference between *Acanthoxyla* and *Clitarchus* alleles will be similar to the amount of allelic difference seen within *Acanthoxyla*, if *Clitarchus* is a parental taxon. By examining the number of SNPs as a proportion of the transcript assembly length we observed that alleles differed by a similar amount between the two stick insect genomes as they did within the putative hybrid (*Acanthoxyla*) genome (Fig 4), as expected. The symmetry of the heat map results from the similarity of sequence divergence within the hybrid and between the parent and hybrid alleles. As the divergence between the two subgenomes of the hybrid is higher than the diversity within a single parent (Fig 3b), we are able to distinguish the difference between alleles and paralogs.

## Discussion

### Are hybrids important?

Hybrid genomes are abundant and result from a range of processes; yeast fuse without sex [36], plant chimaeras are produced from grafting [37], and even some viral genomes appear to be the product of recombination between DNA and RNA viruses [38]. More than 70% of angiosperms are neo- or paleo-polyploids of which allopolyploids are most numerous [2], and the ancestor of vertebrates was involved in a whole genome duplication event [39]. So there is no doubt that many lineages are reticulate and that further tests of hybridisation are needed.

The phenotypic effects of hybridisation are many and varied and the role hybridisation plays in generating and reducing biodiversity is much debated [40, 41]. Furthermore, hybrid vigor has been associated with invasive species [42, 43], and conservation issues [44]. Thus the role and analysis of hybrid genomes are increasingly in the spotlight. The methods presented here provide the potential to explore any non-model organisms, even where ploidy level variation exists, as we have demonstrated by comparing diploid and triploid lineages. Distinguishing between paralogs and alleles is essential for downstream analyses, but where the divergence between two subgenomes of the hybrid is higher than the diversity within a single parent, then this is possible [45]. Artificial construction of hybrids, and recent natural hybrids reveal that alterations in gene expression can occur quickly due to many factors [46, 47], however, although this leads to important phenotypic variation it does not weaken our ability to test hybrid hypotheses using NGS. During manual curation of a subset of our data we found that expression levels as inferred from read coverage were strongly linked to ploidy level. A hybrid origin hypothesis sets up two testable predictions: 1. The putative hybrid will have greater allelic diversity than related non-hybrids; 2. Alleles of the parental taxa will co-occur in the genome of the hybrid. Both these predictions were met when we examine >2,500 loci expressed in *Acanthoxyla geisovii* (putative hybrid) and *Clitarchus hookeri* (putative paternal taxon).

### Stick insect evolution

Stick insects produce successful hybrids because they readily reproduce via parthenogenesis, simultaneously providing both potential fitness advantage over parental taxa and reproductive isolation from those parents [19, 48–50]. Diverse reproductive mechanisms are employed by stick insects, including both hybridogenesis and androgenesis, where just one parental genome is transmitted to offspring without recombination, resulting in hemiclonal inheritance of maternal or paternal genotypes [21]. In addition, parthenogenesis with or without recombination (automixis, thelytoky) is common; [21, 49], and each stick insect taxon may include numerous independent origins of parthenogenetic lineages [3, 20, 49, 51, 52]. The genome of the stick insect *Acanthoxyla geisovii* fits our predictions of a hybrid, containing high allelic diversity and sharing alleles with its putative paternal taxon, *Clitarchus hookeri*. Although no males have been described from *Acanthoxyla*, eight morphologically distinct species are recognized. The genus may have multiple origins given the variation detected in nuclear markers from the full range of morphological diversity [8, 15]. However, the species do not form monophyletic clades, as expected of clonal taxa [18], and possibly much (or all) of the morphological variation arises from recombination within the hybrid genome (automixis). The distinctive character combinations could also result from loss of heterozygosity during the transition from triploid to diploid [18]. Alternatively, multiple hybridisation events between different *Acanthoxyla* lineages and different *Clitarchus* lineages might have been involved in generating diversity. Sampling across the range of *Acanthoxyla* phenotypes using multiple markers will help resolve this question. Variation is the indispensable basis of evolution but males may not be needed to generate variation in this system due to the allelic diversity we have observed within the hybrid genome.

## Supporting Information

S1 Fig Open reading frame (ORF) prediction and GC content from stick insect mRNA. (DOCX)

S2 Fig The majority of transcript assemblies generated from cDNA from non-model organisms will find no match using BLAST searches against non-redundant (nr) protein databases, as in this example of two stick insects. Those transcript assemblies that have matches are predominantly from well resourced insect species.
(DOCX)

S3 Fig Kernel density plot generated for the GC rich subset of data (48% or more). Sequence divergence of stick insect protein coding DNA (measured by SNP density per nucleotide) observed when reads were mapped to loci (transcript assemblies). Putative parental genome (*Clitarchus hookeri*) contains many loci with no or low allelic diversity.
(DOCX)

S1 Table Short reads of cDNA from two New Zealand stick insects was assembled with a variety of different settings and software. Assembly statistics for assembly combinations, the number of contigs, and overall contig length for each species and *de novo* assembler grouped by kmer and data trim type.
(DOCX)

S2 Table After running custom scripts to generate a unique subset of contigs for each stick insect species, the number of sequences was greatly reduced, as shown. It should be noted that this process reduced the number of sequences contained within longer sequences; there was no reduction for any sequences that overlapped any other sequence.
(DOCX)

S3 Table The identity of transcript assemblies from *Acanthoxyla geisovii* was assessed by a BLAST homology search in which 13.3% matched sequences in the National Center for Biotechnology Information (NCBI) non-redundant (nr) protein database. (PDF)

S4 Table The identity of transcript assemblies from *Clitarchus hookeri* was assessed by a BLAST homology search in which 13.3% matched sequences in the National Center for Biotechnology Information (NCBI) non-redundant (nr) protein database. (PDF)
