# Supplementary material for: Sticky Genomes: Using NGS Evidence to Test Hybrid Speciation Hypotheses
Source: PLoS One. 2016 May 17;11(5):e0154911. doi: 10.1371/journal.pone.0154911 (PMC4871368; doi:10.1371/journal.pone.0154911)
Supplement: S1 Fig — (DOCX) [file pone.0154911.s001.docx]

**S1 Fig**. Open reading frame (ORF) prediction and GC content calculation indicated a positive relationship between ORF length and GC content from stick insect mRNA.
