# Supplementary material for: Sticky Genomes: Using NGS Evidence to Test Hybrid Speciation Hypotheses
Source: PLoS One. 2016 May 17;11(5):e0154911. doi: 10.1371/journal.pone.0154911 (PMC4871368; doi:10.1371/journal.pone.0154911)
Supplement: S2 Fig — Those transcript assemblies that have matches are predominantly from well resourced insect species. (DOCX) [file pone.0154911.s002.docx]

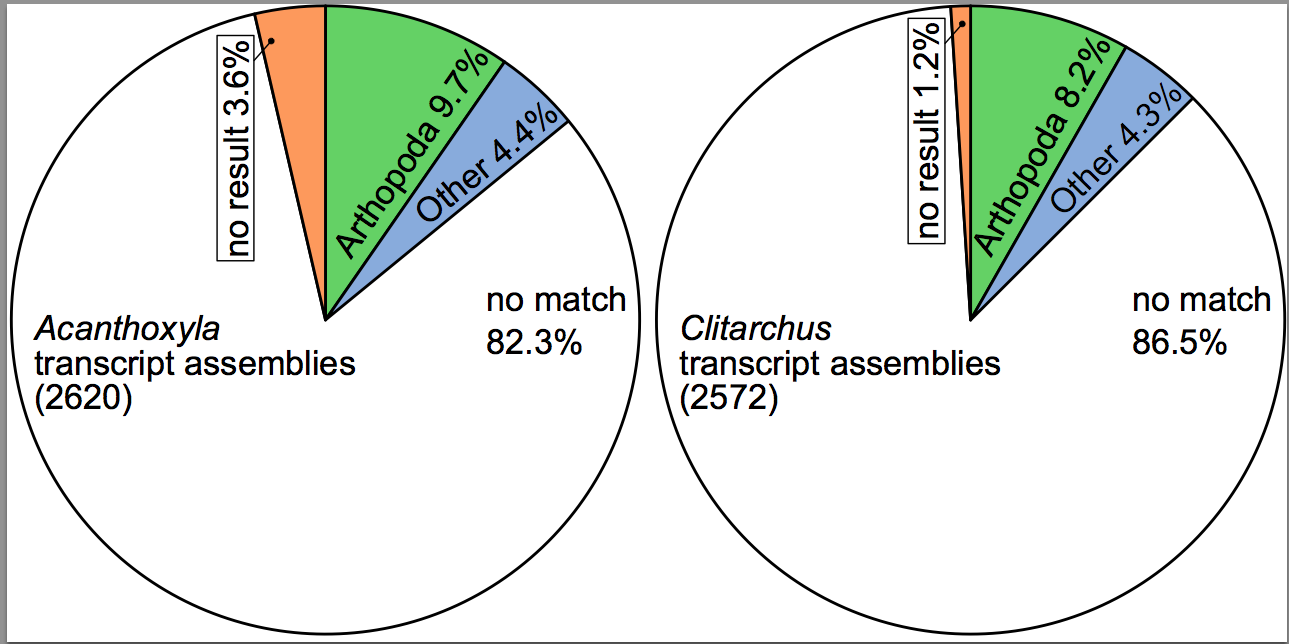


**S2 Fig**. The majority of transcript assemblies generated from cDNA from non-model organisms will find no match using BLAST searches against non-redundant (nr) protein databases, as in this example of two stick insects. Those transcript assemblies that have matches are predominantly from well resourced insect species.
