# Supplementary material for: Sticky Genomes: Using NGS Evidence to Test Hybrid Speciation Hypotheses
Source: PLoS One. 2016 May 17;11(5):e0154911. doi: 10.1371/journal.pone.0154911 (PMC4871368; doi:10.1371/journal.pone.0154911)
Supplement: S3 Fig — Sequence divergence of stick insect protein coding DNA (measured by SNP density per nucleotide) observed when reads were mapped to loci (transcript assemblies). Putative parental genome (Clitarchus hookeri) contains many loci with no or low allelic diversity. (DOCX) [file pone.0154911.s003.docx]

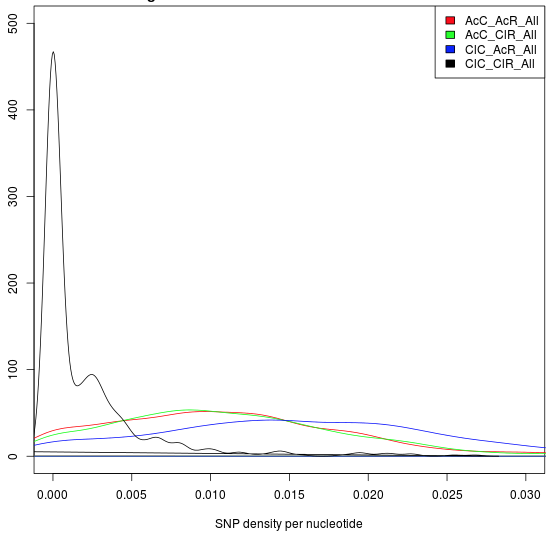


**S3 Fig.** Kernel density plot generated for the GC rich subset of data (48% or more). Sequence divergence of stick insect protein coding DNA (measured by SNP density per nucleotide) observed when reads were mapped to loci (transcript assemblies). Putative parental genome (*Clitarchus hookeri*) contains many loci with no or low allelic diversity. SNPs detected in less than 10% of the short reads were ignored. Only the longest assembled transcripts generated per cluster were included.
