# Supplementary material for: Sticky Genomes: Using NGS Evidence to Test Hybrid Speciation Hypotheses
Source: PLoS One. 2016 May 17;11(5):e0154911. doi: 10.1371/journal.pone.0154911 (PMC4871368; doi:10.1371/journal.pone.0154911)
Supplement: S1 Table — Assembly statistics for assembly combinations, the number of contigs, and overall contig length for each species and de novo assembler grouped by kmer and data trim type. (DOCX) [file pone.0154911.s004.docx]

**Table S1.** Short reads of cDNA from two New Zealand stick insects was assembled with a variety of different settings and software. Assembly statistics for assembly combinations, the number of contigs, and overall contig length for each species and *de novo* assembler grouped by kmer and data trim type

| Assembler | Isolate | trimType | Sum (real length) | Number of Contigs |
| --- | --- | --- | --- | --- |
| Velvet | r_Ag_0.001 | Trim2 | 7415378 | 20911 |
| Velvet | r_Ag_0.001 | Plain | 7245788 | 20633 |
| Velvet | r_Ag_0.003 | Trim2 | 15132749 | 43795 |
| Velvet | r_Ag_0.003 | Plain | 14610745 | 43765 |
| Velvet | r_Ag_0.01 | Trim2 | 20283291 | 58802 |
| Velvet | r_Ag_0.01 | Plain | 19130865 | 58008 |
| Velvet | r_Cl_0.001 | Trim2 | 10373787 | 25746 |
| Velvet | r_Cl_0.001 | Plain | 10173697 | 26002 |
| Velvet | r_Cl_0.003 | Trim2 | 20092413 | 50203 |
| Velvet | r_Cl_0.003 | Plain | 19463617 | 50977 |
| Velvet | r_Cl_0.01 | Trim2 | 25724284 | 64816 |
| Velvet | r_Cl_0.01 | Plain | 24649769 | 64826 |
| ABySS | r_Ag_0.001 | Trim2 | 7670007 | 23715 |
| ABySS | r_Ag_0.001 | Plain | 7345425 | 23419 |
| ABySS | r_Ag_0.003 | Trim2 | 15694227 | 49876 |
| ABySS | r_Ag_0.003 | Plain | 14411748 | 48218 |
| ABySS | r_Ag_0.01 | Trim2 | 21114379 | 67316 |
| ABySS | r_Ag_0.01 | Plain | 19006698 | 64468 |
| ABySS | r_Cl_0.001 \| | Trim2 | 11103757 | 30836 |
| ABySS | r_Cl_0.001 | Plain | 10687167 | 31275 |
| ABySS | r_Cl_0.003 | Trim2 | 21763811 | 61223 |
| ABySS | r_Cl_0.003 | Plain | 20440099 | 61659 |
| ABySS | r_Cl_0.01 | Trim2 | 25205573 | 68479 |
| ABySS | r_Cl_0.01 | Plain | 24569547 | 73832 |
