# Supplementary material for: Sticky Genomes: Using NGS Evidence to Test Hybrid Speciation Hypotheses
Source: PLoS One. 2016 May 17;11(5):e0154911. doi: 10.1371/journal.pone.0154911 (PMC4871368; doi:10.1371/journal.pone.0154911)
Supplement: S2 Table — It should be noted that this process reduced the number of sequences contained within longer sequences; there was no reduction for any sequences that overlapped any other sequence. (DOCX) [file pone.0154911.s005.docx]

**Table S2.** After running custom scripts to generate a unique subset of contigs for each stick insect species, the number of sequences was greatly reduced, as shown. It should be noted that this process reduced the number of sequences contained within longer sequences; there was no reduction for any sequences that overlapped any other sequence.

| \| Assember \| isolate \| Trim type \| Sun (real length) \| Number of contigs \| \| --- \| --- \| --- \| --- \| --- \| \| Velvet \| r_Ag_0.001 \| Trim2 \| 4990293 \| 13405 \| \| Velvet \| r_Ag_0.001 \| Plain \| 4867499 \| 13220 \| \| Velvet \| r_Ag_0.003 \| Trim2 \| 9009551 \| 24390 \| \| Velvet \| r_Ag_0.003 \| Plain \| 8949783 \| 25321 \| \| Velvet \| r_Ag_0.01 \| Trim2 \| 11540673 \| 30985 \| \| Velvet \| r_Ag_0.01 \| Plain \| 11219103 \| 31996 \| \| Velvet \| r_Cl_0.001 \| Trim2 \| 5665790 \| 13453 \| \| Velvet \| r_Cl_0.001 \| Plain \| 5591389 \| 13603 \| \| Velvet \| r_Cl_0.003 \| Trim2 \| 9216770 \| 21654 \| \| Velvet \| r_Cl_0.003 \| Plain \| 9069131 \| 22176 \| \| Velvet \| r_Cl_0.01 \| Trim2 \| 11105307 \| 26169 \| \| Velvet \| r_Cl_0.01 \| Plain \| 10906917 \| 26702 \| \| ABySS \| r_Ag_0.001 \| Trim2 \| 3329004 \| 9542 \| \| ABySS \| r_Ag_0.001 \| Plain \| 3267675 \| 9696 \| \| ABySS \| r_Ag_0.003 \| Trim2 \| 5905588 \| 17308 \| \| ABySS \| r_Ag_0.003 \| Plain \| 5707277 \| 17772 \| \| ABySS \| r_Ag_0.01 \| Trim2 \| 7451179 \| 21806 \| \| ABySS \| r_Ag_0.01 \| Plain \| 7147612 \| 22510 \| \| ABySS \| r_Cl_0.001 \| Trim2 \| 3290796 \| 8156 \| \| ABySS \| r_Cl_0.001 \| Plain \| 3327741 \| 8751 \| \| ABySS \| r_Cl_0.003 \| Trim2 \| 5481835 \| 13574 \| \| ABySS \| r_Cl_0.003 \| Plain \| 5533108 \| 14806 \| \| ABySS \| r_Cl_0.01 \| Trim2 \| 6538708 \| 15607 \| \| ABySS \| r_Cl_0.01 \| Plain \| 6741112 \| 17861 \| |
| --- | --- | --- | --- | --- | --- | --- | --- | --- | --- | --- | --- | --- | --- | --- | --- | --- | --- | --- | --- | --- | --- | --- | --- | --- | --- | --- | --- | --- | --- | --- | --- | --- | --- | --- | --- | --- | --- | --- | --- | --- | --- | --- | --- | --- | --- | --- | --- | --- | --- | --- | --- | --- | --- | --- | --- | --- | --- | --- | --- | --- | --- | --- | --- | --- | --- | --- | --- | --- | --- | --- | --- | --- | --- | --- | --- | --- | --- | --- | --- | --- | --- | --- | --- | --- | --- | --- | --- | --- | --- | --- | --- | --- | --- | --- | --- | --- | --- | --- | --- | --- | --- | --- | --- | --- | --- | --- | --- | --- | --- | --- | --- | --- | --- | --- | --- | --- | --- | --- | --- | --- | --- | --- | --- | --- | --- |
