# Supplementary material for: Sticky Genomes: Using NGS Evidence to Test Hybrid Speciation Hypotheses
Source: PLoS One. 2016 May 17;11(5):e0154911. doi: 10.1371/journal.pone.0154911 (PMC4871368; doi:10.1371/journal.pone.0154911)
Supplement: S3 Table — (PDF) [file pone.0154911.s006.pdf]

No.	Seq. name	Seq. decription	length	hits	min. eValue	sim. Mean
3	Ac_clus100003_Contig1	bombus impatiens hypothetical loc100743641 miscrna	2972	4	0.00E+00	68.50%
9	Ac_clus100009_Contig1	pediculus humanus corporis mrna	4208	2	0.00E+00	68.50%
11	Ac_clus100011_Contig1	drosophila ananassae gf24936 (dana\gf24936) mrna	3144	3	0.00E+00	68.33%
12	Ac_clus100012_Contig1	aedes aegypti mitochondrial partial mrna	1893	15	0.00E+00	70.13%
16	Ac_clus100016_Contig1	schistocerca gregaria atp-synthase subunit beta complete cds	2069	20	0.00E+00	79.90%
19	Ac_clus100019_Contig1	cavia porcellus oxoglutarate dehydrogenase-like transcript variant mrna	2063	17	0.00E+00	71.35%
24	Ac_clus100024_Contig1	anopheles gambiae pest agap002564-pe complete cds	1538	20	0.00E+00	73.45%
25	Ac_clus100025_Contig1	aedes aegypti eukaryotic translation elongation factor partial mrna	1473	20	0.00E+00	75.85%
27	Ac_clus100027_Contig1	locusta migratoria gapdh complete cds	1426	20	0.00E+00	75.05%
28	Ac_clus100028_Contig1	drosophila melanogaster alpha actinin transcript variant mrna	2695	20	0.00E+00	79.60%
32	Ac_clus100032_Contig1	clitarchus hookeri isolate -2 28s ribosomal rna partial sequence	2223	20	0.00E+00	97.85%
36	Ac_clus100036_Contig1	acyrthosiphon pisum probable aconitate mitochondrial-like mrna	1365	2	3.50E-93	72.00%
37	Ac_clus100037_Contig1	acyrthosiphon pisum twitchin-like mrna	2091	1	5.50E-150	66.00%
38	Ac_clus100038_Contig1	coptotermes formosanus clone cfsni164 heavy subunit complete cds	969	2	5.10E-45	66.00%
39	Ac_clus100039_Contig1	dasypus novemcinctus ca++ cardiac fast twitch 1 transcript variant mrna	2070	20	0.00E+00	74.05%
42	Ac_clus100042_Contig1	papilio xuthus mrna for beta- complete sequence id: px-0214	1542	20	0.00E+00	81.50%
43	Ac_clus100043_Contig1	gryllus bimaculatus gbcontig29003	1054	1	7.30E-50	74.00%
48	Ac_clus100048_Contig1	tribolium castaneum bmkettin mrna	1574	1	0.00E+00	71.00%
50	Ac_clus100050_Contig1	drosophila pseudoobscura pseudoobscura ga21679 (dpse\ga21679) transcript variant mrna	1384	8	1.30E-174	73.75%
52	Ac_clus100052_Contig1	tribolium castaneum cg32019-pa mrna	2065	1	0.00E+00	69.00%
54	Ac_clus100055_Contig1	drosophila melanogaster ferritin 1 heavy chain homologue transcript variant mrna	1083	20	9.80E-55	77.70%
59	Ac_clus100054_Contig1	tribolium castaneum cg32019-pa mrna	1555	1	9.50E-152	68.00%
63	Ac_clus100063_Contig1	diaphorina citri clone whdc1630 atp synthase gamma subunit partial cds	1328	1	7.60E-32	78.00%
64	Ac_clus100064_Contig1	culex quinquefasciatus glutamate mrna	1784	2	0.00E+00	72.50%
69	Ac_clus100069_Contig1	tribolium castaneum darkener of apricot cg33553-pg mrna	1851	2	4.00E-170	71.50%
76	Ac_clus100076_Contig1	rattus norvegicus high mobility group protein b1-like mrna	1097	20	6.20E-70	70.05%
77	Ac_clus100077_Contig1	cucumis sativus 60s ribosomal protein l23a-like mrna	864	8	2.70E-54	77.13%
79	Ac_clus100079_Contig1	argyrotaenia alisellana enolase partial cds	1217	20	0.00E+00	73.15%
84	Ac_clus100085_Contig1	drosophila melanogaster clone complete sequence	1529	20	0.00E+00	80.10%
86	Ac_clus100087_Contig1	megachile rotundata twitchin-like mrna	3511	1	0.00E+00	68.00%
90	Ac_clus100083_Contig1	anasa tristis arginine kinase complete cds	1022	20	3.00E-162	76.05%
91	Ac_clus100092_Contig1	maylandia zebra catalase-like mrna	1262	16	0.00E+00	72.56%
97	Ac_clus100098_Contig1	anopheles gambiae pest agap002154-pb partial cds	1179	2	3.30E-61	71.00%
101	Ac_clus100101_Contig1	mustela putorius furo calreticulin transcript variant mrna	1371	11	0.00E+00	75.64%
105	Ac_clus100105_Contig1	nasonia vitripennis peptidyl-prolyl cis-trans isomerase- transcript variant 2 mrna	1048	20	1.80E-95	74.30%
106	Ac_clus100106_Contig1	helicoverpa armigera heat shock protein 90 complete cds	1298	20	0.00E+00	74.75%
113	Ac_clus100113_Contig1	gryllus bimaculatus gbcontig29225	1097	1	1.90E-19	65.00%
114	Ac_clus100114_Contig1	aspergillus oryzae rib40 polyadenylate-binding cytoplasmic and mrna	1568	11	0.00E+00	72.73%
117	Ac_clus100117_Contig1	drosophila melanogaster heat shock protein cognate 4 (hsc70-4) transcript variant mrna	1076	20	0.00E+00	78.50%
118	Ac_clus100118_Contig1	nematostella vectensis protein partial mrna	1820	1	1.50E-42	75.00%
125	Ac_clus100121_Contig1	jakoba bahamiensis strain atcc 50695 complete genome	916	2	1.10E-78	69.00%
127	Ac_clus100127_Contig1	drosophila persimilis gl12270 (dper\gl12270) mrna	1338	1	3.10E-24	77.00%
128	Ac_clus100128_Contig1	bos taurus eukaryotic translation elongation factor 1 delta transcript variant 1 complete alternatively spliced	790	5	7.50E-42	70.00%
129	Ac_clus100129_Contig1	nasonia vitripennis muscle m-line assembly protein unc-89-like mrna	1650	2	1.30E-156	67.00%
130	Ac_clus100131_Contig1	pan troglodytes ribosomal protein l8 mrna	863	20	2.40E-175	75.55%
139	Ac_clus100140_Contig1	aedes aegypti mitochondrial processing peptidase beta subunit partial mrna	1495	5	0.00E+00	72.00%
140	Ac_clus100139_Contig1	ovis aries 60s ribosomal protein l10a-like mrna	837	20	6.10E-151	76.65%
141	Ac_clus100141_Contig1	lachancea thermotolerans cbs 6340 klth0b08514p complete cds	913	4	2.20E-30	80.00%
147	Ac_clus100147_Contig1	aedes aegypti eukaryotic translation elongation factor partial mrna	954	20	0.00E+00	77.40%
148	Ac_clus100148_Contig1	drosophila melanogaster ribosomal protein s2 transcript variant mrna	958	20	0.00E+00	78.65%

150	Ac_clus100150_Contig1	gryllus bimaculatus gbcontig22796	1585	1	0.00E+00	79.00%
151	Ac_clus100151_Contig1	drosophila melanogaster calcium atpase at 60a (ca-p60a) transcript variant mrna	1343	20	0.00E+00	79.75%
159	Ac_clus100160_Contig1	agathemera crassa nuclear 18s rrna gene	1502	20	0.00E+00	98.65%
160	Ac_clus100162_Contig1	marsupenaeus japonicus isolate 02 actin complete cds	1132	20	0.00E+00	86.00%
163	Ac_clus100164_Contig1	tentyria rotundata partial mp20 gene for muscular protein specimen voucher bmnh exons 1-2	660	20	1.10E-114	71.35%
165	Ac_clus100166_Contig1	papilio xuthus mrna for muscle myosin heavy complete clone: fsg31	1191	20	0.00E+00	77.25%
166	Ac_clus100167_Contig1	aedes albopictus clone al_249 translationally controlled tumor protein complete cds	781	6	1.70E-43	67.33%
167	Ac_clus100168_Contig1	bombyx mandarina strain suzhou paramyosin complete cds	1470	4	0.00E+00	75.25%
168	Ac_clus100169_Contig2	delphacodes kuscheli actin partial cds	457	20	3.90E-150	85.25%
172	Ac_clus100173_Contig1	fenneropenaeus chinensis glucose-regulated protein 78 complete cds	1093	12	6.60E-152	73.75%
173	Ac_clus100174_Contig1	cicer arietinum t-complex protein 1 subunit epsilon-like mrna	954	1	2.50E-55	69.00%
174	Ac_clus100175_Contig1	candida albicans sc5314 histone h3 complete cds	1170	20	1.30E-91	77.45%
175	Ac_clus100176_Contig1	papilio xuthus mrna for adp ribosylation complete sequence id: px-0305	1518	20	2.80E-101	76.60%
177	Ac_clus100179_Contig1	megachile rotundata f-box lrr-repeat protein 7-like mrna	1003	2	0.00E+00	72.00%
180	Ac_clus100183_Contig1	ceratotherium simum simum nadh dehydrogenase	1012	3	5.40E-102	79.00%
191	Ac_clus100194_Contig1	hister apv-2005 mrna for ribosomal protein s23e (rps23e gene)	540	2	5.40E-86	75.00%
193	Ac_clus100196_Contig1	dendroctonus ponderosae whole genome shotgun sequence	455	2	2.80E-63	78.00%
196	Ac_clus100199_Contig1	papilio xuthus mrna for ribosomal protein complete sequence id: px-0053	906	3	5.40E-171	74.33%
197	Ac_clus100200_Contig2	gryllus bimaculatus gbcontig30723	600	3	2.10E-47	83.00%
200	Ac_clus100204_Contig1	acyrthosiphon pisum clone: full-insert cdna sequence based on ests (5 -est: 3 -est aci2aaf1yd14bbm1)	1370	14	4.90E-142	70.07%
205	Ac_clus100210_Contig2	papilio xuthus mrna for cg6803- complete clone: psg25	757	1	4.50E-19	77.00%
210	Ac_clus100218_Contig1	aedes aegypti atpase subunit partial mrna	1001	20	2.40E-68	82.50%
212	Ac_clus100220_Contig1	phobaeticus serratipes mitochondrial complete genome	1141	14	1.20E-85	84.79%
215	Ac_clus100223_Contig1	arabidopsis thaliana chromosome complete sequence	758	20	0.00E+00	92.80%
216	Ac_clus100224_Contig1	drosophila miranda partial ga15129 strain 635ma	1882	8	4.00E-94	72.00%
217	Ac_clus100217_Contig1	ostrinia furnacalis strain acb- heat shock cognate 70 kda protein complete cds	569	20	3.80E-145	79.85%
224	Ac_clus100231_Contig1	gryllus bimaculatus gbcontig07471	542	1	2.60E-20	91.00%
232	Ac_clus100239_Contig1	aphanomyces euteiches cdna	1171	12	2.50E-56	71.08%
241	Ac_clus100243_Contig1	sorex araneus protein disulfide isomerase family member 3 transcript variant mrna	1151	3	3.60E-35	71.00%
248	Ac_clus100255_Contig1	drosophila ananassae gf18582 (dana\gf18582) mrna	738	1	9.10E-47	69.00%
250	Ac_clus100257_Contig1	sorex araneus nucleosome assembly protein 1-like 4 mrna	947	2	3.40E-28	72.50%
253	Ac_clus100260_Contig1	hyriopsis cumingii 37 kda laminin receptor complete cds	707	1	5.90E-87	73.00%
255	Ac_clus100262_Contig1	callithrix jacchus cytoplasmic 2- transcript variant 1 mrna	263	20	4.10E-58	84.00%
256	Ac_clus100263_Contig1	nasonia vitripennis 40s ribosomal protein s9-like mrna	699	1	2.80E-116	76.00%
257	Ac_clus100264_Contig1	helicoverpa armigera heat shock protein 90 complete cds	898	20	1.30E-172	79.00%
260	Ac_clus100267_Contig1	tetraodon nigroviridis full-length cdna	1222	20	0.00E+00	80.55%
261	Ac_clus100268_Contig1	helicoverpa armigera heat shock protein 90 complete cds	753	20	1.10E-153	76.85%
270	Ac_clus100277_Contig1	bacillus atticus partial genome	1093	20	6.20E-165	81.30%
271	Ac_clus100278_Contig2	carausius morosus 28s ribosomal rna partial sequence	1471	20	0.00E+00	88.10%
274	Ac_clus100281_Contig1	plutella xylostella 40 protein complete cds	557	20	3.50E-101	79.50%
275	Ac_clus100282_Contig1	kazachstania africana cbs 2517 chromosome complete genome	958	2	3.70E-15	72.00%
277	Ac_clus100284_Contig1	bombyx mori troponin isoform 2-like mrna	513	3	5.80E-60	75.00%
278	Ac_clus100285_Contig2	agathemera crassa 18s ribosomal rna partial sequence	917	20	0.00E+00	98.75%
280	Ac_clus100287_Contig1	monochamus alternatus adenine nucleotide translocase complete cds	1329	20	1.20E-130	72.50%
284	Ac_clus100291_Contig1	drosophila yakuba ge12016 (dyak\ge12016) mrna	1318	1	8.10E-57	69.00%
285	Ac_clus100292_Contig1	chrysomela tremulae ribosomal protein l7 complete cds	836	3	4.70E-108	73.00%
298	Ac_clus100305_Contig1	nasonia vitripennis camp-dependent protein kinase type i regulatory subunit-like partial mrna	1106	2	9.90E-169	74.50%
315	Ac_clus100322_Contig1	oreochromis niloticus succinate dehydrogenase	1368	7	0.00E+00	72.29%
330	Ac_clus100337_Contig1	biston betularia ribosomal protein l4 partial cds	1165	20	4.80E-135	69.15%
331	Ac_clus100338_Contig1	telmatoscopus amf-2003 ribosomal protein s6 partial cds	566	1	6.00E-54	71.00%
332	Ac_clus100339_Contig1	gesonula punctifrons 14-3-3 zeta partial alternatively spliced	1123	3	1.10E-41	79.33%

334	Ac_clus100341_Contig1	nasonia vitripennis muscle m-line assembly protein unc-89-like mrna	1361	2	1.50E-167	68.50%
335	Ac_clus100342_Contig1	bombus impatiens protein translation factor sui1 homolog mrna	466	20	8.80E-108	81.45%
336	Ac_clus100343_Contig1	callorhinchus milii clone 40s ribosomal protein s11 complete cds	488	13	6.70E-97	78.92%
338	Ac_clus100345_Contig1	ochotona princeps tyrosine 3-monooxygenase tryptophan 5-monooxygenase activation eta polypeptide mrna	786	3	7.50E-61	79.33%
340	Ac_clus100347_Contig1	drosophila simulans gd19909 (dsim\gd19909) mrna	417	20	6.90E-77	83.35%
344	Ac_clus100351_Contig1	aedes aegypti 60s ribosomal protein l23 partial mrna	508	13	8.50E-115	76.23%
348	Ac_clus100355_Contig1	gryllus bimaculatus gbcontig28204	1205	14	3.60E-124	72.86%
358	Ac_clus100366_Contig1	bos taurus isocitrate dehydrogenase 3 (nad+) beta transcript variant mrna	883	14	1.60E-82	72.86%
360	Ac_clus100367_Contig1	rana catesbeiana clone rcat-evr-518-049 40s ribosomal protein s16 complete cds	520	4	5.50E-92	76.00%
371	Ac_clus100380_Contig1	monodelphis domestica 60s ribosomal protein l26-like mrna	578	3	3.90E-88	75.33%
373	Ac_clus100382_Contig1	drosophila melanogaster cg14207 transcript variant mrna	767	18	2.70E-85	74.72%
374	Ac_clus100383_Contig1	drosophila pseudoobscura pseudoobscura ga16481 (dpse\ga16481) mrna	888	10	3.40E-167	80.40%
377	Ac_clus100386_Contig1	anopheles gambiae pest agap007135-pa complete cds	1236	5	0.00E+00	79.60%
379	Ac_clus100388_Contig1	leptynia hispanica isolate hucla8 elongation factor 1-alpha (ef1-alpha) partial cds	405	20	1.40E-161	90.25%
384	Ac_clus100393_Contig1	anolis carolinensis atp synthase subunit mitochondrial-like mrna	652	1	1.50E-11	77.00%
385	Ac_clus100394_Contig1	latimeria chalumnae ribosomal protein l5 partial cds	1047	1	3.50E-98	71.00%
386	Ac_clus100395_Contig1	dermacentor variabilis isolate 40 ribosomal protein l12 complete cds	675	1	5.20E-62	71.00%
391	Ac_clus100402_Contig1	drosophila pseudoobscura pseudoobscura ga19246 (dpse\ga19246) transcript variant mrna	732	7	6.10E-163	76.71%
398	Ac_clus100399_Contig1	locusta migratoria isolate ii-11 csp partial cds	556	4	2.10E-15	68.75%
399	Ac_clus100409_Contig1	ixodes scapularis cytochrome mrna	646	4	8.40E-72	80.00%
402	Ac_clus100413_Contig1	paratoxopoda depilis voucher su37 elongation factor-1 alpha partial cds	470	20	1.80E-135	81.95%
403	Ac_clus100414_Contig1	cryptocercus punctulatus elongation factor 1-alpha complete cds	730	20	1.50E-132	78.20%
408	Ac_clus100421_Contig1	helicoverpa armigera actin complete cds	763	20	0.00E+00	88.25%
417	Ac_clus100430_Contig1	aedes aegypti dead boxatp-dependent rna helicase partial mrna	357	13	2.20E-82	79.08%
426	Ac_clus100438_Contig1	mus musculus crl-1722 l5178y-r riken full-length enriched clone:i730086p06 product:chaperonin subunit 5 full insert sequence	942	20	6.50E-126	71.65%
431	Ac_clus100443_Contig1	ornithorhynchus anatinus 6- liver type-like mrna	676	2	2.10E-16	81.00%
449	Ac_clus100458_Contig1	plutella xylostella ryanodine receptor complete cds	443	13	2.70E-120	80.00%
453	Ac_clus100466_Contig1	oryza sativa japonica group chromosome 5 clone complete sequence	928	15	3.50E-110	75.80%
467	Ac_clus100482_Contig1	single read from an extremity of a full-length cdna clone made from anopheles gambiae total adult 5-prime end of clone fk0aac35ac08 of strain 6-9 of anopheles gambiae (african malaria mosquito)	808	20	4.80E-114	73.20%
470	Ac_clus100486_Contig1	gryllus bimaculatus gbcontig06695	1133	1	6.50E-13	81.00%
473	Ac_clus100489_Contig1	drosophila melanogaster s-adenosylmethionine synthetase (sam-s) transcript variant mrna	743	20	2.60E-85	71.15%
474	Ac_clus100490_Contig1	ictalurus furcatus clone cbzc3255 guanine nucleotide-binding protein subunit beta-2-like 1 complete cds	712	1	1.10E-127	76.00%
475	Ac_clus100491_Contig1	anopheles gambiae pest agap005960-pa complete cds	480	3	6.20E-53	71.00%
477	Ac_clus100493_Contig1	lethenteron camtschaticum rps3 mrna for ribosomal protein partial cds	811	8	7.70E-175	78.63%
478	Ac_clus100418_Contig1	drosophila melanogaster myosin heavy chain transcript variant mrna	1153	20	0.00E+00	80.35%
482	Ac_clus100494_Contig1	echinops telfairi nadh dehydrogenase flavoprotein 51kda transcript variant mrna	1194	20	0.00E+00	75.40%
484	Ac_clus100499_Contig1	papilio xuthus mrna for ribosomal protein complete sequence id: px-0166	436	2	2.90E-50	79.00%
488	Ac_clus100504_Contig1	tetraodon nigroviridis full-length cdna	618	20	4.50E-94	75.00%
493	Ac_clus100508_Contig1	drosophila melanogaster eukaryotic initiation factor 1a (eif-1a) transcript variant mrna	459	20	9.90E-82	76.70%
496	Ac_clus100511_Contig1	paracoccidioides lutzii pb01 40s ribosomal protein partial mrna	543	4	8.00E-46	75.00%
499	Ac_clus100514_Contig1	drosophila sechellia gm26300 (dsec\gm26300) mrna	622	14	1.10E-82	72.93%
508	Ac_clus100523_Contig1	drosophila simulans gd15763 (dsim\gd15763) mrna	973	2	1.00E-47	71.00%
514	Ac_clus100529_Contig1	acartia pacifica clone acapa-cdna37-1 60s ribosomal protein l15 complete cds	722	16	4.90E-88	70.44%

515	Ac_clus100530_Contig1	mustela putorius furo nucleoside diphosphate kinase chloroplastic-like transcript variant mrna	799	8	2.00E-55	72.38%
518	Ac_clus100533_Contig1	ornithorhynchus anatinus 40s ribosomal protein s10-like mrna	376	3	1.80E-58	78.33%
519	Ac_clus100534_Contig1	callithrix jacchus 60s ribosomal protein l27a-like mrna	511	12	6.60E-72	73.25%
521	Ac_clus100535_Contig1	riptortus pedestris mrna for ribosomal protein complete sequence id: rped-0117	303	1	2.50E-17	72.00%
541	Ac_clus100557_Contig1	nasonia vitripennis 60s ribosomal protein l18a- transcript variant 1 mrna	330	3	1.90E-76	77.67%
543	Ac_clus100556_Contig1	drosophila melanogaster ribosomal protein l3 transcript variant mrna	667	20	4.80E-151	75.90%
544	Ac_clus100558_Contig1	callorhinchus milii clone ribosomal protein l10 complete cds	730	20	1.20E-165	75.50%
549	Ac_clus100564_Contig1	gryllus bimaculatus gbcontig31584	595	1	8.80E-103	78.00%
550	Ac_clus100565_Contig1	phobaeticus serratipes mitochondrial complete genome	1715	20	7.60E-122	83.30%
561	Ac_clus100577_Contig1	bombus terrestris 60s ribosomal protein l9-like mrna	621	2	5.10E-106	74.50%
562	Ac_clus100578_Contig1	nilaparvata lugens heat shock cognate protein 70 complete cds	866	20	0.00E+00	78.75%
568	Ac_clus100584_Contig1	chrysomela tremulae ribosomal protein s7 complete cds	659	1	1.90E-105	77.00%
572	Ac_clus100590_Contig1	bombyx mori myosin light chain 2 complete cds	970	20	2.20E-157	78.70%
573	Ac_clus100588_Contig1	drosophila melanogaster myosin heavy chain transcript variant mrna	732	20	0.00E+00	84.60%
576	Ac_clus100589_Contig1	bacillus rossius 28s ribosomal rna partial sequence	876	20	0.00E+00	89.45%
579	Ac_clus100597_Contig1	drosophila melanogaster paramyosin transcript variant mrna	819	20	0.00E+00	77.65%
581	Ac_clus100598_Contig1	boechera divaricarpa isolate slw-d-d09 mrna sequence	595	20	2.20E-167	79.65%
582	Ac_clus100599_Contig1	drosophila melanogaster tropomyosin 2 transcript variant mrna	764	20	0.00E+00	79.05%
583	Ac_clus100600_Contig1	diabolocatantops pinguis beta-actin complete cds	364	20	1.70E-115	89.75%
586	Ac_clus100603_Contig1	drosophila melanogaster ryanodine receptor 44f (rya-r44f) transcript variant mrna	755	18	2.00E-175	76.39%
591	Ac_clus100609_Contig1	papilio xuthus mrna for vacuolar h	797	1	3.00E-72	69.00%
597	Ac_clus100615_Contig1	osmerus mordax clone omor-eva-511-147 tubulin alpha-1 chain complete cds	312	7	3.80E-72	77.71%
615	Ac_clus100633_Contig1	drosophila melanogaster serpentine transcript variant mrna	664	20	0.00E+00	85.95%
621	Ac_clus100641_Contig1	megachile rotundata uncharacterized loc100883123 mrna	757	5	9.40E-47	80.40%
627	Ac_clus100647_Contig1	locusta migratoria mrna for eukaryotic translation initiation factor 2 gamma subunit (eif2g gene)	583	3	2.50E-141	71.33%
645	Ac_clus100666_Contig1	leptospira biflexa serovar patoc strain patoc 1 chromosome complete sequence	1274	7	3.10E-106	71.71%
650	Ac_clus100671_Contig1	nasonia vitripennis transmembrane protein 35-like mrna	637	1	1.20E-25	67.00%
663	Ac_clus100684_Contig1	maconellicoccus hirsutus clone whmh2520 ribosomal protein l13 complete cds	756	1	1.10E-58	71.00%
669	Ac_clus100687_Contig1	heliothis virescens secreted protein acidic and rich in cysteine-like complete sequence	313	3	8.50E-68	76.00%
671	Ac_clus100694_Contig1	drosophila melanogaster clone complete sequence	1156	20	9.20E-43	86.05%
680	Ac_clus100699_Contig1	bos taurus peroxiredoxin 5 mrna	641	2	2.30E-28	67.50%
683	Ac_clus100703_Contig1	cnaphalocrocis medinalis ryanodine receptor complete cds	466	3	3.80E-68	70.33%
692	Ac_clus100713_Contig1	papilio xuthus mrna for ribosomal protein complete sequence id: px-0055	518	1	6.20E-104	80.00%
698	Ac_clus100720_Contig1	bombus terrestris 40s ribosomal protein s29-like mrna	407	1	6.30E-33	79.00%
699	Ac_clus100721_Contig1	ovis aries 40s ribosomal protein s15a-like mrna	510	20	3.40E-88	76.70%
700	Ac_clus100718_Contig1	clitarchus hookeri variant i internal transcribed spacer ribosomal rna and internal transcribed spacer complete sequence	935	20	0.00E+00	92.65%
703	Ac_clus100724_Contig1	drosophila melanogaster elongation factor 1alpha48d transcript variant mrna	466	20	1.40E-149	84.00%
713	Ac_clus100733_Contig1	setaria italica adp-ribosylation factor-like transcript variant mrna	547	20	1.00E-88	76.95%
715	Ac_clus100736_Contig1	drosophila ananassae gf12176 (dana\gf12176) mrna	614	2	2.10E-66	72.00%
719	Ac_clus100742_Contig1	monodelphis domestica glycogen muscle form-like mrna	745	2	1.20E-108	72.00%
725	Ac_clus100746_Contig1	mesocricetus auratus partial mrna for dihydrolipoamide dehydrogenase (dld gene)	540	1	1.20E-43	76.00%
726	Ac_clus100747_Contig1	aedes aegypti nascent polypeptide associated complex alpha subunit (nac alpha) partial mrna	647	20	4.10E-44	70.20%
732	Ac_clus100756_Contig1	bombyx mori genomic chromosome bac clone: complete sequence	306	20	1.60E-70	92.95%
733	Ac_clus100757_Contig1	megachile rotundata 40s ribosomal protein s28-like mrna	394	1	4.40E-41	79.00%
734	Ac_clus100758_Contig1	tribolium castaneum s-adenosylmethionine transcript variant 1 mrna	534	1	1.30E-17	95.00%

740	Ac_clus100764_Contig1	culex quinquefasciatus mrna	686	20	1.50E-138	82.55%
741	Ac_clus100765_Contig1	manduca sexta ribosomal protein l17 complete cds	586	2	1.50E-74	76.50%
742	Ac_clus100766_Contig1	cryptocercus punctulatus ribosomal protein s18 complete cds	624	5	1.20E-107	70.60%
744	Ac_clus100768_Contig1	zea mays loc100281135 (pco103560) mrna	836	2	1.80E-18	75.50%
745	Ac_clus100769_Contig1	homarus americanus troponin c isoform 2a complete cds	387	1	1.60E-27	75.00%
750	Ac_clus100774_Contig1	placozoa h4 eukaryotic translation initiation factor 5a complete cds	614	2	7.50E-47	82.00%
763	Ac_clus100787_Contig1	drosophila grimshawi gh25015 (dgri\gh25015) mrna	860	2	6.30E-37	69.00%
765	Ac_clus100789_Contig1	gryllus bimaculatus gbcontig30509	456	1	8.10E-121	83.00%
768	Ac_clus100792_Contig1	aedes aegypti troponin c partial mrna	632	15	2.40E-110	76.40%
769	Ac_clus100793_Contig1	drosophila melanogaster myosin heavy chain transcript variant mrna	637	20	0.00E+00	84.10%
770	Ac_clus100794_Contig1	megachile rotundata muscle lim protein mlp84b-like mrna	492	5	4.00E-68	79.00%
779	Ac_clus100804_Contig1	perkinsus marinus atcc 50983 mrna	461	20	1.40E-54	80.55%
780	Ac_clus100805_Contig1	gryllus bimaculatus gbcontig07916	542	1	2.60E-20	87.00%
781	Ac_clus100806_Contig1	ashbya gossypii atcc 10895 chromosome complete sequence	431	4	1.40E-41	73.75%
782	Ac_clus100807_Contig1	tetraodon nigroviridis full-length cdna	788	20	0.00E+00	77.90%
792	Ac_clus100817_Contig1	ochrogaster lunifer complete mitochondrial genome	268	20	5.10E-38	84.25%
796	Ac_clus100821_Contig1	megacrania alpheus adan mitochondrial complete genome	808	20	9.40E-60	83.45%
800	Ac_clus100825_Contig1	gryllus bimaculatus gbcontig05884	597	1	4.00E-31	77.00%
827	Ac_clus100852_Contig1	single read from an extremity of a full-length cdna clone made from anopheles gambiae total adult 3-prime end of clone fk0aaa9ab05 of strain 6-9 of anopheles gambiae (african malaria mosquito)	497	20	1.20E-87	74.50%
833	Ac_clus100858_Contig1	megachile rotundata ryanodine receptor 44f-like mrna	906	2	6.60E-151	72.50%
841	Ac_clus100866_Contig1	single read from an extremity of a full-length cdna clone made from anopheles gambiae total adult 5-prime end of clone fk0aaa12df06 of strain 6-9 of anopheles gambiae (african malaria mosquito)	751	17	3.90E-64	71.47%
854	Ac_clus100879_Contig1	tetraodon nigroviridis full-length cdna	492	2	3.10E-44	70.50%
863	Ac_clus100888_Contig1	dendroctonus ponderosae whole genome shotgun sequence	702	2	4.80E-50	76.00%
865	Ac_clus100890_Contig1	drosophila ananassae gf17649 (dana\gf17649) mrna	591	20	0.00E+00	84.50%
881	Ac_clus100906_Contig1	cavia porcellus dihydrolipoamide s-acetyltransferase mrna	752	1	8.20E-35	67.00%
889	Ac_clus100918_Contig1	salpingoeca atcc 50818 atp-dependent rna helicase dbp2 complete cds	448	3	4.40E-29	77.67%
892	Ac_clus100921_Contig1	drosophila melanogaster heat shock protein cognate 3 (hsc70-3) transcript variant mrna	739	20	4.70E-177	78.20%
893	Ac_clus100922_Contig1	nomascus leucogenys s-phase kinase-associated protein 1 mrna	863	20	1.80E-113	78.05%
899	Ac_clus100927_Contig1	oryctolagus cuniculus aldehyde dehydrogenase 1l1-like mrna	647	9	1.90E-105	69.22%
914	Ac_clus100942_Contig1	drosophila melanogaster ryanodine receptor 44f (rya-r44f) transcript variant mrna	654	17	7.50E-98	74.88%
919	Ac_clus100947_Contig1	drosophila melanogaster ryanodine receptor 44f (rya-r44f) transcript variant mrna	634	20	5.60E-150	80.15%
928	Ac_clus100956_Contig1	kazachstania naganishii cbs 8797 chromosome complete genome	536	2	1.30E-30	71.00%
940	Ac_clus100967_Contig1	anopheles gambiae pest agap003851-pa complete cds	393	16	3.30E-36	78.63%
947	Ac_clus100974_Contig1	salmo salar 6-phosphofructo-2-kinase fructose- -biphosphatase 2 mrna	484	1	3.20E-12	80.00%
951	Ac_clus100978_Contig1	gallus gallus citrate synthase mrna	514	1	1.00E-75	74.00%
956	Ac_clus100983_Contig1	gryllus bimaculatus gbcontig20780	644	1	5.70E-17	85.00%
957	Ac_clus100984_Contig1	arabidopsis thaliana ribulose bisphosphate small subunit ( ) complete cds	452	20	0.00E+00	96.00%
966	Ac_clus100993_Contig1	mya arenaria mitochondrial mortalin-2 precursor (mot-2) complete cds nuclear gene for mitochondrial product	779	7	2.60E-155	74.00%
967	Ac_clus100994_Contig1	maylandia zebra stress-70 mitochondrial-like transcript variant mrna	398	5	3.20E-49	71.60%
973	Ac_clus101001_Contig1	meleagris gallopavo counting factor associated protein d-like mrna	604	3	8.40E-40	68.33%
981	Ac_clus101009_Contig1	monosiga brevicollis mx1 protein monbrdraft_24772 complete cds	425	2	3.20E-43	71.00%
986	Ac_clus101014_Contig1	sus scrofa splicing factor u2af 26 kda subunit- transcript variant 1 mrna	582	4	7.60E-72	75.50%
999	Ac_clus101023_Contig1	anopheles gambiae pest agap012045-pa complete cds	728	20	2.30E-73	68.25%
1000	Ac_clus101029_Contig1	tribolium castaneum delta9-acyl- desaturase mrna	378	1	1.70E-14	75.00%
1001	Ac_clus101030_Contig1	drosophila melanogaster serpentine transcript variant mrna	552	20	5.10E-175	75.65%
1003	Ac_clus101032_Contig1	drosophila melanogaster gasp transcript variant mrna	214	17	5.10E-62	82.94%
1026	Ac_clus101060_Contig1	bombus impatiens peptidyl-prolyl cis-trans isomerase fkbp1a-like mrna	396	3	1.80E-52	77.00%
1028	Ac_clus101062_Contig1	drosophila melanogaster knockdown transcript variant mrna	689	10	3.20E-90	73.50%
1037	Ac_clus101072_Contig1	ixodes scapularis gtp-binding nuclear protein mrna	585	20	1.10E-101	77.35%

1052	Ac_clus101086_Contig1	drosophila melanogaster ryanodine receptor 44f (rya-r44f) transcript variant mrna	627	20	1.70E-118	72.25%
1054	Ac_clus101088_Contig1	trichechus manatus latirostris ribosomal protein l36 mrna	400	3	4.20E-35	80.33%
1080	Ac_clus101114_Contig1	papilio polytes mrna for ribosomal protein complete sequence id: pp-0021	252	6	6.20E-43	74.67%
1087	Ac_clus101121_Contig1	nasonia vitripennis hypothetical transcript variant 2 mrna	970	3	1.50E-178	76.67%
1088	Ac_clus101122_Contig1	saimiri boliviensis boliviensis 60s ribosomal protein l3-like misc_rna	495	1	2.50E-45	73.00%
1092	Ac_clus101126_Contig1	single read from an extremity of a full-length cdna clone made from anopheles gambiae total adult 3-prime end of clone fk0aaa29ca04 of strain 6-9 of anopheles gambiae (african malaria mosquito)	495	1	4.00E-30	79.00%
1102	Ac_clus101136_Contig1	meleagris gallopavo 60s ribosomal protein l3-like mrna	450	2	5.40E-66	74.50%
1105	Ac_clus101139_Contig1	gryllus bimaculatus gbcontig29054	426	4	1.30E-73	77.75%
1110	Ac_clus101144_Contig1	callorhinchus milii clone ribosomal protein l34 complete cds	356	14	2.20E-82	79.14%
1111	Ac_clus101145_Contig1	arabidopsis lyrata lyrata mrna	404	20	0.00E+00	98.90%
1112	Ac_clus101146_Contig1	neohirasea japonica mitochondrial partial genome	465	20	1.10E-30	93.50%
1116	Ac_clus101150_Contig1	acanthoxyla prasina isolate cytochrome oxidase subunit i and cytochrome oxidase subunit ii partial cds mitochondrial	671	20	0.00E+00	99.00%
1120	Ac_clus101156_Contig1	sycon ciliatum mrna for 60s ribosomal protein l44 (rpl44 gene)	446	2	7.40E-39	71.50%
1122	Ac_clus101157_Contig1	nasonia vitripennis muscle lim protein transcript variant mrna	548	2	8.60E-128	77.50%
1123	Ac_clus101159_Contig1	bacillus atticus partial genome	411	20	8.30E-38	81.75%
1127	Ac_clus101160_Contig1	drosophila melanogaster myosin heavy chain transcript variant mrna	596	20	0.00E+00	80.25%
1140	Ac_clus101165_Contig1	papilio xuthus mrna for muscle myosin heavy complete clone: fsg31	220	1	1.10E-32	75.00%
1171	Ac_clus101209_Contig1	culex quinquefasciatus vacuolar atp synthase subunit mrna	454	3	1.50E-98	77.00%
1175	Ac_clus101214_Contig1	bombyx mori genomic chromosome bac clone: complete sequence	498	18	4.90E-67	87.61%
1177	Ac_clus101215_Contig1	tribolium castaneum agap001884-pa mrna	265	20	5.10E-57	79.75%
1201	Ac_clus101241_Contig1	scylla paramamosain g protein beta 1 complete cds	390	5	4.30E-79	76.20%
1204	Ac_clus101243_Contig1	megachile rotundata guanine nucleotide-binding protein g subunit alpha- transcript variant 2 mrna	469	5	5.30E-98	80.00%
1213	Ac_clus101252_Contig1	oreochromis niloticus adenosylhomocysteinase b-like mrna	607	4	4.40E-94	71.00%
1257	Ac_clus101299_Contig1	cardisoma armatum mrna for mitochondrial manganese superoxide dismutase (m gene)	733	11	1.20E-70	69.64%
1279	Ac_clus101324_Contig1	drosophila ananassae gf21115 (dana\gf21115) mrna	443	4	6.90E-90	75.00%
1292	Ac_clus101336_Contig1	megachile rotundata tar dna-binding protein 43-like mrna	793	6	4.50E-108	71.67%
1302	Ac_clus101344_Contig1	echinops telfairi liver mrna	349	20	5.30E-52	76.55%
1306	Ac_clus101326_Contig1	ornithorhynchus anatinus ubiquitin-conjugating enzyme e2 j2-like mrna	212	1	5.80E-36	80.00%
1311	Ac_clus101352_Contig1	apis florea ubiquitin-conjugating enzyme e2 variant 2-like mrna	390	1	1.50E-59	75.00%
1318	Ac_clus101359_Contig1	ciona intestinalis gamma-aminobutyric acid receptor-associated mrna	227	3	2.80E-21	82.33%
1321	Ac_clus101362_Contig1	loxosceles arizonica clone 1 sphingomyelinase d - like protein exon 1 and partial cds	382	2	2.80E-62	86.00%
1331	Ac_clus101372_Contig1	sorex araneus tubulin alpha-3 chain-like mrna	290	20	4.00E-122	92.25%
1334	Ac_clus101375_Contig1	Clavispora lusitaniae ATCC 42720 hypothetical protein, mRNA	757	1	2.40E-54	69.00%
1359	Ac_clus101401_Contig1	monosiga brevicollis mx1 alpha tubulin monbrdraft_17634 complete cds	261	20	2.70E-98	87.60%
1373	Ac_clus101414_Contig1	gryllus bimaculatus gbcontig29117	251	1	9.80E-47	77.00%
1383	Ac_clus101425_Contig1	oryzias latipes adenosylhomocysteinase b-like mrna	350	20	1.60E-77	76.10%
1385	Ac_clus101427_Contig1	meleagris gallopavo phosphoglycerate kinase-like mrna	371	4	1.20E-41	74.75%
1394	Ac_clus101436_Contig1	anopheles gambiae pest agap010750-pa complete cds	221	2	7.90E-22	85.00%
1399	Ac_clus101441_Contig1	apis florea 26s proteasome non-atpase regulatory subunit 6-like mrna	369	4	1.00E-67	75.50%
1406	Ac_clus101448_Contig1	drosophila melanogaster eukaryotic initiation factor 4a (eif-4a) transcript variant mrna	479	20	5.80E-85	73.95%
1411	Ac_clus101453_Contig1	marsupenaeus japonicus mrna for glycogen complete cds	775	20	6.90E-112	68.95%
1425	Ac_clus101467_Contig1	cavia porcellus member ras oncogene family mrna	471	1	1.70E-15	78.00%
1426	Ac_clus101468_Contig1	culex quinquefasciatus gtp-binding protein mrna	378	2	2.50E-69	80.50%
1453	Ac_clus101500_Contig1	loxosceles arizonica clone 1 sphingomyelinase d - like protein exon 1 and partial cds	264	1	4.40E-45	91.00%
1456	Ac_clus101503_Contig1	helicoverpa armigera vacuolar v-type h(+)-atpase b subunit complete cds	626	20	2.00E-174	79.40%
1459	Ac_clus101506_Contig1	nematostella vectensis protein partial mrna	491	20	1.80E-72	82.10%

1471	Ac_clus101519_Contig1	tribolium castaneum agap010429-pa mrna	289	2	4.90E-64	75.50%
1511	Ac_clus101562_Contig1	drosophila melanogaster ryanodine receptor 44f (rya-r44f) transcript variant mrna	638	20	0.00E+00	84.15%
1520	Ac_clus101569_Contig1	single read from an extremity of a full-length cdna clone made from anopheles gambiae total adult 5-prime end of clone fk0aab3bf06 of strain 6-9 of anopheles gambiae (african malaria mosquito)	359	20	2.10E-76	79.95%
1525	Ac_clus101576_Contig1	oreochromis niloticus utp--glucose-1-phosphate uridylyltransferase-like mrna	449	5	4.10E-23	71.20%
1546	Ac_clus101597_Contig1	takifugu rubripes v-type proton atpase subunit brain isoform-like mrna	807	1	4.60E-32	78.00%
1552	Ac_clus101601_Contig1	barentsia elongata ribosomal protein s13 complete cds	526	1	1.40E-80	74.00%
1565	Ac_clus101616_Contig1	plutella xylostella mrna for ribosomal protein complete cds	519	1	4.50E-93	78.00%
1570	Ac_clus101622_Contig1	drosophila subobscura troponin c 73f partial cds	344	1	2.10E-63	78.00%
1601	Ac_clus101654_Contig1	meladema coriacea mrna for ribosomal protein l7ae (rpl7ae gene)	421	1	1.30E-54	73.00%
1617	Ac_clus101671_Contig1	tetraodon nigroviridis full-length cdna	293	20	2.60E-99	87.30%
1620	Ac_clus101676_Contig1	orestes mouhotii mitochondrial partial genome	365	20	3.10E-36	88.85%
1624	Ac_clus101677_Contig1	monochamus alternatus adenine nucleotide translocase complete cds	824	20	1.80E-157	72.35%
1632	Ac_clus101687_Contig1	echinops telfairi cysteine and glycine-rich protein 1 mrna	254	1	1.90E-11	91.00%
1638	Ac_clus101693_Contig1	ictalurus punctatus ribosomal protein l41 mrna	365	3	3.30E-23	92.00%
1641	Ac_clus101696_Contig1	nasonia vitripennis tropomyosin 1 transcript variant mrna	550	20	4.20E-138	79.55%
1642	Ac_clus101697_Contig1	sus scrofa clone: expressed in mesenteric lymph node	227	20	3.00E-65	85.00%
1644	Ac_clus101700_Contig1	aedes aegypti tropomyosin invertebrate partial mrna	343	20	1.00E-111	82.90%
1649	Ac_clus101704_Contig1	bacillus rossius partial genome	1184	20	0.00E+00	80.20%
1650	Ac_clus101705_Contig1	heteropteryx dilatata mitochondrial complete genome	829	20	0.00E+00	77.75%
1655	Ac_clus101710_Contig1	gallus gallus ubiquitin-conjugating enzyme e2 b-like transcript variant mrna	276	8	5.30E-57	78.50%
1663	Ac_clus101719_Contig1	rattus norvegicus methylenetetrahydrofolate dehydrogenase (nadp+ dependent) methenyltetrahydrofolate formyltetrahydrofolate mrna (cdna clone mgc:108690 image:7323085) complete cds	276	2	1.20E-14	79.00%
1665	Ac_clus101721_Contig1	aedes aegypti eukaryotic translation initiation factor 3 subunit partial mrna	394	1	4.70E-28	81.00%
1667	Ac_clus101724_Contig1	nasonia vitripennis sodium potassium-transporting atpase subunit alpha-like miscrna	285	12	1.40E-64	79.33%
1732	Ac_clus101802_Contig1	papilio xuthus membrane protein sequence id: px- expressed in epidermis	249	1	3.90E-20	81.00%
1733	Ac_clus101803_Contig1	drosophila erecta gg24920 (dere\gg24920) mrna	233	1	1.20E-13	84.00%
1744	Ac_clus101814_Contig1	parapanteles oconnor04 voucher para29208 alpha-spectrin partial cds	515	7	1.10E-62	78.86%
1778	Ac_clus101854_Contig1	drosophila willistoni gk21976 (dwil\gk21976) mrna	362	2	5.90E-39	73.00%
1779	Ac_clus101855_Contig1	xenopus tropicalis cytochrome c oxidase assembly protein mitochondrial-like transcript variant mrna	327	3	6.40E-38	78.00%
1784	Ac_clus101857_Contig1	nasonia vitripennis dynein light chain a (dlc-a) mrna	330	4	7.90E-56	81.00%
1796	Ac_clus101867_Contig1	sorex araneus dipeptidyl-peptidase 3 transcript variant mrna	242	10	1.50E-31	78.20%
1821	Ac_clus101902_Contig1	anopheles gambiae pest agap002182-pa complete cds	219	1	6.50E-42	78.00%
1823	Ac_clus101905_Contig1	drosophila melanogaster chd64 transcript variant mrna	443	14	1.90E-46	71.29%
1857	Ac_clus101938_Contig1	drosophila mojavensis gi10520 (dmoj\gi10520) mrna	351	1	1.90E-13	65.00%
1862	Ac_clus101944_Contig1	paracyclopina nana heat shock protein 70 complete cds	304	2	4.30E-27	72.00%
1868	Ac_clus101950_Contig1	ixodes scapularis clathrin heavy mrna	230	1	1.00E-39	79.00%
1893	Ac_clus101979_Contig1	megachile rotundata eukaryotic translation initiation factor 4 gamma 2-like mrna	243	7	1.60E-37	75.86%
1895	Ac_clus101982_Contig1	micromonas rcc299 chromosome 16 protein complete cds	254	2	7.70E-42	75.00%
1916	Ac_clus102009_Contig1	gryllus bimaculatus gbcontig30018	288	1	7.80E-49	88.00%
1955	Ac_clus102053_Contig1	aedes aegypti aspartate aminotransferase partial mrna	422	2	4.70E-60	71.00%
1990	Ac_clus102089_Contig1	salpingoeca atcc 50818 fumarate hydratase complete cds	252	2	2.60E-41	75.00%
2027	Ac_clus102134_Contig1	maylandia zebra transforming transcript variant mrna	668	20	1.30E-107	76.95%
2039	Ac_clus102145_Contig1	culex quinquefasciatus superoxide mrna	659	6	1.80E-61	73.83%
2051	Ac_clus102161_Contig1	cnaphalocrocis medinalis ryanodine receptor complete cds	337	4	8.10E-113	83.50%
2059	Ac_clus102171_Contig1	megachile rotundata uncharacterized loc100881888 mrna	449	4	2.80E-101	74.00%
2065	Ac_clus102176_Contig1	valerianella locusta internal transcribed spacer partial sequence ribosomal rna complete sequence and internal transcribed spacer partial sequence	211	20	7.50E-79	98.05%
2068	Ac_clus102180_Contig1	pachycladon cheesemanii complete genome	326	20	6.00E-165	99.60%

2094	Ac_clus102202_Contig1	tribolium castaneum discs large 1 cg1725-pk mrna	314	1	2.60E-74	80.00%
2096	Ac_clus102212_Contig1	takifugu rubripes afg3-like protein 2-like mrna	206	2	1.30E-18	85.00%
2146	Ac_clus102268_Contig1	condylura cristata dead (asp-glu-ala-asp) box helicase 17 transcript variant mrna	335	4	2.00E-44	73.00%
2150	Ac_clus102273_Contig1	setaria italica long chain acyl- synthetase 4-like transcript variant mrna	309	5	3.40E-22	77.80%
2174	Ac_clus102323_Contig1	dendroctonus ponderosae whole genome shotgun sequence	226	9	3.50E-58	80.22%
2181	Ac_clus102329_Contig1	gryllus bimaculatus gbcontig23843	214	1	5.90E-17	96.00%
2192	Ac_clus102331_Contig1	hyphantria cunea clone c-3 phosphoglucose isomerase complete cds	253	20	8.70E-35	73.80%
2195	Ac_clus102346_Contig1	loxodonta africana t-complex protein 1 subunit zeta- transcript variant 3 mrna	315	3	1.90E-25	71.00%
2224	Ac_clus102383_Contig1	drosophila melanogaster rm62 transcript variant mrna	252	20	8.70E-35	75.00%
2229	Ac_clus102388_Contig1	dendroctonus ponderosae whole genome shotgun sequence	497	1	4.60E-23	83.00%
2235	Ac_clus102394_Contig1	anopheles gambiae pest agap003843-pa complete cds	506	12	1.90E-15	72.92%
2238	Ac_clus102400_Contig1	drosophila melanogaster ryanodine receptor 44f (rya-r44f) transcript variant mrna	208	16	1.30E-56	79.50%
2252	Ac_clus102414_Contig1	drosophila pseudoobscura pseudoobscura ga10593 (dpse\ga10593) transcript variant mrna	475	8	1.30E-99	78.00%
2268	Ac_clus102430_Contig1	plutella xylostella ryanodine receptor complete cds	252	7	1.80E-62	80.57%
2270	Ac_clus102432_Contig1	sus scrofa nadh dehydrogenase	226	20	2.30E-79	87.00%
2275	Ac_clus102437_Contig1	drosophila ananassae gf21831 (dana\gf21831) mrna	415	17	1.30E-98	77.94%
2278	Ac_clus102442_Contig1	drosophila melanogaster vermiform transcript variant mrna	310	20	6.00E-95	84.10%
2285	Ac_clus102448_Contig1	gryllus bimaculatus gbcontig26019	206	1	1.40E-62	90.00%
2308	Ac_clus102471_Contig1	ovis aries iron-sulfur cluster assembly 1 mitochondrial-like mrna	627	14	4.00E-44	73.36%
2310	Ac_clus102474_Contig1	gryllus bimaculatus accessory gland protein (ag-0220p-gb) partial cds	250	5	1.10E-20	81.20%
2324	Ac_clus102490_Contig1	megachile rotundata ryanodine receptor 44f-like mrna	497	1	1.10E-81	74.00%
2330	Ac_clus102447_Contig1	arabidopsis thaliana psi type iii chlorophyll a b-binding protein complete cds	224	18	1.40E-94	92.89%
2342	Ac_clus102511_Contig1	callithrix jacchus peptidylprolyl isomerase c (cyclophilin c) mrna	430	2	2.30E-51	73.50%
2348	Ac_clus102519_Contig1	salmo salar clone ssal-evf-567-335 ring-box protein 1 complete cds	216	20	1.00E-64	84.60%
2359	Ac_clus102527_Contig1	drosophila melanogaster cg2010 transcript variant mrna	211	16	5.10E-62	84.31%
2360	Ac_clus102529_Contig1	drosophila pseudoobscura pseudoobscura ga25309 (dpse\ga25309) mrna	448	20	1.00E-68	73.15%
2392	Ac_clus102563_Contig1	pachycladon cheesemanii complete genome	344	20	1.10E-174	100.00%
2394	Ac_clus102565_Contig1	tetraodon nigroviridis full-length cdna	415	9	4.90E-47	80.78%
2438	Ac_clus102613_Contig1	ciona intestinalis lon protease 2-like mrna	236	2	7.50E-29	77.50%
2447	Ac_clus102623_Contig1	ematurga atomaria voucher ns43 ribosomal protein s5 partial cds	432	9	3.50E-68	78.00%
2450	Ac_clus102626_Contig1	bombyx mori clone: fcal17l05	282	2	1.10E-40	75.00%
2462	Ac_clus102640_Contig1	hypomecis punctinalis voucher ns18 ribosomal protein s5 partial cds	345	18	1.20E-91	78.17%
2480	Ac_clus102661_Contig1	manduca sexta ribosomal protein s30 complete cds	243	1	8.30E-35	78.00%
2500	Ac_clus102682_Contig1	plutella xylostella 40 protein complete cds	215	20	7.20E-35	85.50%
2507	Ac_clus102691_Contig1	crypsiphona ocultaria isolate cytochrome oxidase subunit 1 partial cds mitochondrial	789	20	0.00E+00	82.45%
2513	Ac_clus102697_Contig1	papaver somniferum ubiquitin complete cds	298	20	8.60E-93	84.90%
2522	Ac_clus102706_Contig1	gryllus bimaculatus gbcontig27912	252	2	4.80E-76	83.50%
2523	Ac_clus102651_Contig1	brugia malayi polyubiquitin precursor partial mrna	274	4	6.40E-37	79.75%
2525	Ac_clus102708_Contig1	gryllus bimaculatus gbcontig28025	409	16	1.50E-148	79.94%
