# Supplementary material for: Sticky Genomes: Using NGS Evidence to Test Hybrid Speciation Hypotheses
Source: PLoS One. 2016 May 17;11(5):e0154911. doi: 10.1371/journal.pone.0154911 (PMC4871368; doi:10.1371/journal.pone.0154911)
Supplement: S4 Table — (PDF) [file pone.0154911.s007.pdf]

No.	Seq. name	Seq. decription	length	hits	min. eValue	sim. Mean
1	Cl_clus100005_Contig1	---NA---	3307	0	-	-
2	Cl_clus100003_Contig1	---NA---	4010	0	-	-
3	Cl_clus100006_Contig1	---NA---	4638	0	-	-
4	Cl_clus100007_Contig1	ochotona princeps tyrosine 3-monooxygenase tryptophan 5-monooxygenase activation eta polypeptide mrna	3611	3	6.40E-76	79.67%
5	Cl_clus100008_Contig1	megachile rotundata twitchin-like mrna	5264	2	0.00E+00	69.00%
6	Cl_clus100011_Contig1	---NA---	2779	0	-	-
7	Cl_clus100010_Contig1	---NA---	3909	0	-	-
8	Cl_clus100012_Contig1	bos taurus isocitrate dehydrogenase 3 (nad+) beta transcript variant mrna	3306	14	1.10E-91	72.86%
9	Cl_clus100013_Contig2	---NA---	5697	0	-	-
10	Cl_clus100009_Contig1	---NA---	4559	0	-	-
11	Cl_clus100014_Contig1	---NA---	2473	0	-	-
12	Cl_clus100015_Contig1	---NA---	4223	0	-	-
13	Cl_clus100016_Contig1	---NA---	3147	0	-	-
14	Cl_clus100017_Contig1	---NA---	3448	0	-	-
15	Cl_clus100018_Contig3	---NA---	3232	0	-	-
16	Cl_clus100019_Contig1	---NA---	2943	0	-	-
17	Cl_clus100020_Contig1	megachile rotundata uncharacterized loc100883123 mrna	2842	5	1.50E-44	80.00%
18	Cl_clus100021_Contig1	gryllus bimaculatus gbcontig30509	2502	1	3.20E-122	72.00%
19	Cl_clus100022_Contig1	---NA---	3375	0	-	-
20	Cl_clus100024_Contig1	drosophila pseudoobscura pseudoobscura ga21679 (dpse\ga21679) transcript variant mrna	2011	9	0.00E+00	74.78%
21	Cl_clus100026_Contig1	---NA---	3127	0	-	-
22	Cl_clus100027_Contig1	---NA---	2772	0	-	-
23	Cl_clus100025_Contig1	---NA---	2004	0	-	-
24	Cl_clus100023_Contig1	---NA---	3005	0	-	-
25	Cl_clus100028_Contig1	acontia aprica voucher aaca enolase protein partial cds	1556	20	0.00E+00	73.85%
26	Cl_clus100029_Contig1	nomascus leucogenys tubulin alpha-1c chain-like mrna	1428	20	0.00E+00	79.95%
27	Cl_clus100030_Contig1	---NA---	4026	0	-	-
28	Cl_clus100031_Contig1	cavia porcellus oxoglutarate dehydrogenase-like transcript variant mrna	3560	16	0.00E+00	71.38%
29	Cl_clus100032_Contig1	---NA---	2627	0	-	-
30	Cl_clus100034_Contig1	---NA---	3066	0	-	-
31	Cl_clus100036_Contig1	---NA---	2568	0	-	-
32	Cl_clus100037_Contig1	---NA---	3128	0	-	-
33	Cl_clus100033_Contig1	---NA---	1958	0	-	-
34	Cl_clus100039_Contig1	salmo salar mrna for glutamate dehydrogenase (gene)	1844	20	0.00E+00	71.25%
35	Cl_clus100040_Contig1	---NA---	1684	0	-	-
36	Cl_clus100041_Contig1	---NA---	2342	0	-	-
37	Cl_clus100038_Contig1	---NA---	2362	0	-	-
38	Cl_clus100042_Contig1	---NA---	1843	0	-	-
39	Cl_clus100035_Contig1	---NA---	2804	0	-	-
40	Cl_clus100043_Contig1	aedes aegypti mitochondrial partial mrna	2923	15	0.00E+00	71.13%
41	Cl_clus100044_Contig1	---NA---	2034	0	-	-
42	Cl_clus100045_Contig1	---NA---	1219	0	-	-
43	Cl_clus100046_Contig1	drosophila melanogaster paramyosin transcript variant mrna	1453	20	0.00E+00	76.75%
44	Cl_clus100047_Contig1	---NA---	2053	0	-	-
45	Cl_clus100048_Contig1	---NA---	2433	0	-	-
46	Cl_clus100049_Contig1	schistocerca gregaria atp-synthase subunit beta complete cds	1988	20	0.00E+00	80.00%
47	Cl_clus100051_Contig1	---NA---	2065	0	-	-
48	Cl_clus100052_Contig1	---NA---	2660	0	-	-
49	Cl_clus100053_Contig1	---NA---	3024	0	-	-
50	Cl_clus100050_Contig1	---NA---	1238	0	-	-
51	Cl_clus100054_Contig1	Dendroctonus ponderosae clone DPO0418_C01 unknown mRNA	1931	1	2.60E-71	71.00%
52	Cl_clus100055_Contig1	gryllus bimaculatus gbcontig22796	2684	1	0.00E+00	79.00%
53	Cl_clus100056_Contig1	---NA---	2057	0	-	-
54	Cl_clus100057_Contig1	---NA---	1861	0	-	-
55	Cl_clus100058_Contig1	---NA---	2169	0	-	-

56	Cl_clus100059_Contig1	---NA---	1382	0 -	-	
57	Cl_clus100060_Contig1	agathemera crassa 18s ribosomal rna partial sequence	1818	20	0.00E+00	98.75%
58	Cl_clus100061_Contig1	helicoverpa armigera heat shock protein 90 complete cds	2096	20	0.00E+00	76.60%
59	Cl_clus100062_Contig1	ostrinia furnacalis strain acb- heat shock cognate 70 kda protein complete cds	2291	20	0.00E+00	78.35%
60	Cl_clus100063_Contig1	---NA---	1592	0 -	-	
61	Cl_clus100065_Contig1	---NA---	1098	0 -	-	
62	Cl_clus100066_Contig1	---NA---	2247	0 -	-	
63	Cl_clus100067_Contig1	aedes aegypti clone ae-10233 pa26-t3 nuclear protein complete cds	2136	3	2.70E-65	73.00%
64	Cl_clus100068_Contig1	---NA---	1779	0 -	-	
65	Cl_clus100064_Contig1	---NA---	1274	0 -	-	
66	Cl_clus100069_Contig1	aedes aegypti eukaryotic translation elongation factor partial mrna	2186	20	0.00E+00	76.90%
67	Cl_clus100070_Contig1	tribolium castaneum darkener of apricot cg33553-pg mrna	1636	2	2.90E-171	72.00%
68	Cl_clus100071_Contig1	---NA---	1787	0 -	-	
69	Cl_clus100072_Contig1	---NA---	1472	0 -	-	
70	Cl_clus100073_Contig2	---NA---	1704	0 -	-	
71	Cl_clus100074_Contig2	---NA---	2132	0 -	-	
72	Cl_clus100075_Contig1	---NA---	3215	0 -	-	
73	Cl_clus100076_Contig1	---NA---	2142	0 -	-	
74	Cl_clus100077_Contig1	---NA---	730	0 -	-	
75	Cl_clus100078_Contig1	---NA---	2341	0 -	-	
76	Cl_clus100079_Contig1	---NA---	1686	0 -	-	
77	Cl_clus100080_Contig1	drosophila melanogaster alpha actinin transcript variant mrna	2956	20	0.00E+00	79.35%
78	Cl_clus100081_Contig1	---NA---	1645	0 -	-	
79	Cl_clus100082_Contig1	---NA---	1105	0 -	-	
80	Cl_clus100083_Contig1	drosophila persimilis gl12270 (dper\gl12270) mrna	2064	1	3.90E-25	77.00%
81	Cl_clus100084_Contig1	---NA---	3487	0 -	-	
82	Cl_clus100086_Contig1	---NA---	1191	0 -	-	
83	Cl_clus100085_Contig1	---NA---	1566	0 -	-	
84	Cl_clus100087_Contig1	---NA---	2039	0 -	-	
85	Cl_clus100088_Contig1	---NA---	2337	0 -	-	
86	Cl_clus100089_Contig1	---NA---	1474	0 -	-	
87	Cl_clus100090_Contig1	tribolium castaneum agap007135-pa mrna	1815	6	0.00E+00	78.50%
88	Cl_clus100091_Contig1	---NA---	1593	0 -	-	
89	Cl_clus100092_Contig1	aedes aegypti troponin c partial mrna	848	16	7.60E-112	75.81%
90	Cl_clus100093_Contig2	bacillus rossius 28s ribosomal rna partial sequence	2358	20	0.00E+00	92.55%
91	Cl_clus100094_Contig1	drosophila sechellia gm12981 (dsec\gm12981) mrna	1441	3	8.30E-51	70.00%
92	Cl_clus100095_Contig1	---NA---	1061	0 -	-	
93	Cl_clus100096_Contig1	---NA---	1726	0 -	-	
94	Cl_clus100097_Contig1	---NA---	1224	0 -	-	
95	Cl_clus100098_Contig1	---NA---	1423	0 -	-	
96	Cl_clus100099_Contig1	---NA---	1363	0 -	-	
97	Cl_clus100100_Contig1	---NA---	1279	0 -	-	
98	Cl_clus100101_Contig1	---NA---	857	0 -	-	
99	Cl_clus100102_Contig1	---NA---	775	0 -	-	
100	Cl_clus100103_Contig1	---NA---	1427	0 -	-	
101	Cl_clus100104_Contig1	---NA---	848	0 -	-	
102	Cl_clus100105_Contig1	pseudoclitarchus sentus isolate pse-231 28s ribosomal rna partial sequence	1146	20	0.00E+00	98.60%
103	Cl_clus100106_Contig1	---NA---	1538	0 -	-	
104	Cl_clus100107_Contig1	culex quinquefasciatus mrna	1578	20	6.90E-141	82.60%
105	Cl_clus100108_Contig1	---NA---	2021	0 -	-	
106	Cl_clus100109_Contig1	rattus norvegicus high mobility group protein b1-like mrna	1450	20	3.50E-68	70.25%
107	Cl_clus100111_Contig1	---NA---	1580	0 -	-	
108	Cl_clus100112_Contig1	---NA---	2316	0 -	-	
109	Cl_clus100113_Contig1	drosophila melanogaster calcium atpase at 60a (ca-p60a) transcript variant mrna	3178	20	0.00E+00	77.80%
110	Cl_clus100110_Contig1	clitarchus hookeri isolate -2 28s ribosomal rna partial sequence	1068	20	0.00E+00	97.40%
111	Cl_clus100114_Contig1	drosophila melanogaster elongation factor 1alpha48d transcript variant mrna	1906	20	0.00E+00	81.70%

112	Cl_clus100115_Contig1	---NA---	1343	0	-	-
113	Cl_clus100116_Contig1	---NA---	1534	0	-	-
114	Cl_clus100117_Contig1	---NA---	835	0	-	-
115	Cl_clus100118_Contig1	sorex araneus protein disulfide isomerase family member 3 transcript variant mrna	1270	3	4.00E-35	70.67%
116	Cl_clus100119_Contig1	---NA---	965	0	-	-
117	Cl_clus100120_Contig1	---NA---	1610	0	-	-
118	Cl_clus100121_Contig1	---NA---	1617	0	-	-
119	Cl_clus100122_Contig1	biston betularia ribosomal protein l4 partial cds	1424	20	5.90E-135	69.15%
120	Cl_clus100123_Contig1	---NA---	1017	0	-	-
121	Cl_clus100124_Contig1	---NA---	1074	0	-	-
122	Cl_clus100125_Contig1	---NA---	780	0	-	-
123	Cl_clus100126_Contig1	oryza sativa japonica group cdna clone:006-205- full insert sequence	1498	20	6.60E-141	73.00%
124	Cl_clus100127_Contig1	---NA---	637	0	-	-
125	Cl_clus100128_Contig1	---NA---	875	0	-	-
126	Cl_clus100129_Contig1	aedes aegypti troponin c partial mrna	1230	8	5.80E-71	71.63%
127	Cl_clus100130_Contig1	---NA---	952	0	-	-
128	Cl_clus100131_Contig1	---NA---	1977	0	-	-
129	Cl_clus100132_Contig1	---NA---	1667	0	-	-
130	Cl_clus100133_Contig1	---NA---	1326	0	-	-
131	Cl_clus100134_Contig1	---NA---	1146	0	-	-
132	Cl_clus100136_Contig1	---NA---	1645	0	-	-
133	Cl_clus100135_Contig1	tetraodon nigroviridis full-length cdna	1800	20	0.00E+00	80.65%
134	Cl_clus100137_Contig1	---NA---	1155	0	-	-
135	Cl_clus100138_Contig1	---NA---	1205	0	-	-
136	Cl_clus100139_Contig1	---NA---	1745	0	-	-
137	Cl_clus100140_Contig1	drosophila mojavensis gi12770 (dmoj\gi12770) mrna	461	20	3.70E-125	78.95%
138	Cl_clus100141_Contig1	chilo suppressalis complete genome	1132	20	0.00E+00	75.10%
139	Cl_clus100142_Contig1	---NA---	1331	0	-	-

140	Cl_clus100143_Contig1	trichophyton rubrum cbs 118892 atp-dependent rna helicase eif4a complete cds	1401	18	0.00E+00	71.83%
141	Cl_clus100145_Contig1	---NA---	939	0	-	-
142	Cl_clus100144_Contig1	---NA---	1708	0	-	-
143	Cl_clus100146_Contig1	---NA---	1717	0	-	-
144	Cl_clus100147_Contig1	---NA---	1762	0	-	-
145	Cl_clus100148_Contig1	papilio xuthus mrna for beta- complete sequence id: px-0214	1627	20	0.00E+00	81.50%
146	Cl_clus100149_Contig1	tentyria rotundata partial mp20 gene for muscular protein specimen voucher bmnh exons 1-2	994	20	4.00E-116	70.65%
147	Cl_clus100151_Contig1	---NA---	2347	0	-	-
148	Cl_clus100150_Contig1	gallus gallus tripartite motif-containing 3 transcript variant mrna	1861	5	1.00E-139	87.40%
149	Cl_clus100152_Contig1	---NA---	2749	0	-	-
150	Cl_clus100153_Contig1	drosophila melanogaster ferritin 1 heavy chain homologue transcript variant mrna	1122	20	1.20E-53	76.70%
151	Cl_clus100154_Contig1	---NA---	691	0	-	-
152	Cl_clus100156_Contig1	---NA---	1436	0	-	-
153	Cl_clus100155_Contig1	---NA---	1493	0	-	-
154	Cl_clus100157_Contig1	---NA---	1911	0	-	-
155	Cl_clus100159_Contig1	---NA---	950	0	-	-
156	Cl_clus100160_Contig1	---NA---	1299	0	-	-
157	Cl_clus100158_Contig1	---NA---	857	0	-	-
158	Cl_clus100161_Contig1	---NA---	884	0	-	-
159	Cl_clus100162_Contig1	---NA---	1359	0	-	-
160	Cl_clus100163_Contig1	---NA---	872	0	-	-
161	Cl_clus100164_Contig1	---NA---	3612	0	-	-
162	Cl_clus100166_Contig1	---NA---	872	0	-	-
163	Cl_clus100165_Contig1	---NA---	1092	0	-	-
164	Cl_clus100167_Contig1	---NA---	1052	0	-	-
165	Cl_clus100168_Contig1	acyrthosiphon pisum clone: full-insert cdna sequence based on ests (5 - est: 3 -est aci2aaf1yd14bbm1)	1479	14	5.30E-142	70.07%

166	Cl_clus100169_Contig1	---NA---	1228	0	-	-
167	Cl_clus100170_Contig1	---NA---	956	0	-	-
168	Cl_clus100171_Contig1	gryllus bimaculatus gbcontig28300	1070	4	1.00E-79	78.25%
169	Cl_clus100172_Contig1	locusta migratoria gapdh complete cds	1398	20	0.00E+00	75.60%
170	Cl_clus100181_Contig1	---NA---	739	0	-	-
171	Cl_clus100185_Contig1	---NA---	1194	0	-	-
172	Cl_clus100186_Contig1	---NA---	1884	0	-	-
173	Cl_clus100182_Contig1	---NA---	844	0	-	-
174	Cl_clus100183_Contig1	---NA---	1258	0	-	-
175	Cl_clus100187_Contig2	---NA---	1678	0	-	-
176	Cl_clus100189_Contig1	---NA---	925	0	-	-
177	Cl_clus100190_Contig1	cricetulus griseus protein transport protein sec61 subunit alpha isoform 1-like mrna	1084	3	5.80E-26	83.67%
178	Cl_clus100191_Contig1	---NA---	1412	0	-	-
179	Cl_clus100184_Contig1	gryllus bimaculatus gbcontig29003	874	1	1.30E-45	73.00%
180	Cl_clus100192_Contig1	---NA---	714	0	-	-
181	Cl_clus100194_Contig1	---NA---	1132	0	-	-
182	Cl_clus100195_Contig1	---NA---	1547	0	-	-
183	Cl_clus100196_Contig1	---NA---	867	0	-	-
184	Cl_clus100197_Contig1	---NA---	1141	0	-	-
185	Cl_clus100198_Contig1	---NA---	1187	0	-	-
186	Cl_clus100199_Contig1	---NA---	804	0	-	-
187	Cl_clus100200_Contig1	---NA---	973	0	-	-
188	Cl_clus100201_Contig1	---NA---	1248	0	-	-
189	Cl_clus100202_Contig1	monodelphis domestica glycogen muscle form-like mrna	1413	3	0.00E+00	71.33%
190	Cl_clus100203_Contig1	---NA---	897	0	-	-
191	Cl_clus100204_Contig1	---NA---	1250	0	-	-
192	Cl_clus100205_Contig1	---NA---	760	0	-	-
193	Cl_clus100206_Contig1	---NA---	1516	0	-	-
194	Cl_clus100207_Contig1	---NA---	961	0	-	-

195	Cl_clus100209_Contig1	---NA---	997	0	-	-
196	Cl_clus100210_Contig1	---NA---	1182	0	-	-
197	Cl_clus100211_Contig1	---NA---	788	0	-	-
198	Cl_clus100212_Contig1	gryllus bimaculatus gbcontig06695	849	1	4.80E-13	81.00%
199	Cl_clus100213_Contig1	---NA---	839	0	-	-
200	Cl_clus100214_Contig1	---NA---	717	0	-	-
201	Cl_clus100215_Contig1	---NA---	467	0	-	-
202	Cl_clus100216_Contig1	---NA---	679	0	-	-
203	Cl_clus100218_Contig1	---NA---	787	0	-	-
204	Cl_clus100188_Contig1	---NA---	1289	0	-	-
205	Cl_clus100217_Contig1	---NA---	1345	0	-	-
206	Cl_clus100193_Contig1	---NA---	760	0	-	-
207	Cl_clus100219_Contig1	sycon ciliatum mrna for 60s ribosomal protein l44 (rpl44 gene)	448	2	9.10E-38	72.00%
208	Cl_clus100220_Contig1	---NA---	513	0	-	-
209	Cl_clus100208_Contig1	---NA---	428	0	-	-
210	Cl_clus100221_Contig1	---NA---	426	0	-	-
211	Cl_clus100222_Contig1	aedes albopictus clone al_249 translationally controlled tumor protein complete cds	830	6	3.00E-47	67.33%
212	Cl_clus100223_Contig1	---NA---	1283	0	-	-
213	Cl_clus100224_Contig1	---NA---	1119	0	-	-
214	Cl_clus100225_Contig1	---NA---	1601	0	-	-
215	Cl_clus100227_Contig1	---NA---	1252	0	-	-
216	Cl_clus100228_Contig1	---NA---	1340	0	-	-
217	Cl_clus100229_Contig1	drosophila pseudoobscura pseudoobscura ga25309 (dpse\ga25309) mrna	1438	20	8.80E-114	68.50%
218	Cl_clus100230_Contig1	leptospira biflexa serovar patoc strain patoc 1 chromosome complete sequence	1193	7	7.70E-177	71.57%
219	Cl_clus100231_Contig1	---NA---	1460	0	-	-
220	Cl_clus100233_Contig1	---NA---	1067	0	-	-
221	Cl_clus100232_Contig1	---NA---	1089	0	-	-

222	Cl_clus100234_Contig1	single read from an extremity of a full-length cdna clone made from anopheles gambiae total adult 5-prime end of clone fk0aaa6ag11 of strain 6-9 of anopheles gambiae (african malaria mosquito)	1568	20	4.40E-80	80.60%
223	Cl_clus100235_Contig1	---NA---	961	0 -	-	
224	Cl_clus100236_Contig1	---NA---	1463	0 -	-	
225	Cl_clus100237_Contig1	---NA---	1214	0 -	-	
226	Cl_clus100238_Contig1	papilio polytes mrna for ribosomal protein complete sequence id: pp-0493	1261	20	0.00E+00	72.65%
227	Cl_clus100239_Contig1	dasypus novemcinctus liver transcript variant mrna	927	7	1.20E-103	75.71%
228	Cl_clus100240_Contig1	---NA---	845	0 -	-	
229	Cl_clus100241_Contig1	bombus impatiens protein translation factor sui1 homolog mrna	1088	20	2.60E-106	81.45%
230	Cl_clus100243_Contig1	mus musculus ribosomal protein pseudogene (rps23-ps) on chromosome 3	559	20	1.10E-88	76.05%
231	Cl_clus100245_Contig2	---NA---	727	0 -	-	
232	Cl_clus100242_Contig1	drosophila grimshawi gh25015 (dgri\gh25015) mrna	1562	2	2.30E-39	69.00%
233	Cl_clus100244_Contig1	---NA---	1237	0 -	-	
234	Cl_clus100246_Contig1	---NA---	1833	0 -	-	
235	Cl_clus100247_Contig1	---NA---	1363	0 -	-	
236	Cl_clus100248_Contig1	---NA---	823	0 -	-	
237	Cl_clus100249_Contig1	papilio bianor complete genome	1503	20	1.70E-167	78.70%
238	Cl_clus100250_Contig1	nasonia vitripennis muscle m-line assembly protein unc-89-like mrna	967	2	1.50E-102	71.00%
239	Cl_clus100252_Contig1	---NA---	1302	0 -	-	
240	Cl_clus100251_Contig1	---NA---	1077	0 -	-	
241	Cl_clus100253_Contig1	drosophila melanogaster ribosomal protein s2 transcript variant mrna	904	20	0.00E+00	78.65%
242	Cl_clus100254_Contig1	coptotermes formosanus clone cfsni164 heavy subunit complete cds	1005	2	3.60E-47	66.00%
243	Cl_clus100255_Contig1	---NA---	2142	0 -	-	
244	Cl_clus100256_Contig1	---NA---	1127	0 -	-	
245	Cl_clus100257_Contig1	diaphorina citri clone whdc1630 atp synthase gamma subunit partial cds	821	1	5.60E-31	77.00%
246	Cl_clus100258_Contig1	---NA---	645	0 -	-	
247	Cl_clus100259_Contig2	reticulitermes flavipes beta-actin complete cds	1576	20	0.00E+00	84.95%
248	Cl_clus100260_Contig1	---NA---	952	0 -	-	
249	Cl_clus100261_Contig1	drosophila melanogaster myosin heavy chain transcript variant mrna	1933	20	0.00E+00	85.15%
250	Cl_clus100262_Contig1	---NA---	1602	0 -	-	
251	Cl_clus100263_Contig1	---NA---	898	0 -	-	
252	Cl_clus100264_Contig1	---NA---	1326	0 -	-	
253	Cl_clus100265_Contig1	---NA---	1066	0 -	-	
254	Cl_clus100266_Contig1	---NA---	1179	0 -	-	
255	Cl_clus100267_Contig1	---NA---	934	0 -	-	
256	Cl_clus100268_Contig1	---NA---	1226	0 -	-	
257	Cl_clus100269_Contig1	---NA---	852	0 -	-	
258	Cl_clus100270_Contig1	---NA---	919	0 -	-	
259	Cl_clus100271_Contig1	---NA---	779	0 -	-	
260	Cl_clus100272_Contig1	---NA---	980	0 -	-	
261	Cl_clus100273_Contig1	---NA---	1179	0 -	-	
262	Cl_clus100274_Contig1	---NA---	625	0 -	-	
263	Cl_clus100275_Contig1	---NA---	604	0 -	-	
264	Cl_clus100276_Contig1	---NA---	1319	0 -	-	
265	Cl_clus100277_Contig1	---NA---	1863	0 -	-	
266	Cl_clus100278_Contig1	---NA---	1159	0 -	-	
267	Cl_clus100279_Contig1	maconellicoccus hirsutus clone whmh2520 ribosomal protein l13 complete cds	723	2	4.30E-57	68.50%
268	Cl_clus100280_Contig1	cicer arietinum t-complex protein 1 subunit epsilon-like mrna	1015	1	6.20E-57	68.00%
269	Cl_clus100281_Contig1	---NA---	849	0 -	-	
270	Cl_clus100283_Contig1	anopheles gambiae pest agap002154-pb partial cds	1237	2	1.40E-72	69.00%
271	Cl_clus100284_Contig1	---NA---	1267	0 -	-	
272	Cl_clus100285_Contig1	---NA---	978	0 -	-	
273	Cl_clus100286_Contig1	drosophila sechellia gm26300 (dsec\gm26300) mrna	1272	3	4.00E-35	80.33%
274	Cl_clus100287_Contig1	---NA---	406	0 -	-	
275	Cl_clus100282_Contig1	---NA---	1091	0 -	-	

276	Cl_clus100288_Contig1	---NA---	1075	0	-	-
277	Cl_clus100289_Contig1	---NA---	835	0	-	-
278	Cl_clus100290_Contig1	---NA---	595	0	-	-
279	Cl_clus100291_Contig1	---NA---	572	0	-	-
280	Cl_clus100292_Contig1	ematurga atomaria voucher ns43 ribosomal protein s5 partial cds	768	20	1.60E-151	78.75%
281	Cl_clus100293_Contig1	---NA---	887	0	-	-
282	Cl_clus100294_Contig1	---NA---	836	0	-	-
283	Cl_clus100295_Contig1	---NA---	489	0	-	-
284	Cl_clus100296_Contig1	tetraodon nigroviridis full-length cdna	588	20	1.50E-93	74.05%
285	Cl_clus100300_Contig1	nasonia vitripennis peptidyl-prolyl cis-trans isomerase- transcript variant 2 mrna	1143	20	2.40E-94	74.20%
286	Cl_clus100301_Contig1	---NA---	1048	0	-	-
287	Cl_clus100302_Contig1	callorhinchus milii clone ribosomal protein l10 complete cds	739	20	2.30E-168	76.05%
288	Cl_clus100303_Contig1	---NA---	543	0	-	-
289	Cl_clus100304_Contig1	---NA---	333	0	-	-
290	Cl_clus100305_Contig1	---NA---	412	0	-	-
291	Cl_clus100306_Contig1	---NA---	699	0	-	-
292	Cl_clus100307_Contig1	---NA---	1025	0	-	-
293	Cl_clus100308_Contig1	---NA---	1049	0	-	-
294	Cl_clus100309_Contig1	placozoa h4 eukaryotic translation initiation factor 5a complete cds	991	2	7.30E-56	81.50%
295	Cl_clus100310_Contig1	---NA---	881	0	-	-
296	Cl_clus100311_Contig1	---NA---	1313	0	-	-
297	Cl_clus100312_Contig1	---NA---	905	0	-	-
298	Cl_clus100313_Contig1	---NA---	690	0	-	-
299	Cl_clus100314_Contig1	---NA---	1522	0	-	-
300	Cl_clus100315_Contig1	drosophila melanogaster ryanodine receptor 44f (rya-r44f) transcript variant mrna	925	20	4.60E-134	73.45%
301	Cl_clus100316_Contig1	---NA---	709	0	-	-
302	Cl_clus100317_Contig1	---NA---	894	0	-	-
303	Cl_clus100318_Contig1	---NA---	685	0	-	-
304	Cl_clus100319_Contig1	---NA---	1032	0	-	-
305	Cl_clus100321_Contig1	---NA---	1171	0	-	-
306	Cl_clus100320_Contig1	---NA---	845	0	-	-
307	Cl_clus100322_Contig1	sorex araneus nucleosome assembly protein 1-like 4 mrna	892	2	3.20E-28	72.00%
308	Cl_clus100323_Contig1	---NA---	588	0	-	-
309	Cl_clus100324_Contig1	---NA---	1528	0	-	-
310	Cl_clus100325_Contig1	---NA---	556	0	-	-
311	Cl_clus100326_Contig1	---NA---	994	0	-	-
312	Cl_clus100327_Contig1	---NA---	955	0	-	-
313	Cl_clus100328_Contig1	clitarchus hookeri voucher nzac03005012 cytochrome oxidase subunit i partial cds mitochondrial	982	20	0.00E+00	96.50%
314	Cl_clus100329_Contig1	---NA---	496	0	-	-
315	Cl_clus100330_Contig1	---NA---	448	0	-	-
316	Cl_clus100331_Contig1	---NA---	645	0	-	-
317	Cl_clus100332_Contig1	---NA---	1164	0	-	-
318	Cl_clus100333_Contig1	---NA---	577	0	-	-
319	Cl_clus100334_Contig1	drosophila melanogaster eukaryotic initiation factor 1a (eif-1a) transcript variant mrna	1033	20	2.80E-99	77.00%
320	Cl_clus100335_Contig1	---NA---	955	0	-	-
321	Cl_clus100336_Contig1	---NA---	1225	0	-	-
322	Cl_clus100337_Contig1	---NA---	1266	0	-	-
323	Cl_clus100338_Contig1	chrysomela tremulae ribosomal protein s7 complete cds	663	1	4.50E-107	77.00%
324	Cl_clus100339_Contig1	papilio xuthus mrna for ribosomal protein complete sequence id: px-0053	919	3	1.60E-171	74.33%
325	Cl_clus100340_Contig1	---NA---	1063	0	-	-
326	Cl_clus100341_Contig1	---NA---	1493	0	-	-
327	Cl_clus100342_Contig1	---NA---	1478	0	-	-
328	Cl_clus100343_Contig1	bacillus atticus partial genome	752	20	5.50E-37	87.85%
329	Cl_clus100344_Contig1	gryllus bimaculatus gbcontig00020	1128	20	0.00E+00	76.40%
330	Cl_clus100345_Contig1	---NA---	656	0	-	-

331	Cl_clus100346_Contig1	---NA---	1137	0	-	-
332	Cl_clus100347_Contig1	---NA---	625	0	-	-
333	Cl_clus100349_Contig1	---NA---	1333	0	-	-
334	Cl_clus100350_Contig1	---NA---	1123	0	-	-
335	Cl_clus100351_Contig1	---NA---	1299	0	-	-
336	Cl_clus100348_Contig1	---NA---	1218	0	-	-
337	Cl_clus100352_Contig1	aspergillus oryzae rib40 polyadenylate-binding cytoplasmic and mrna	1473	12	0.00E+00	72.83%
338	Cl_clus100354_Contig1	---NA---	1090	0	-	-
339	Cl_clus100353_Contig1	gryllus bimaculatus gbcontig31212	661	1	1.20E-44	74.00%
340	Cl_clus100355_Contig1	---NA---	1924	0	-	-
341	Cl_clus100356_Contig1	---NA---	684	0	-	-
342	Cl_clus100357_Contig1	---NA---	1652	0	-	-
343	Cl_clus100358_Contig1	tribolium castaneum troponin c mrna	711	4	1.00E-77	77.75%
344	Cl_clus100359_Contig1	aedes aegypti atpase subunit partial mrna	614	20	9.80E-52	86.25%
345	Cl_clus100360_Contig1	---NA---	453	0	-	-
346	Cl_clus100361_Contig1	---NA---	984	0	-	-
347	Cl_clus100362_Contig2	gryllus bimaculatus gbcontig29225	1215	1	5.00E-21	65.00%
348	Cl_clus100363_Contig1	drosophila willistoni gk13316 (dwil\gk13316) mrna	1713	1	1.60E-35	76.00%
349	Cl_clus100364_Contig1	---NA---	1238	0	-	-
350	Cl_clus100365_Contig1	---NA---	2140	0	-	-
351	Cl_clus100366_Contig1	---NA---	1607	0	-	-
352	Cl_clus100367_Contig1	---NA---	1414	0	-	-
353	Cl_clus100368_Contig1	---NA---	481	0	-	-
354	Cl_clus100369_Contig1	---NA---	1371	0	-	-
355	Cl_clus100370_Contig1	---NA---	700	0	-	-
356	Cl_clus100371_Contig1	---NA---	818	0	-	-
357	Cl_clus100372_Contig1	---NA---	756	0	-	-
358	Cl_clus100373_Contig1	---NA---	702	0	-	-
359	Cl_clus100374_Contig1	---NA---	843	0	-	-
360	Cl_clus100375_Contig1	---NA---	1215	0	-	-

361	Cl_clus100376_Contig1	---NA---	959	0	-	-
362	Cl_clus100377_Contig1	---NA---	977	0	-	-
363	Cl_clus100379_Contig1	---NA---	803	0	-	-
364	Cl_clus100378_Contig1	---NA---	732	0	-	-
365	Cl_clus100380_Contig1	---NA---	1322	0	-	-
366	Cl_clus100382_Contig1	---NA---	618	0	-	-
367	Cl_clus100383_Contig1	---NA---	698	0	-	-
368	Cl_clus100384_Contig1	---NA---	796	0	-	-
369	Cl_clus100385_Contig1	---NA---	453	0	-	-
370	Cl_clus100386_Contig1	---NA---	1126	0	-	-
371	Cl_clus100387_Contig1	---NA---	766	0	-	-
372	Cl_clus100388_Contig1	---NA---	639	0	-	-
373	Cl_clus100389_Contig1	---NA---	858	0	-	-
374	Cl_clus100390_Contig1	---NA---	834	0	-	-
375	Cl_clus100391_Contig1	---NA---	645	0	-	-
376	Cl_clus100392_Contig1	---NA---	824	0	-	-
377	Cl_clus100393_Contig1	---NA---	404	0	-	-
378	Cl_clus100394_Contig1	---NA---	838	0	-	-
379	Cl_clus100395_Contig1	---NA---	928	0	-	-
380	Cl_clus100396_Contig1	---NA---	1001	0	-	-
381	Cl_clus100397_Contig1	---NA---	478	0	-	-
382	Cl_clus100398_Contig1	---NA---	752	0	-	-
383	Cl_clus100399_Contig1	---NA---	775	0	-	-
384	Cl_clus100401_Contig1	---NA---	1000	0	-	-
385	Cl_clus100402_Contig1	tribolium castaneum s-adenosylmethionine transcript variant 1 mrna	397	1	9.70E-18	95.00%
386	Cl_clus100403_Contig1	single read from an extremity of a full-length cdna clone made from anopheles gambiae total adult 3-prime end of clone fk0aaa29ca04 of strain 6-9 of anopheles gambiae (african malaria mosquito)	425	1	3.40E-30	79.00%
387	Cl_clus100404_Contig1	---NA---	798	0	-	-
388	Cl_clus100405_Contig1	drosophila melanogaster cg2010 transcript variant mrna	1065	18	0.00E+00	76.83%

389	Cl_clus100406_Contig1	---NA---	647	0 -	-	
390	Cl_clus100407_Contig1	---NA---	569	0 -	-	
391	Cl_clus100408_Contig1	plutella xylostella 40 protein complete cds	657	20	1.90E-67	81.05%
392	Cl_clus100409_Contig1	---NA---	528	0 -	-	
393	Cl_clus100410_Contig1	---NA---	523	0 -	-	
394	Cl_clus100411_Contig1	---NA---	484	0 -	-	
395	Cl_clus100412_Contig1	anolis carolinensis atp synthase subunit mitochondrial-like mrna	552	1	1.30E-11	75.00%
396	Cl_clus100414_Contig1	chrysomela tremulae ribosomal protein l7 complete cds	840	2	3.90E-109	73.00%
397	Cl_clus100415_Contig1	---NA---	428	0 -	-	
398	Cl_clus100416_Contig1	---NA---	2119	0 -	-	
399	Cl_clus100417_Contig1	---NA---	2229	0 -	-	
400	Cl_clus100418_Contig1	---NA---	1149	0 -	-	
401	Cl_clus100419_Contig1	cardisoma armatum mrna for mitochondrial manganese superoxide dismutase (m gene)	855	11	1.30E-83	70.09%
402	Cl_clus100420_Contig3	---NA---	520	0 -	-	
403	Cl_clus100421_Contig1	---NA---	731	0 -	-	
404	Cl_clus100423_Contig1	---NA---	657	0 -	-	
405	Cl_clus100422_Contig1	---NA---	1014	0 -	-	
406	Cl_clus100424_Contig1	---NA---	882	0 -	-	
407	Cl_clus100425_Contig1	---NA---	965	0 -	-	
408	Cl_clus100426_Contig1	---NA---	924	0 -	-	
409	Cl_clus100427_Contig1	---NA---	1093	0 -	-	
410	Cl_clus100428_Contig1	---NA---	796	0 -	-	
411	Cl_clus100429_Contig1	pan troglodytes mrna for glucose-6-phosphate complete clone: -5-5_c06	802	20	1.40E-95	69.70%
412	Cl_clus100430_Contig1	---NA---	889	0 -	-	
413	Cl_clus100431_Contig1	---NA---	779	0 -	-	
414	Cl_clus100432_Contig1	---NA---	860	0 -	-	
415	Cl_clus100433_Contig1	---NA---	900	0 -	-	
416	Cl_clus100434_Contig1	---NA---	808	0 -	-	
417	Cl_clus100435_Contig1	---NA---	583	0 -	-	
418	Cl_clus100436_Contig1	---NA---	656	0 -	-	
419	Cl_clus100437_Contig1	---NA---	477	0 -	-	
420	Cl_clus100439_Contig1	taeniopygia guttata nadh dehydrogenase flavoprotein 51kda mrna	1259	5	4.20E-155	70.40%
421	Cl_clus100440_Contig1	neurospora crassa or74a peptidyl-prolyl cis-trans isomerase b precursor partial mrna	1307	14	6.10E-71	70.86%
422	Cl_clus100442_Contig1	---NA---	978	0 -	-	
423	Cl_clus100443_Contig1	mus musculus crl-1722 l5178y-r riken full-length enriched clone:i730086p06 product:chaperonin subunit 5 full insert sequence	1004	20	1.10E-110	71.55%
424	Cl_clus100444_Contig1	---NA---	328	0 -	-	
425	Cl_clus100446_Contig1	---NA---	442	0 -	-	
426	Cl_clus100447_Contig1	---NA---	550	0 -	-	
427	Cl_clus100438_Contig1	---NA---	449	0 -	-	
428	Cl_clus100448_Contig1	mustela putorius furo nucleoside diphosphate kinase chloroplastic-like transcript variant mrna	745	8	1.90E-55	72.38%
429	Cl_clus100449_Contig1	nasonia vitripennis muscle m-line assembly protein unc-89-like mrna	1007	4	0.00E+00	73.50%
430	Cl_clus100451_Contig1	---NA---	749	0 -	-	
431	Cl_clus100452_Contig1	---NA---	938	0 -	-	
432	Cl_clus100453_Contig1	---NA---	970	0 -	-	
433	Cl_clus100454_Contig1	---NA---	985	0 -	-	
434	Cl_clus100441_Contig1	---NA---	311	0 -	-	
435	Cl_clus100456_Contig1	---NA---	1490	0 -	-	
436	Cl_clus100457_Contig1	---NA---	953	0 -	-	
437	Cl_clus100458_Contig1	phlebotomus papatasi 40s ribosomal protein s8-like protein complete cds	366	1	7.80E-63	75.00%
438	Cl_clus100459_Contig1	---NA---	661	0 -	-	
439	Cl_clus100461_Contig1	---NA---	466	0 -	-	
440	Cl_clus100462_Contig1	---NA---	1629	0 -	-	
441	Cl_clus100463_Contig1	---NA---	396	0 -	-	
442	Cl_clus100464_Contig1	---NA---	764	0 -	-	

443	Cl_clus100465_Contig1	drosophila melanogaster paxillin transcript variant mrna	821	20	5.20E-177	81.20%
444	Cl_clus100466_Contig1	---NA---	671	0 -	-	
445	Cl_clus100467_Contig1	---NA---	597	0 -	-	
446	Cl_clus100468_Contig1	dna for 18s ribosomal rna	859	20	0.00E+00	96.10%
447	Cl_clus100469_Contig1	boechera divaricarpa isolate slw-d-d09 mrna sequence	618	20	3.00E-172	79.85%
448	Cl_clus100470_Contig1	---NA---	674	0 -	-	
449	Cl_clus100471_Contig1	---NA---	814	0 -	-	
450	Cl_clus100472_Contig1	---NA---	900	0 -	-	
451	Cl_clus100473_Contig1	---NA---	1205	0 -	-	
452	Cl_clus100474_Contig1	---NA---	814	0 -	-	
453	Cl_clus100475_Contig1	---NA---	612	0 -	-	
454	Cl_clus100476_Contig1	sipyloidea sipylus mitochondrial partial genome	1213	20	0.00E+00	81.05%
455	Cl_clus100477_Contig1	---NA---	533	0 -	-	
456	Cl_clus100478_Contig1	---NA---	1665	0 -	-	
457	Cl_clus100479_Contig1	---NA---	1103	0 -	-	
458	Cl_clus100445_Contig1	---NA---	818	0 -	-	
459	Cl_clus100460_Contig1	---NA---	371	0 -	-	
460	Cl_clus100450_Contig1	---NA---	510	0 -	-	
461	Cl_clus100455_Contig1	nasonia vitripennis ribosomal protein l39 mrna	268	1	3.90E-33	81.00%
462	Cl_clus100480_Contig1	---NA---	957	0 -	-	
463	Cl_clus100481_Contig1	---NA---	619	0 -	-	
464	Cl_clus100482_Contig1	cucumis sativus 60s ribosomal protein l23a-like mrna	809	6	5.60E-50	76.50%
465	Cl_clus100483_Contig1	---NA---	656	0 -	-	
466	Cl_clus100484_Contig1	---NA---	807	0 -	-	
467	Cl_clus100485_Contig1	---NA---	460	0 -	-	
468	Cl_clus100486_Contig1	---NA---	843	0 -	-	
469	Cl_clus100487_Contig1	gryllus bimaculatus gbcontig31584	887	1	4.70E-102	78.00%
470	Cl_clus100488_Contig1	---NA---	928	0 -	-	
471	Cl_clus100489_Contig1	---NA---	1116	0 -	-	
472	Cl_clus100490_Contig1	anolis carolinensis protein phosphatase regulatory subunit 12a mrna	708	1	1.40E-12	77.00%
473	Cl_clus100491_Contig1	---NA---	366	0 -	-	
474	Cl_clus100493_Contig2	---NA---	458	0 -	-	
475	Cl_clus100494_Contig1	---NA---	951	0 -	-	
476	Cl_clus100495_Contig1	drosophila melanogaster tropomyosin 2 transcript variant mrna	772	20	0.00E+00	79.10%
477	Cl_clus100492_Contig1	---NA---	709	0 -	-	
478	Cl_clus100496_Contig1	---NA---	1079	0 -	-	
479	Cl_clus100497_Contig1	gryllus bimaculatus gbcontig19906	1324	1	4.20E-168	80.00%
480	Cl_clus100498_Contig1	drosophila melanogaster myosin heavy chain transcript variant mrna	1139	20	0.00E+00	79.65%
481	Cl_clus100499_Contig1	---NA---	909	0 -	-	
482	Cl_clus100500_Contig1	strongylocentrotus purpuratus 5 -amp-activated protein kinase subunit gamma-2-like mrna	339	1	2.20E-12	68.00%
483	Cl_clus100501_Contig1	---NA---	595	0 -	-	
484	Cl_clus100502_Contig1	---NA---	1682	0 -	-	
485	Cl_clus100503_Contig1	---NA---	486	0 -	-	
486	Cl_clus100504_Contig1	---NA---	667	0 -	-	
487	Cl_clus100505_Contig1	---NA---	591	0 -	-	
488	Cl_clus100507_Contig1	---NA---	371	0 -	-	
489	Cl_clus100508_Contig1	---NA---	774	0 -	-	
490	Cl_clus100509_Contig1	---NA---	575	0 -	-	
491	Cl_clus100510_Contig1	---NA---	614	0 -	-	
492	Cl_clus100512_Contig1	---NA---	735	0 -	-	
493	Cl_clus100513_Contig1	---NA---	695	0 -	-	
494	Cl_clus100515_Contig1	---NA---	893	0 -	-	
495	Cl_clus100516_Contig1	---NA---	603	0 -	-	
496	Cl_clus100511_Contig1	---NA---	267	0 -	-	
497	Cl_clus100514_Contig1	---NA---	480	0 -	-	
498	Cl_clus100517_Contig1	---NA---	293	0 -	-	
499	Cl_clus100519_Contig1	---NA---	1042	0 -	-	
500	Cl_clus100520_Contig1	---NA---	391	0 -	-	
501	Cl_clus100506_Contig1	---NA---	752	0 -	-	

502	Cl_clus100521_Contig1	---NA---	490	0 -	-	
503	Cl_clus100518_Contig1	---NA---	975	0 -	-	
504	Cl_clus100522_Contig1	---NA---	667	0 -	-	
505	Cl_clus100524_Contig1	---NA---	516	0 -	-	
506	Cl_clus100525_Contig1	---NA---	535	0 -	-	
507	Cl_clus100526_Contig1	---NA---	643	0 -	-	
508	Cl_clus100527_Contig1	---NA---	722	0 -	-	
509	Cl_clus100528_Contig1	---NA---	385	0 -	-	
510	Cl_clus100529_Contig1	---NA---	863	0 -	-	
511	Cl_clus100530_Contig1	---NA---	526	0 -	-	
512	Cl_clus100531_Contig1	---NA---	664	0 -	-	
513	Cl_clus100532_Contig1	---NA---	669	0 -	-	
514	Cl_clus100523_Contig1	---NA---	793	0 -	-	
515	Cl_clus100534_Contig1	---NA---	381	0 -	-	
516	Cl_clus100535_Contig1	---NA---	914	0 -	-	
517	Cl_clus100536_Contig1	---NA---	520	0 -	-	
518	Cl_clus100537_Contig1	dermacentor variabilis isolate 40 ribosomal protein l12 complete cds	288	1	3.80E-21	69.00%
519	Cl_clus100538_Contig1	---NA---	385	0 -	-	
520	Cl_clus100539_Contig1	---NA---	377	0 -	-	
521	Cl_clus100533_Contig1	bos taurus eukaryotic translation elongation factor 1 delta transcript variant 1 complete alternatively spliced	777	5	9.00E-41	70.00%
522	Cl_clus100540_Contig1	---NA---	823	0 -	-	
523	Cl_clus100541_Contig1	---NA---	313	0 -	-	
524	Cl_clus100542_Contig1	---NA---	479	0 -	-	
525	Cl_clus100543_Contig1	---NA---	584	0 -	-	
526	Cl_clus100544_Contig1	---NA---	384	0 -	-	
527	Cl_clus100547_Contig1	---NA---	709	0 -	-	
528	Cl_clus100545_Contig1	ovis aries 40s ribosomal protein s15a-like mrna	488	20	3.20E-88	76.70%
529	Cl_clus100549_Contig1	---NA---	404	0 -	-	
530	Cl_clus100546_Contig1	---NA---	454	0 -	-	
531	Cl_clus100548_Contig1	aedes aegypti 60s ribosomal protein l23 partial mrna	472	13	4.10E-112	76.08%
532	Cl_clus100550_Contig1	---NA---	340	0 -	-	
533	Cl_clus100551_Contig1	---NA---	466	0 -	-	
534	Cl_clus100553_Contig1	---NA---	1008	0 -	-	
535	Cl_clus100554_Contig1	---NA---	1419	0 -	-	
536	Cl_clus100552_Contig1	---NA---	499	0 -	-	
537	Cl_clus100556_Contig1	---NA---	567	0 -	-	
538	Cl_clus100557_Contig1	---NA---	675	0 -	-	
539	Cl_clus100558_Contig2	---NA---	501	0 -	-	
540	Cl_clus100559_Contig1	---NA---	978	0 -	-	
541	Cl_clus100560_Contig1	---NA---	908	0 -	-	
542	Cl_clus100561_Contig1	---NA---	738	0 -	-	
543	Cl_clus100555_Contig1	gryllus bimaculatus gbcontig20780	1171	1	1.10E-16	85.00%
544	Cl_clus100562_Contig1	---NA---	1401	0 -	-	
545	Cl_clus100563_Contig1	---NA---	665	0 -	-	
546	Cl_clus100566_Contig1	---NA---	729	0 -	-	
547	Cl_clus100567_Contig1	---NA---	804	0 -	-	
548	Cl_clus100564_Contig1	---NA---	456	0 -	-	
549	Cl_clus100568_Contig1	---NA---	451	0 -	-	
550	Cl_clus100565_Contig1	---NA---	621	0 -	-	
551	Cl_clus100571_Contig1	---NA---	531	0 -	-	
552	Cl_clus100572_Contig1	---NA---	461	0 -	-	
553	Cl_clus100573_Contig1	---NA---	400	0 -	-	
554	Cl_clus100569_Contig1	---NA---	540	0 -	-	
555	Cl_clus100570_Contig1	---NA---	311	0 -	-	
556	Cl_clus100574_Contig1	---NA---	417	0 -	-	
557	Cl_clus100575_Contig1	---NA---	1292	0 -	-	
558	Cl_clus100576_Contig1	---NA---	1102	0 -	-	
559	Cl_clus100577_Contig1	fenneropenaeus chinensis glucose-regulated protein 78 complete cds	621	10	7.10E-136	74.60%
560	Cl_clus100578_Contig1	---NA---	556	0 -	-	

561	Cl_clus100579_Contig1	---NA---	769	0 -	-	
562	Cl_clus100580_Contig1	---NA---	634	0 -	-	
563	Cl_clus100581_Contig1	---NA---	318	0 -	-	
564	Cl_clus100582_Contig1	---NA---	214	0 -	-	
565	Cl_clus100583_Contig1	---NA---	468	0 -	-	
566	Cl_clus100584_Contig1	---NA---	381	0 -	-	
567	Cl_clus100585_Contig1	---NA---	563	0 -	-	
568	Cl_clus100586_Contig1	---NA---	356	0 -	-	
569	Cl_clus100587_Contig1	---NA---	379	0 -	-	
570	Cl_clus100588_Contig1	---NA---	427	0 -	-	
571	Cl_clus100589_Contig1	---NA---	550	0 -	-	
572	Cl_clus100590_Contig1	jaculus jaculus junctophilin 1 transcript variant mrna	650	20	2.40E-91	74.00%
573	Cl_clus100591_Contig1	Locusta migratoria partial mRNA for hypothetical protein (ORF19), clone lmigEST19	750	1	4.20E-32	75.00%
574	Cl_clus100593_Contig1	---NA---	766	0 -	-	
575	Cl_clus100594_Contig1	---NA---	679	0 -	-	
576	Cl_clus100595_Contig1	---NA---	658	0 -	-	
577	Cl_clus100596_Contig1	---NA---	217	0 -	-	
578	Cl_clus100592_Contig1	---NA---	909	0 -	-	
579	Cl_clus100597_Contig1	heliothis virescens secreted protein acidic and rich in cysteine-like complete sequence	892	3	4.40E-115	73.67%
580	Cl_clus100598_Contig1	---NA---	689	0 -	-	
581	Cl_clus100600_Contig1	---NA---	690	0 -	-	
582	Cl_clus100599_Contig1	---NA---	989	0 -	-	
583	Cl_clus100601_Contig1	---NA---	456	0 -	-	
584	Cl_clus100602_Contig1	---NA---	734	0 -	-	
585	Cl_clus100603_Contig1	---NA---	738	0 -	-	
586	Cl_clus100604_Contig1	---NA---	517	0 -	-	
587	Cl_clus100605_Contig1	---NA---	424	0 -	-	
588	Cl_clus100606_Contig1	---NA---	658	0 -	-	
589	Cl_clus100607_Contig1	---NA---	949	0 -	-	
590	Cl_clus100608_Contig1	---NA---	779	0 -	-	
591	Cl_clus100609_Contig1	---NA---	746	0 -	-	
592	Cl_clus100610_Contig1	---NA---	579	0 -	-	
593	Cl_clus100611_Contig1	---NA---	789	0 -	-	
594	Cl_clus100614_Contig1	---NA---	534	0 -	-	
595	Cl_clus100615_Contig1	---NA---	1162	0 -	-	
596	Cl_clus100612_Contig1	---NA---	890	0 -	-	
597	Cl_clus100613_Contig1	---NA---	785	0 -	-	
598	Cl_clus100616_Contig1	---NA---	418	0 -	-	
599	Cl_clus100617_Contig1	---NA---	719	0 -	-	
600	Cl_clus100618_Contig1	---NA---	386	0 -	-	
601	Cl_clus100619_Contig1	---NA---	322	0 -	-	
602	Cl_clus100620_Contig1	---NA---	807	0 -	-	
603	Cl_clus100621_Contig1	---NA---	303	0 -	-	
604	Cl_clus100622_Contig1	---NA---	545	0 -	-	
605	Cl_clus100623_Contig1	callorhinchus milii clone ribosomal protein l34 complete cds	660	15	6.60E-86	79.80%
606	Cl_clus100624_Contig1	plutella xylostella mrna for ribosomal protein complete cds	512	1	9.80E-89	78.00%
607	Cl_clus100625_Contig1	---NA---	276	0 -	-	
608	Cl_clus100626_Contig1	---NA---	535	0 -	-	
609	Cl_clus100627_Contig1	---NA---	587	0 -	-	
610	Cl_clus100628_Contig1	---NA---	221	0 -	-	
611	Cl_clus100629_Contig1	---NA---	613	0 -	-	
612	Cl_clus100631_Contig1	---NA---	396	0 -	-	
613	Cl_clus100632_Contig1	---NA---	532	0 -	-	
614	Cl_clus100633_Contig1	---NA---	855	0 -	-	
615	Cl_clus100634_Contig1	---NA---	567	0 -	-	
616	Cl_clus100635_Contig1	---NA---	454	0 -	-	
617	Cl_clus100636_Contig1	---NA---	840	0 -	-	
618	Cl_clus100637_Contig1	---NA---	363	0 -	-	

619	Cl_clus100638_Contig1	---NA---	377	0 -	-	
620	Cl_clus100639_Contig1	bombus terrestris 60s ribosomal protein l9-like mrna	610	2	1.20E-107	75.00%
621	Cl_clus100640_Contig1	---NA---	286	0 -	-	
622	Cl_clus100641_Contig1	---NA---	540	0 -	-	
623	Cl_clus100642_Contig1	papilio xuthus mrna for ribosomal protein complete sequence id: px-0166	323	2	2.50E-49	78.00%
624	Cl_clus100643_Contig1	---NA---	1158	0 -	-	
625	Cl_clus100644_Contig1	---NA---	497	0 -	-	
626	Cl_clus100645_Contig1	papilio polytes mrna for atpase complete sequence id: pp-0109	315	10	6.20E-19	83.80%
627	Cl_clus100646_Contig1	---NA---	837	0 -	-	
628	Cl_clus100647_Contig1	---NA---	1533	0 -	-	
629	Cl_clus100648_Contig1	anopheles gambiae pest agap010147-pa complete cds	869	20	0.00E+00	81.45%
630	Cl_clus100649_Contig1	nasonia vitripennis muscle lim protein transcript variant mrna	2180	2	1.30E-151	73.50%
631	Cl_clus100651_Contig1	---NA---	779	0 -	-	
632	Cl_clus100653_Contig1	---NA---	701	0 -	-	
633	Cl_clus100652_Contig1	---NA---	620	0 -	-	
634	Cl_clus100654_Contig1	---NA---	1143	0 -	-	
635	Cl_clus100655_Contig1	---NA---	296	0 -	-	
636	Cl_clus100656_Contig1	---NA---	256	0 -	-	
637	Cl_clus100657_Contig1	---NA---	768	0 -	-	
638	Cl_clus100658_Contig1	---NA---	413	0 -	-	
639	Cl_clus100659_Contig1	---NA---	869	0 -	-	
640	Cl_clus100660_Contig1	---NA---	485	0 -	-	
641	Cl_clus100663_Contig1	anopheles gambiae pest agap010750-pa complete cds	413	1	3.80E-99	80.00%
642	Cl_clus100664_Contig1	---NA---	538	0 -	-	
643	Cl_clus100630_Contig1	helicoverpa armigera heat shock protein 90 complete cds	961	20	1.70E-171	79.05%
644	Cl_clus100665_Contig1	---NA---	802	0 -	-	
645	Cl_clus100667_Contig1	---NA---	532	0 -	-	
646	Cl_clus100668_Contig1	entoria okinawaensis mitochondrial complete genome	545	20	1.20E-157	77.05%
647	Cl_clus100669_Contig1	---NA---	724	0 -	-	

648	Cl_clus100670_Contig1	---NA---	533	0 -	-	
649	Cl_clus100671_Contig1	---NA---	579	0 -	-	
650	Cl_clus100672_Contig1	papilio xuthus mrna for adp ribosylation complete sequence id: px-0305	501	10	9.60E-70	78.30%
651	Cl_clus100673_Contig1	---NA---	448	0 -	-	
652	Cl_clus100674_Contig1	acyrthosiphon pisum clone: full-insert cdna sequence based on ests (5 -est: 3 -est aci2aaf3yo12bbm1)	355	1	7.60E-25	75.00%
653	Cl_clus100675_Contig1	---NA---	1001	0 -	-	
654	Cl_clus100676_Contig1	---NA---	731	0 -	-	
655	Cl_clus100677_Contig1	---NA---	699	0 -	-	
656	Cl_clus100678_Contig1	---NA---	287	0 -	-	
657	Cl_clus100679_Contig1	---NA---	653	0 -	-	
658	Cl_clus100680_Contig1	riptortus pedestris mrna for ribosomal protein complete sequence id: rped-0117	259	1	2.10E-17	72.00%
659	Cl_clus100681_Contig1	callorhinchus milii clone 40s ribosomal protein s11 complete cds	444	13	1.10E-93	79.69%
660	Cl_clus100682_Contig1	---NA---	1183	0 -	-	
661	Cl_clus100650_Contig1	---NA---	952	0 -	-	
662	Cl_clus100683_Contig1	---NA---	778	0 -	-	
663	Cl_clus100684_Contig1	---NA---	351	0 -	-	
664	Cl_clus100685_Contig1	neohirasea japonica mitochondrial partial genome	839	20	0.00E+00	84.10%
665	Cl_clus100687_Contig1	nasonia vitripennis 60s ribosomal protein l18a- transcript variant 2 mrna	597	3	6.80E-98	75.33%
666	Cl_clus100688_Contig1	diaphorina citri ribosomal protein l19e complete cds	634	20	9.50E-122	77.60%
667	Cl_clus100689_Contig1	---NA---	500	0 -	-	
668	Cl_clus100690_Contig1	---NA---	1041	0 -	-	
669	Cl_clus100691_Contig1	---NA---	1342	0 -	-	
670	Cl_clus100692_Contig1	---NA---	633	0 -	-	
671	Cl_clus100693_Contig1	---NA---	718	0 -	-	
672	Cl_clus100694_Contig1	papilio xuthus mrna for cg6803- complete clone: psg25	611	1	1.30E-18	76.00%
673	Cl_clus100695_Contig1	---NA---	767	0 -	-	

674	Cl_clus100697_Contig1	---NA---	974	0	-	-
675	Cl_clus100698_Contig1	---NA---	748	0	-	-
676	Cl_clus100699_Contig1	gryllus bimaculatus gbcontig07916	579	1	2.80E-20	87.00%
677	Cl_clus100696_Contig1	---NA---	662	0	-	-
678	Cl_clus100700_Contig1	monodelphis domestica 60s ribosomal protein l26-like mrna	488	3	4.80E-86	75.33%
679	Cl_clus100701_Contig1	---NA---	402	0	-	-
680	Cl_clus100705_Contig1	---NA---	474	0	-	-
681	Cl_clus100704_Contig1	---NA---	1675	0	-	-
682	Cl_clus100706_Contig1	---NA---	604	0	-	-
683	Cl_clus100707_Contig1	---NA---	1326	0	-	-
684	Cl_clus100709_Contig1	---NA---	533	0	-	-
685	Cl_clus100710_Contig1	---NA---	792	0	-	-
686	Cl_clus100711_Contig1	monochamus alternatus adenine nucleotide translocase complete cds	1739	20	4.90E-175	70.80%
687	Cl_clus100712_Contig1	---NA---	905	0	-	-
688	Cl_clus100713_Contig1	---NA---	958	0	-	-
689	Cl_clus100714_Contig1	locusta migratoria isolate ii-11 csp partial cds	615	4	2.30E-15	68.75%
690	Cl_clus100716_Contig1	neohirasea japonica mitochondrial partial genome	1648	20	1.40E-124	84.65%
691	Cl_clus100717_Contig1	---NA---	334	0	-	-
692	Cl_clus100718_Contig1	---NA---	587	0	-	-
693	Cl_clus100719_Contig1	---NA---	820	0	-	-
694	Cl_clus100720_Contig1	---NA---	427	0	-	-
695	Cl_clus100721_Contig1	---NA---	712	0	-	-
696	Cl_clus100722_Contig1	---NA---	613	0	-	-
697	Cl_clus100723_Contig1	---NA---	446	0	-	-
698	Cl_clus100724_Contig1	---NA---	282	0	-	-
699	Cl_clus100725_Contig1	---NA---	547	0	-	-
700	Cl_clus100726_Contig1	---NA---	296	0	-	-
701	Cl_clus100728_Contig1	---NA---	763	0	-	-
702	Cl_clus100729_Contig1	---NA---	653	0	-	-
703	Cl_clus100730_Contig1	---NA---	637	0	-	-
704	Cl_clus100727_Contig1	---NA---	343	0	-	-
705	Cl_clus100731_Contig1	---NA---	382	0	-	-
706	Cl_clus100732_Contig1	---NA---	257	0	-	-
707	Cl_clus100733_Contig1	oryctolagus cuniculus ribosomal protein l10a-like mrna	836	17	2.00E-144	73.35%
708	Cl_clus100734_Contig1	---NA---	823	0	-	-
709	Cl_clus100735_Contig1	---NA---	315	0	-	-
710	Cl_clus100736_Contig1	---NA---	393	0	-	-
711	Cl_clus100737_Contig1	tribolium castaneum z9 acyl- desaturase b mrna	329	1	4.40E-21	71.00%
712	Cl_clus100738_Contig1	---NA---	240	0	-	-
713	Cl_clus100739_Contig1	---NA---	882	0	-	-
714	Cl_clus100740_Contig1	drosophila melanogaster mip20342 full insert cdna	465	10	1.70E-15	70.00%
715	Cl_clus100741_Contig1	---NA---	661	0	-	-
716	Cl_clus100742_Contig1	---NA---	564	0	-	-
717	Cl_clus100743_Contig1	gryllus bimaculatus gbcontig30540	606	1	5.30E-17	81.00%
718	Cl_clus100702_Contig1	---NA---	395	0	-	-
719	Cl_clus100708_Contig1	---NA---	516	0	-	-
720	Cl_clus100703_Contig1	gryllus bimaculatus gbcontig20780	691	1	6.10E-17	85.00%
721	Cl_clus100744_Contig1	---NA---	532	0	-	-
722	Cl_clus100745_Contig1	ornithorhynchus anatinus 40s ribosomal protein s10-like mrna	402	3	1.80E-52	74.67%
723	Cl_clus100746_Contig1	ictalurus furcatus clone cbzc3255 guanine nucleotide-binding protein subunit beta-2-like 1 complete cds	535	1	2.00E-110	77.00%
724	Cl_clus100747_Contig1	---NA---	316	0	-	-
725	Cl_clus100748_Contig1	cryptocercus punctulatus ribosomal protein s18 complete cds	592	5	1.10E-107	70.40%
726	Cl_clus100749_Contig1	---NA---	358	0	-	-
727	Cl_clus100750_Contig1	---NA---	878	0	-	-
728	Cl_clus100751_Contig1	ixodes scapularis cytochrome mrna	649	4	1.60E-74	80.25%
729	Cl_clus100752_Contig1	---NA---	693	0	-	-
730	Cl_clus100753_Contig1	---NA---	706	0	-	-
731	Cl_clus100754_Contig1	---NA---	383	0	-	-
732	Cl_clus100755_Contig1	---NA---	410	0	-	-

733	Cl_clus100756_Contig1	---NA---	581	0	-	-
734	Cl_clus100757_Contig1	---NA---	265	0	-	-
735	Cl_clus100758_Contig1	---NA---	497	0	-	-
736	Cl_clus100759_Contig1	---NA---	758	0	-	-
737	Cl_clus100760_Contig1	---NA---	476	0	-	-
738	Cl_clus100761_Contig1	---NA---	857	0	-	-
739	Cl_clus100762_Contig1	---NA---	616	0	-	-
740	Cl_clus100763_Contig1	---NA---	334	0	-	-
741	Cl_clus100765_Contig1	---NA---	291	0	-	-
742	Cl_clus100766_Contig1	---NA---	398	0	-	-
743	Cl_clus100767_Contig1	---NA---	787	0	-	-
744	Cl_clus100768_Contig1	---NA---	467	0	-	-
745	Cl_clus100764_Contig1	---NA---	276	0	-	-
746	Cl_clus100769_Contig1	---NA---	253	0	-	-
747	Cl_clus100770_Contig1	---NA---	322	0	-	-
748	Cl_clus100771_Contig1	---NA---	371	0	-	-
749	Cl_clus100772_Contig1	---NA---	548	0	-	-
750	Cl_clus100773_Contig1	---NA---	734	0	-	-
751	Cl_clus100774_Contig1	---NA---	435	0	-	-
752	Cl_clus100775_Contig1	---NA---	443	0	-	-
753	Cl_clus100776_Contig1	---NA---	465	0	-	-
754	Cl_clus100777_Contig1	---NA---	721	0	-	-
755	Cl_clus100778_Contig1	---NA---	520	0	-	-
756	Cl_clus100779_Contig1	oryzias latipes tumor suppressor candidate 3-like mrna	771	1	1.00E-52	69.00%
757	Cl_clus100780_Contig1	---NA---	285	0	-	-
758	Cl_clus100781_Contig1	---NA---	241	0	-	-
759	Cl_clus100782_Contig1	---NA---	368	0	-	-
760	Cl_clus100783_Contig1	---NA---	469	0	-	-
761	Cl_clus100784_Contig1	---NA---	449	0	-	-
762	Cl_clus100785_Contig1	aplysia californica nadh-ubiquinone oxidoreductase 75 kda mitochondrial-like transcript variant mrna	613	17	1.00E-152	75.41%
763	Cl_clus100786_Contig1	---NA---	592	0	-	-
764	Cl_clus100787_Contig1	---NA---	504	0	-	-
765	Cl_clus100788_Contig1	---NA---	412	0	-	-
766	Cl_clus100789_Contig1	---NA---	751	0	-	-
767	Cl_clus100790_Contig1	---NA---	362	0	-	-
768	Cl_clus100791_Contig1	---NA---	372	0	-	-
769	Cl_clus100792_Contig1	---NA---	600	0	-	-
770	Cl_clus100793_Contig1	---NA---	320	0	-	-
771	Cl_clus100794_Contig1	---NA---	301	0	-	-
772	Cl_clus100795_Contig1	---NA---	393	0	-	-
773	Cl_clus100796_Contig1	---NA---	280	0	-	-
774	Cl_clus100797_Contig1	---NA---	210	0	-	-
775	Cl_clus100799_Contig1	gryllus bimaculatus gbcontig06341	343	1	1.50E-14	80.00%
776	Cl_clus100798_Contig1	---NA---	800	0	-	-
777	Cl_clus100800_Contig1	---NA---	677	0	-	-
778	Cl_clus100801_Contig1	---NA---	459	0	-	-
779	Cl_clus100802_Contig1	---NA---	484	0	-	-
780	Cl_clus100803_Contig1	---NA---	379	0	-	-
781	Cl_clus100804_Contig1	---NA---	330	0	-	-
782	Cl_clus100805_Contig1	---NA---	373	0	-	-
783	Cl_clus100806_Contig1	---NA---	962	0	-	-
784	Cl_clus100807_Contig1	---NA---	757	0	-	-
785	Cl_clus100809_Contig1	---NA---	871	0	-	-
786	Cl_clus100810_Contig1	tribolium castaneum agap010429-pa mrna	393	1	2.30E-76	78.00%
787	Cl_clus100811_Contig1	---NA---	382	0	-	-
788	Cl_clus100808_Contig1	---NA---	301	0	-	-
789	Cl_clus100812_Contig1	---NA---	687	0	-	-
790	Cl_clus100813_Contig1	---NA---	332	0	-	-

791	Cl_clus100814_Contig1	---NA---	423	0 -	-	
792	Cl_clus100815_Contig1	plutella xylostella ryanodine receptor complete cds	899	8	0.00E+00	76.88%
793	Cl_clus100816_Contig1	---NA---	328	0 -	-	
794	Cl_clus100817_Contig1	---NA---	537	0 -	-	
795	Cl_clus100818_Contig1	---NA---	317	0 -	-	
796	Cl_clus100819_Contig1	---NA---	370	0 -	-	
797	Cl_clus100820_Contig1	---NA---	381	0 -	-	
798	Cl_clus100821_Contig1	---NA---	688	0 -	-	
799	Cl_clus100822_Contig1	---NA---	343	0 -	-	
800	Cl_clus100823_Contig1	---NA---	871	0 -	-	
801	Cl_clus100824_Contig1	---NA---	417	0 -	-	
802	Cl_clus100825_Contig1	nomascus leucogenys s-phase kinase-associated protein 1 mrna	301	20	2.30E-49	78.00%
803	Cl_clus100826_Contig1	---NA---	681	0 -	-	
804	Cl_clus100827_Contig1	---NA---	659	0 -	-	
805	Cl_clus100828_Contig1	salmo salar aspartate cytoplasmic mrna	630	6	7.30E-60	71.17%
806	Cl_clus100829_Contig1	---NA---	228	0 -	-	
807	Cl_clus100830_Contig1	---NA---	1053	0 -	-	
808	Cl_clus100831_Contig1	---NA---	285	0 -	-	
809	Cl_clus100832_Contig1	---NA---	406	0 -	-	
810	Cl_clus100833_Contig1	---NA---	489	0 -	-	
811	Cl_clus100834_Contig1	---NA---	808	0 -	-	
812	Cl_clus100835_Contig1	helicoverpa armigera pupae dna	577	1	1.30E-24	88.00%
813	Cl_clus100836_Contig1	---NA---	566	0 -	-	
814	Cl_clus100837_Contig1	---NA---	226	0 -	-	
815	Cl_clus100838_Contig1	---NA---	417	0 -	-	
816	Cl_clus100839_Contig1	---NA---	605	0 -	-	
817	Cl_clus100840_Contig1	---NA---	415	0 -	-	
818	Cl_clus100842_Contig1	---NA---	454	0 -	-	
819	Cl_clus100843_Contig1	---NA---	319	0 -	-	
820	Cl_clus100844_Contig1	---NA---	291	0 -	-	
821	Cl_clus100845_Contig1	candida albicans sc5314 histone h3 complete cds	582	20	6.20E-92	77.45%
822	Cl_clus100846_Contig1	---NA---	345	0 -	-	
823	Cl_clus100847_Contig1	---NA---	660	0 -	-	
824	Cl_clus100848_Contig1	echinops telfairi nadh dehydrogenase flavoprotein 51kda transcript variant mrna	981	20	0.00E+00	75.25%
825	Cl_clus100849_Contig1	---NA---	606	0 -	-	
826	Cl_clus100850_Contig1	---NA---	502	0 -	-	
827	Cl_clus100841_Contig1	---NA---	478	0 -	-	
828	Cl_clus100851_Contig1	---NA---	407	0 -	-	
829	Cl_clus100852_Contig1	---NA---	977	0 -	-	
830	Cl_clus100853_Contig1	---NA---	506	0 -	-	
831	Cl_clus100854_Contig1	---NA---	710	0 -	-	
832	Cl_clus100855_Contig1	maylandia zebra 40s ribosomal protein s3-like mrna	265	5	1.20E-58	79.20%
833	Cl_clus100856_Contig1	---NA---	498	0 -	-	
834	Cl_clus100857_Contig1	---NA---	558	0 -	-	
835	Cl_clus100858_Contig1	---NA---	850	0 -	-	
836	Cl_clus100859_Contig1	---NA---	552	0 -	-	
837	Cl_clus100860_Contig1	---NA---	420	0 -	-	
838	Cl_clus100861_Contig1	---NA---	559	0 -	-	
839	Cl_clus100862_Contig1	---NA---	511	0 -	-	
840	Cl_clus100863_Contig1	---NA---	753	0 -	-	
841	Cl_clus100864_Contig1	---NA---	382	0 -	-	
842	Cl_clus100865_Contig1	---NA---	430	0 -	-	
843	Cl_clus100866_Contig1	---NA---	415	0 -	-	
844	Cl_clus100867_Contig1	---NA---	850	0 -	-	
845	Cl_clus100868_Contig1	---NA---	315	0 -	-	
846	Cl_clus100869_Contig1	Locusta migratoria partial mRNA for hypothetical protein (ORF10), clone lmigEST10	388	1	2.10E-32	74.00%
847	Cl_clus100870_Contig1	nasonia vitripennis 40s ribosomal protein s9-like mrna	727	2	2.00E-118	75.00%
848	Cl_clus100871_Contig1	---NA---	575	0 -	-	

849	Cl_clus100872_Contig1	---NA---	429	0 -	-	
850	Cl_clus100873_Contig1	---NA---	503	0 -	-	
851	Cl_clus100874_Contig1	---NA---	346	0 -	-	
852	Cl_clus100875_Contig1	---NA---	483	0 -	-	
853	Cl_clus100876_Contig1	---NA---	209	0 -	-	
854	Cl_clus100877_Contig1	---NA---	658	0 -	-	
855	Cl_clus100878_Contig1	---NA---	415	0 -	-	
856	Cl_clus100879_Contig1	---NA---	394	0 -	-	
857	Cl_clus100880_Contig1	bombus terrestris 40s ribosomal protein s29-like mrna	400	1	2.20E-32	78.00%
858	Cl_clus100881_Contig1	---NA---	754	0 -	-	
859	Cl_clus100882_Contig1	---NA---	371	0 -	-	
860	Cl_clus100883_Contig1	---NA---	365	0 -	-	
861	Cl_clus100884_Contig1	---NA---	1313	0 -	-	
862	Cl_clus100886_Contig1	---NA---	465	0 -	-	
863	Cl_clus100887_Contig1	---NA---	338	0 -	-	
864	Cl_clus100888_Contig1	---NA---	723	0 -	-	
865	Cl_clus100885_Contig1	---NA---	405	0 -	-	
866	Cl_clus100891_Contig1	---NA---	296	0 -	-	
867	Cl_clus100892_Contig1	---NA---	827	0 -	-	
868	Cl_clus100893_Contig1	---NA---	766	0 -	-	
869	Cl_clus100889_Contig1	---NA---	486	0 -	-	
870	Cl_clus100894_Contig1	---NA---	652	0 -	-	
871	Cl_clus100895_Contig1	---NA---	567	0 -	-	
872	Cl_clus100896_Contig1	---NA---	745	0 -	-	
873	Cl_clus100890_Contig1	---NA---	246	0 -	-	
874	Cl_clus100897_Contig1	---NA---	685	0 -	-	
875	Cl_clus100898_Contig1	---NA---	741	0 -	-	
876	Cl_clus100899_Contig1	---NA---	474	0 -	-	
877	Cl_clus100900_Contig1	---NA---	559	0 -	-	
878	Cl_clus100901_Contig1	drosophila virilis gj11017 (dvir\gj11017) mrna	265	1	3.90E-14	68.00%
879	Cl_clus100902_Contig1	---NA---	525	0 -	-	
880	Cl_clus100903_Contig1	---NA---	428	0 -	-	
881	Cl_clus100904_Contig1	---NA---	772	0 -	-	
882	Cl_clus100905_Contig1	---NA---	508	0 -	-	
883	Cl_clus100906_Contig1	---NA---	531	0 -	-	
884	Cl_clus100907_Contig1	---NA---	748	0 -	-	
885	Cl_clus100908_Contig1	drosophila willistoni gk23837 (dwil\gk23837) mrna	303	1	8.80E-17	82.00%
886	Cl_clus100909_Contig1	---NA---	306	0 -	-	
887	Cl_clus100911_Contig1	drosophila melanogaster s-adenosylmethionine synthetase (sam-s) transcript variant mrna	386	20	7.70E-57	74.50%
888	Cl_clus100912_Contig1	---NA---	565	0 -	-	
889	Cl_clus100910_Contig1	---NA---	494	0 -	-	
890	Cl_clus100913_Contig1	---NA---	616	0 -	-	
891	Cl_clus100914_Contig1	---NA---	420	0 -	-	
892	Cl_clus100915_Contig1	---NA---	529	0 -	-	
893	Cl_clus100916_Contig1	---NA---	310	0 -	-	
894	Cl_clus100917_Contig1	---NA---	536	0 -	-	
895	Cl_clus100918_Contig1	---NA---	625	0 -	-	
896	Cl_clus100919_Contig1	---NA---	619	0 -	-	
897	Cl_clus100920_Contig1	---NA---	348	0 -	-	
898	Cl_clus100921_Contig1	---NA---	448	0 -	-	
899	Cl_clus100922_Contig1	---NA---	335	0 -	-	
900	Cl_clus100923_Contig1	---NA---	1039	0 -	-	
901	Cl_clus100924_Contig1	---NA---	280	0 -	-	
902	Cl_clus100925_Contig1	meladema coriacea mrna for ribosomal protein l7ae (rpl7ae gene)	470	1	3.30E-56	73.00%
903	Cl_clus100926_Contig1	papilio xuthus mrna for vacuolar h	434	1	1.60E-53	78.00%
904	Cl_clus100927_Contig1	---NA---	447	0 -	-	
905	Cl_clus100928_Contig1	acanthoxyla prasina isolate cytochrome oxidase subunit i and cytochrome oxidase subunit ii partial cds mitochondrial	433	20	0.00E+00	99.65%
906	Cl_clus100929_Contig1	---NA---	1491	0 -	-	

907	Cl_clus100930_Contig1	---NA---	458	0 -	-	
908	Cl_clus100931_Contig1	---NA---	647	0 -	-	
909	Cl_clus100932_Contig1	vitis vinifera ubiquitin-conjugating enzyme e2-17 kda- transcript variant 1 mrna	1158	11	5.50E-52	73.09%
910	Cl_clus100933_Contig1	cnaphalocrocis medinalis ryanodine receptor complete cds	483	2	1.50E-73	72.00%
911	Cl_clus100934_Contig1	---NA---	1003	0 -	-	
912	Cl_clus100935_Contig1	---NA---	832	0 -	-	
913	Cl_clus100936_Contig1	---NA---	727	0 -	-	
914	Cl_clus100937_Contig1	---NA---	668	0 -	-	
915	Cl_clus100938_Contig1	---NA---	338	0 -	-	
916	Cl_clus100939_Contig1	callithrix jacchus 60s ribosomal protein l27a-like mrna	543	15	8.60E-71	73.07%
917	Cl_clus100941_Contig1	---NA---	725	0 -	-	
918	Cl_clus100942_Contig1	---NA---	487	0 -	-	
919	Cl_clus100940_Contig1	---NA---	639	0 -	-	
920	Cl_clus100943_Contig1	---NA---	204	0 -	-	
921	Cl_clus100944_Contig1	---NA---	333	0 -	-	
922	Cl_clus100947_Contig1	---NA---	485	0 -	-	
923	Cl_clus100945_Contig1	---NA---	649	0 -	-	
924	Cl_clus100946_Contig1	---NA---	459	0 -	-	
925	Cl_clus100948_Contig1	---NA---	376	0 -	-	
926	Cl_clus100949_Contig1	---NA---	724	0 -	-	
927	Cl_clus100950_Contig1	---NA---	656	0 -	-	
928	Cl_clus100951_Contig1	---NA---	506	0 -	-	
929	Cl_clus100952_Contig1	gryllus bimaculatus gbcontig28204	943	14	6.10E-120	72.71%
930	Cl_clus100953_Contig1	---NA---	415	0 -	-	
931	Cl_clus100954_Contig1	---NA---	551	0 -	-	
932	Cl_clus100955_Contig1	---NA---	361	0 -	-	
933	Cl_clus100956_Contig1	---NA---	582	0 -	-	
934	Cl_clus100957_Contig1	---NA---	494	0 -	-	
935	Cl_clus100959_Contig1	---NA---	682	0 -	-	

936	Cl_clus100958_Contig1	---NA---	442	0 -	-	
937	Cl_clus100960_Contig1	drosophila melanogaster ryanodine receptor 44f (rya-r44f) transcript variant mrna	583	20	2.50E-179	82.90%
938	Cl_clus100961_Contig1	---NA---	499	0 -	-	
939	Cl_clus100962_Contig1	---NA---	371	0 -	-	
940	Cl_clus100963_Contig1	---NA---	317	0 -	-	
941	Cl_clus100964_Contig1	---NA---	351	0 -	-	
942	Cl_clus100966_Contig1	---NA---	764	0 -	-	
943	Cl_clus100967_Contig1	echinops telfairi liver mrna	647	6	1.50E-68	71.17%
944	Cl_clus100968_Contig1	apis florea protein disulfide-isomerase a3-like mrna	901	4	5.50E-38	76.50%
945	Cl_clus100969_Contig1	---NA---	671	0 -	-	
946	Cl_clus100971_Contig1	telmatoscopus amf-2003 ribosomal protein s6 partial cds	646	1	3.00E-52	71.00%
947	Cl_clus100972_Contig1	---NA---	768	0 -	-	
948	Cl_clus100973_Contig1	---NA---	897	0 -	-	
949	Cl_clus100974_Contig1	pan troglodytes ribosomal protein l8 mrna	516	20	1.20E-87	75.00%
950	Cl_clus100975_Contig1	---NA---	491	0 -	-	
951	Cl_clus100970_Contig1	---NA---	265	0 -	-	
952	Cl_clus100977_Contig1	---NA---	694	0 -	-	
953	Cl_clus100978_Contig1	---NA---	353	0 -	-	
954	Cl_clus100979_Contig1	---NA---	212	0 -	-	
955	Cl_clus100976_Contig1	---NA---	304	0 -	-	
956	Cl_clus100965_Contig1	---NA---	273	0 -	-	
957	Cl_clus100980_Contig1	---NA---	590	0 -	-	
958	Cl_clus100981_Contig1	ailuropoda melanoleuca ribosomal protein l5 complete cds	704	13	1.60E-62	71.31%
959	Cl_clus100982_Contig1	---NA---	376	0 -	-	
960	Cl_clus100984_Contig1	---NA---	473	0 -	-	
961	Cl_clus100985_Contig1	neohirasea japonica mitochondrial partial genome	818	20	2.90E-85	78.60%
962	Cl_clus100986_Contig1	---NA---	528	0 -	-	
963	Cl_clus100987_Contig1	---NA---	833	0 -	-	
964	Cl_clus100988_Contig1	---NA---	578	0 -	-	

965	Cl_clus100989_Contig1	---NA---	791	0	-	-
966	Cl_clus100990_Contig1	---NA---	637	0	-	-
967	Cl_clus100991_Contig1	---NA---	678	0	-	-
968	Cl_clus100983_Contig1	---NA---	465	0	-	-
969	Cl_clus100993_Contig1	---NA---	690	0	-	-
970	Cl_clus100995_Contig1	---NA---	991	0	-	-
971	Cl_clus100996_Contig1	---NA---	560	0	-	-
972	Cl_clus100997_Contig1	---NA---	865	0	-	-
973	Cl_clus100994_Contig1	---NA---	663	0	-	-
974	Cl_clus100998_Contig1	---NA---	399	0	-	-
975	Cl_clus100999_Contig1	triatoma vitticeps arginine kinase complete cds	1787	20	0.00E+00	76.45%
976	Cl_clus100992_Contig1	---NA---	849	0	-	-
977	Cl_clus101000_Contig1	---NA---	603	0	-	-
978	Cl_clus101002_Contig1	papio anubis ubiquitin transcript variant 12 mrna	311	20	1.70E-95	85.00%
979	Cl_clus101003_Contig1	periplaneta americana cxpwmw05 complete cds	758	1	5.20E-12	94.00%
980	Cl_clus101004_Contig1	extatosoma tiaratum mitochondrial complete genome	827	20	5.00E-76	82.50%
981	Cl_clus101005_Contig1	---NA---	649	0	-	-
982	Cl_clus101001_Contig1	---NA---	605	0	-	-
983	Cl_clus101006_Contig1	---NA---	216	0	-	-
984	Cl_clus101008_Contig1	---NA---	328	0	-	-
985	Cl_clus101009_Contig1	---NA---	548	0	-	-
986	Cl_clus101010_Contig1	---NA---	316	0	-	-
987	Cl_clus101007_Contig1	---NA---	1227	0	-	-
988	Cl_clus101011_Contig1	---NA---	692	0	-	-
989	Cl_clus101012_Contig1	---NA---	544	0	-	-
990	Cl_clus101013_Contig1	---NA---	485	0	-	-
991	Cl_clus101014_Contig1	---NA---	317	0	-	-
992	Cl_clus101015_Contig1	---NA---	754	0	-	-
993	Cl_clus101016_Contig1	---NA---	229	0	-	-
994	Cl_clus101017_Contig1	---NA---	1179	0	-	-
995	Cl_clus101018_Contig1	---NA---	500	0	-	-
996	Cl_clus101019_Contig1	---NA---	714	0	-	-
997	Cl_clus101020_Contig1	---NA---	297	0	-	-
998	Cl_clus101021_Contig1	---NA---	491	0	-	-
999	Cl_clus101022_Contig1	---NA---	646	0	-	-
1000	Cl_clus101023_Contig1	---NA---	329	0	-	-
1001	Cl_clus101025_Contig1	---NA---	604	0	-	-
1002	Cl_clus101027_Contig1	---NA---	464	0	-	-
1003	Cl_clus101028_Contig1	---NA---	561	0	-	-
1004	Cl_clus101031_Contig1	rhipicephalus sanguineus calreticulin complete cds	878	11	1.60E-158	75.45%
1005	Cl_clus101029_Contig1	---NA---	296	0	-	-
1006	Cl_clus101024_Contig1	---NA---	220	0	-	-
1007	Cl_clus101030_Contig1	---NA---	216	0	-	-
1008	Cl_clus101026_Contig1	---NA---	387	0	-	-
1009	Cl_clus101032_Contig1	---NA---	385	0	-	-
1010	Cl_clus101033_Contig1	---NA---	867	0	-	-
1011	Cl_clus101034_Contig1	nematostella vectensis protein partial mrna	520	1	9.30E-26	82.00%
1012	Cl_clus101035_Contig1	---NA---	671	0	-	-
1013	Cl_clus101036_Contig1	---NA---	606	0	-	-
1014	Cl_clus101038_Contig1	---NA---	350	0	-	-
1015	Cl_clus101039_Contig1	---NA---	533	0	-	-
1016	Cl_clus101040_Contig1	biphyllus lunatus mrna for ribosomal protein l37e (rpl37e gene)	460	1	1.10E-30	73.00%
1017	Cl_clus101037_Contig1	---NA---	331	0	-	-
1018	Cl_clus101041_Contig1	---NA---	434	0	-	-
1019	Cl_clus101042_Contig1	---NA---	343	0	-	-
1020	Cl_clus101043_Contig1	---NA---	691	0	-	-
1021	Cl_clus101044_Contig1	---NA---	362	0	-	-
1022	Cl_clus101045_Contig1	---NA---	245	0	-	-
1023	Cl_clus101046_Contig1	---NA---	548	0	-	-
1024	Cl_clus101047_Contig1	nematostella vectensis protein partial mrna	449	2	5.00E-22	77.50%

1025	Cl_clus101048_Contig1	---NA---	695	0 -	-	
1026	Cl_clus101049_Contig1	---NA---	340	0 -	-	
1027	Cl_clus101050_Contig1	---NA---	208	0 -	-	
1028	Cl_clus101051_Contig1	---NA---	504	0 -	-	
1029	Cl_clus101052_Contig1	---NA---	233	0 -	-	
1030	Cl_clus101053_Contig1	---NA---	439	0 -	-	
1031	Cl_clus101054_Contig1	---NA---	422	0 -	-	
1032	Cl_clus101055_Contig1	---NA---	324	0 -	-	
1033	Cl_clus101056_Contig1	---NA---	328	0 -	-	
1034	Cl_clus101057_Contig1	---NA---	366	0 -	-	
1035	Cl_clus101058_Contig1	---NA---	286	0 -	-	
1036	Cl_clus101059_Contig1	---NA---	204	0 -	-	
1037	Cl_clus101060_Contig1	---NA---	442	0 -	-	
1038	Cl_clus101061_Contig1	---NA---	283	0 -	-	
1039	Cl_clus101062_Contig1	---NA---	368	0 -	-	
1040	Cl_clus101063_Contig1	---NA---	520	0 -	-	
1041	Cl_clus101064_Contig1	---NA---	558	0 -	-	
1042	Cl_clus101065_Contig1	---NA---	409	0 -	-	
1043	Cl_clus101066_Contig1	---NA---	426	0 -	-	
1044	Cl_clus101067_Contig1	---NA---	464	0 -	-	
1045	Cl_clus101068_Contig1	---NA---	253	0 -	-	
1046	Cl_clus101069_Contig1	---NA---	290	0 -	-	
1047	Cl_clus101070_Contig1	---NA---	475	0 -	-	
1048	Cl_clus101071_Contig1	---NA---	356	0 -	-	
1049	Cl_clus101072_Contig1	---NA---	471	0 -	-	
1050	Cl_clus101073_Contig1	---NA---	240	0 -	-	
1051	Cl_clus101074_Contig1	---NA---	523	0 -	-	
1052	Cl_clus101075_Contig1	---NA---	777	0 -	-	
1053	Cl_clus101076_Contig1	---NA---	714	0 -	-	
1054	Cl_clus101077_Contig1	---NA---	272	0 -	-	
1055	Cl_clus101078_Contig1	---NA---	510	0 -	-	
1056	Cl_clus101079_Contig1	mesocricetus auratus partial mrna for dihydrolipoamide dehydrogenase (dld gene)	407	3	7.70E-51	70.67%
1057	Cl_clus101080_Contig1	---NA---	311	0 -	-	
1058	Cl_clus101081_Contig1	---NA---	209	0 -	-	
1059	Cl_clus101082_Contig1	chrysochromulina nies-1333 alpha tubulin partial cds	262	20	2.90E-85	86.00%
1060	Cl_clus101083_Contig1	---NA---	213	0 -	-	
1061	Cl_clus101084_Contig1	---NA---	345	0 -	-	
1062	Cl_clus101085_Contig1	---NA---	209	0 -	-	
1063	Cl_clus101086_Contig1	---NA---	391	0 -	-	
1064	Cl_clus101087_Contig1	---NA---	281	0 -	-	
1065	Cl_clus101088_Contig1	---NA---	363	0 -	-	
1066	Cl_clus101089_Contig1	---NA---	385	0 -	-	
1067	Cl_clus101090_Contig1	---NA---	392	0 -	-	
1068	Cl_clus101091_Contig1	---NA---	321	0 -	-	
1069	Cl_clus101092_Contig1	drosophila melanogaster malic enzyme b (men-b) transcript variant mrna	745	20	6.60E-55	70.60%
1070	Cl_clus101093_Contig1	---NA---	280	0 -	-	
1071	Cl_clus101094_Contig1	---NA---	410	0 -	-	
1072	Cl_clus101095_Contig1	barley mrna for aspartic proteinase	609	1	5.40E-17	77.00%
1073	Cl_clus101097_Contig1	---NA---	242	0 -	-	
1074	Cl_clus101098_Contig1	---NA---	471	0 -	-	
1075	Cl_clus101099_Contig1	---NA---	332	0 -	-	
1076	Cl_clus101100_Contig1	---NA---	378	0 -	-	
1077	Cl_clus101101_Contig1	---NA---	437	0 -	-	
1078	Cl_clus101096_Contig1	---NA---	399	0 -	-	
1079	Cl_clus101102_Contig1	---NA---	662	0 -	-	
1080	Cl_clus101103_Contig1	---NA---	1108	0 -	-	
1081	Cl_clus101104_Contig1	---NA---	356	0 -	-	
1082	Cl_clus101105_Contig1	---NA---	615	0 -	-	

1083	Cl_clus101106_Contig1	---NA---	554	0 -	-	
1084	Cl_clus101107_Contig1	---NA---	499	0 -	-	
1085	Cl_clus101109_Contig1	riptortus pedestris mrna for splicing factor u2af large complete sequence id: rped-1473	592	3	1.00E-114	75.33%
1086	Cl_clus101110_Contig1	---NA---	325	0 -	-	
1087	Cl_clus101108_Contig1	anopheles gambiae pest agap003851-pa complete cds	202	16	1.60E-36	79.13%
1088	Cl_clus101111_Contig1	---NA---	458	0 -	-	
1089	Cl_clus101112_Contig1	---NA---	484	0 -	-	
1090	Cl_clus101113_Contig1	---NA---	420	0 -	-	
1091	Cl_clus101114_Contig1	locusta migratoria hexokinase complete cds	283	1	1.90E-37	76.00%
1092	Cl_clus101115_Contig1	---NA---	310	0 -	-	
1093	Cl_clus101116_Contig1	---NA---	283	0 -	-	
1094	Cl_clus101117_Contig1	---NA---	539	0 -	-	
1095	Cl_clus101118_Contig1	---NA---	367	0 -	-	
1096	Cl_clus101119_Contig1	---NA---	412	0 -	-	
1097	Cl_clus101121_Contig1	---NA---	559	0 -	-	
1098	Cl_clus101122_Contig1	echinops telfairi valosin containing protein mrna	210	2	1.00E-32	77.00%
1099	Cl_clus101123_Contig1	---NA---	576	0 -	-	
1100	Cl_clus101124_Contig1	---NA---	326	0 -	-	
1101	Cl_clus101125_Contig1	---NA---	546	0 -	-	
1102	Cl_clus101126_Contig1	---NA---	404	0 -	-	
1103	Cl_clus101127_Contig1	---NA---	570	0 -	-	
1104	Cl_clus101120_Contig1	---NA---	416	0 -	-	
1105	Cl_clus101128_Contig1	---NA---	487	0 -	-	
1106	Cl_clus101129_Contig1	---NA---	229	0 -	-	
1107	Cl_clus101130_Contig1	---NA---	271	0 -	-	
1108	Cl_clus101131_Contig1	---NA---	306	0 -	-	
1109	Cl_clus101133_Contig1	---NA---	429	0 -	-	
1110	Cl_clus101134_Contig1	---NA---	211	0 -	-	
1111	Cl_clus101135_Contig1	ficedula albicollis ezrin transcript variant mrna	434	9	1.70E-97	75.78%
1112	Cl_clus101136_Contig1	---NA---	267	0 -	-	
1113	Cl_clus101137_Contig1	---NA---	508	0 -	-	
1114	Cl_clus101132_Contig1	---NA---	632	0 -	-	
1115	Cl_clus101138_Contig1	---NA---	418	0 -	-	
1116	Cl_clus101139_Contig1	---NA---	566	0 -	-	
1117	Cl_clus101140_Contig1	---NA---	288	0 -	-	
1118	Cl_clus101141_Contig1	---NA---	300	0 -	-	
1119	Cl_clus101142_Contig1	---NA---	257	0 -	-	
1120	Cl_clus101143_Contig1	---NA---	527	0 -	-	
1121	Cl_clus101145_Contig1	---NA---	394	0 -	-	
1122	Cl_clus101146_Contig1	---NA---	343	0 -	-	
1123	Cl_clus101144_Contig1	---NA---	515	0 -	-	
1124	Cl_clus101148_Contig1	---NA---	353	0 -	-	
1125	Cl_clus101149_Contig1	---NA---	417	0 -	-	
1126	Cl_clus101150_Contig1	---NA---	453	0 -	-	
1127	Cl_clus101151_Contig1	---NA---	251	0 -	-	
1128	Cl_clus101152_Contig1	---NA---	226	0 -	-	
1129	Cl_clus101153_Contig1	---NA---	679	0 -	-	
1130	Cl_clus101154_Contig1	gryllus bimaculatus gbcontig30018	364	1	3.80E-54	88.00%
1131	Cl_clus101155_Contig1	---NA---	332	0 -	-	
1132	Cl_clus101156_Contig1	---NA---	479	0 -	-	
1133	Cl_clus101158_Contig1	---NA---	483	0 -	-	
1134	Cl_clus101159_Contig1	---NA---	225	0 -	-	
1135	Cl_clus101160_Contig1	---NA---	580	0 -	-	
1136	Cl_clus101157_Contig1	---NA---	439	0 -	-	
1137	Cl_clus101161_Contig1	---NA---	564	0 -	-	
1138	Cl_clus101162_Contig1	---NA---	277	0 -	-	
1139	Cl_clus101163_Contig1	---NA---	436	0 -	-	
1140	Cl_clus101164_Contig1	---NA---	322	0 -	-	
1141	Cl_clus101165_Contig1	---NA---	892	0 -	-	

1142	Cl_clus101166_Contig1	drosophila melanogaster cg2010 transcript variant mrna	292	20	4.30E-90	83.40%
1143	Cl_clus101167_Contig1	---NA---	274	0 -	-	
1144	Cl_clus101168_Contig1	---NA---	270	0 -	-	
1145	Cl_clus101169_Contig1	---NA---	483	0 -	-	
1146	Cl_clus101170_Contig1	---NA---	276	0 -	-	
1147	Cl_clus101171_Contig1	---NA---	268	0 -	-	
1148	Cl_clus101172_Contig1	---NA---	317	0 -	-	
1149	Cl_clus101173_Contig1	---NA---	406	0 -	-	
1150	Cl_clus101174_Contig1	---NA---	419	0 -	-	
1151	Cl_clus101175_Contig1	---NA---	546	0 -	-	
1152	Cl_clus101176_Contig1	---NA---	229	0 -	-	
1153	Cl_clus101177_Contig1	---NA---	224	0 -	-	
1154	Cl_clus101178_Contig1	---NA---	323	0 -	-	
1155	Cl_clus101179_Contig1	gallus gallus dead (asp-glu-ala-asp) box polypeptide 43 transcript variant mrna	268	4	4.80E-13	91.00%
1156	Cl_clus101180_Contig1	---NA---	436	0 -	-	
1157	Cl_clus101182_Contig1	---NA---	259	0 -	-	
1158	Cl_clus101183_Contig1	---NA---	250	0 -	-	
1159	Cl_clus101185_Contig1	---NA---	621	0 -	-	
1160	Cl_clus101186_Contig1	---NA---	360	0 -	-	
1161	Cl_clus101187_Contig1	---NA---	355	0 -	-	
1162	Cl_clus101188_Contig1	---NA---	336	0 -	-	
1163	Cl_clus101189_Contig1	---NA---	466	0 -	-	
1164	Cl_clus101190_Contig1	---NA---	369	0 -	-	
1165	Cl_clus101192_Contig1	---NA---	803	0 -	-	
1166	Cl_clus101193_Contig1	---NA---	791	0 -	-	
1167	Cl_clus101194_Contig1	---NA---	270	0 -	-	
1168	Cl_clus101195_Contig1	---NA---	257	0 -	-	
1169	Cl_clus101196_Contig1	---NA---	237	0 -	-	
1170	Cl_clus101197_Contig1	---NA---	328	0 -	-	
1171	Cl_clus101191_Contig1	megachile rotundata ryanodine receptor 44f-like mrna	428	2	8.10E-89	75.50%
1172	Cl_clus101198_Contig1	---NA---	703	0 -	-	
1173	Cl_clus101199_Contig1	---NA---	325	0 -	-	
1174	Cl_clus101200_Contig1	---NA---	377	0 -	-	
1175	Cl_clus101201_Contig1	drosophila melanogaster ryanodine receptor 44f (rya-r44f) transcript variant mrna	627	18	1.00E-108	78.78%
1176	Cl_clus101202_Contig1	---NA---	336	0 -	-	
1177	Cl_clus101204_Contig1	---NA---	905	0 -	-	
1178	Cl_clus101203_Contig1	---NA---	343	0 -	-	
1179	Cl_clus101207_Contig1	---NA---	325	0 -	-	
1180	Cl_clus101206_Contig1	---NA---	271	0 -	-	
1181	Cl_clus101208_Contig1	---NA---	346	0 -	-	
1182	Cl_clus101210_Contig1	---NA---	224	0 -	-	
1183	Cl_clus101184_Contig1	---NA---	466	0 -	-	
1184	Cl_clus101212_Contig1	barentsia elongata ribosomal protein s13 complete cds	526	1	1.40E-80	74.00%
1185	Cl_clus101213_Contig1	---NA---	305	0 -	-	
1186	Cl_clus101214_Contig1	---NA---	358	0 -	-	
1187	Cl_clus101215_Contig1	---NA---	421	0 -	-	
1188	Cl_clus101216_Contig1	---NA---	289	0 -	-	
1189	Cl_clus101218_Contig1	---NA---	231	0 -	-	
1190	Cl_clus101219_Contig1	nasonia vitripennis rna-binding protein lark-like mrna	286	1	4.90E-64	81.00%
1191	Cl_clus101220_Contig1	---NA---	324	0 -	-	
1192	Cl_clus101221_Contig1	drosophila melanogaster chd64 transcript variant mrna	567	14	1.00E-82	72.93%
1193	Cl_clus101222_Contig1	nematostella vectensis protein partial mrna	492	1	3.80E-43	75.00%
1194	Cl_clus101223_Contig1	---NA---	797	0 -	-	
1195	Cl_clus101224_Contig1	---NA---	421	0 -	-	
1196	Cl_clus101225_Contig1	---NA---	263	0 -	-	
1197	Cl_clus101227_Contig1	---NA---	350	0 -	-	
1198	Cl_clus101228_Contig1	---NA---	778	0 -	-	
1199	Cl_clus101229_Contig1	---NA---	353	0 -	-	

1200	Cl_clus101231_Contig1	---NA---	457	0 -	-	
1201	Cl_clus101226_Contig1	---NA---	488	0 -	-	
1202	Cl_clus101232_Contig1	apostichopus japonicus catalase complete cds	394	3	3.00E-81	75.00%
1203	Cl_clus101233_Contig1	anopheles gambiae pest agap010750-pa complete cds	508	2	1.40E-23	83.00%
1204	Cl_clus101234_Contig1	---NA---	487	0 -	-	
1205	Cl_clus101230_Contig1	---NA---	825	0 -	-	
1206	Cl_clus101235_Contig1	---NA---	414	0 -	-	
1207	Cl_clus101237_Contig1	---NA---	332	0 -	-	
1208	Cl_clus101238_Contig1	single read from an extremity of a full-length cdna clone made from anopheles gambiae total adult 5-prime end of clone fk0aac35ac08 of strain 6-9 of anopheles gambiae (african malaria mosquito)	633	20	2.20E-66	72.45%
1209	Cl_clus101239_Contig1	---NA---	241	0 -	-	
1210	Cl_clus101240_Contig1	---NA---	398	0 -	-	
1211	Cl_clus101241_Contig1	---NA---	421	0 -	-	
1212	Cl_clus101242_Contig1	---NA---	518	0 -	-	
1213	Cl_clus101246_Contig1	---NA---	329	0 -	-	
1214	Cl_clus101236_Contig1	---NA---	373	0 -	-	
1215	Cl_clus101243_Contig1	---NA---	438	0 -	-	
1216	Cl_clus101247_Contig1	---NA---	399	0 -	-	
1217	Cl_clus101244_Contig1	---NA---	396	0 -	-	
1218	Cl_clus101248_Contig1	---NA---	556	0 -	-	
1219	Cl_clus101245_Contig1	---NA---	663	0 -	-	
1220	Cl_clus101249_Contig1	---NA---	460	0 -	-	
1221	Cl_clus101252_Contig1	---NA---	360	0 -	-	
1222	Cl_clus101251_Contig1	---NA---	319	0 -	-	
1223	Cl_clus101254_Contig1	---NA---	246	0 -	-	
1224	Cl_clus101255_Contig1	---NA---	523	0 -	-	
1225	Cl_clus101256_Contig1	---NA---	336	0 -	-	
1226	Cl_clus101257_Contig1	---NA---	819	0 -	-	
1227	Cl_clus101258_Contig1	---NA---	420	0 -	-	
1228	Cl_clus101253_Contig1	---NA---	317	0 -	-	
1229	Cl_clus101259_Contig1	---NA---	537	0 -	-	
1230	Cl_clus101260_Contig1	---NA---	618	0 -	-	
1231	Cl_clus101261_Contig1	---NA---	495	0 -	-	
1232	Cl_clus101262_Contig1	gadus morhua sec61 gamma subunit complete cds	452	1	1.40E-35	77.00%
1233	Cl_clus101263_Contig1	---NA---	312	0 -	-	
1234	Cl_clus101264_Contig1	---NA---	658	0 -	-	
1235	Cl_clus101265_Contig1	---NA---	554	0 -	-	
1236	Cl_clus101267_Contig1	---NA---	224	0 -	-	
1237	Cl_clus101268_Contig1	acyrthosiphon pisum eukaryotic translation initiation factor 3 subunit e-like mrna	327	2	4.30E-59	75.00%
1238	Cl_clus101269_Contig1	---NA---	311	0 -	-	
1239	Cl_clus101270_Contig1	---NA---	268	0 -	-	
1240	Cl_clus101271_Contig1	---NA---	207	0 -	-	
1241	Cl_clus101272_Contig1	---NA---	312	0 -	-	
1242	Cl_clus101275_Contig1	---NA---	404	0 -	-	
1243	Cl_clus101276_Contig1	---NA---	285	0 -	-	
1244	Cl_clus101277_Contig1	---NA---	427	0 -	-	
1245	Cl_clus101278_Contig1	---NA---	551	0 -	-	
1246	Cl_clus101279_Contig1	---NA---	529	0 -	-	
1247	Cl_clus101280_Contig1	---NA---	294	0 -	-	
1248	Cl_clus101273_Contig1	---NA---	575	0 -	-	
1249	Cl_clus101274_Contig1	---NA---	577	0 -	-	
1250	Cl_clus101266_Contig1	---NA---	325	0 -	-	
1251	Cl_clus101281_Contig1	---NA---	365	0 -	-	
1252	Cl_clus101282_Contig1	---NA---	575	0 -	-	
1253	Cl_clus101283_Contig1	---NA---	345	0 -	-	
1254	Cl_clus101284_Contig1	---NA---	485	0 -	-	
1255	Cl_clus101285_Contig1	---NA---	337	0 -	-	
1256	Cl_clus101286_Contig1	---NA---	364	0 -	-	

1257	Cl_clus101287_Contig1	---NA---	499	0 -	-	
1258	Cl_clus101288_Contig1	---NA---	453	0 -	-	
1259	Cl_clus101289_Contig1	drosophila melanogaster ribosomal protein s4 transcript variant mrna	442	17	4.30E-105	78.00%
1260	Cl_clus101290_Contig1	---NA---	520	0 -	-	
1261	Cl_clus101291_Contig1	---NA---	478	0 -	-	
1262	Cl_clus101293_Contig1	drosophila melanogaster knockdown transcript variant mrna	527	13	2.70E-64	75.15%
1263	Cl_clus101294_Contig1	---NA---	299	0 -	-	
1264	Cl_clus101295_Contig1	---NA---	509	0 -	-	
1265	Cl_clus101296_Contig1	---NA---	447	0 -	-	
1266	Cl_clus101297_Contig1	---NA---	497	0 -	-	
1267	Cl_clus101298_Contig1	---NA---	363	0 -	-	
1268	Cl_clus101292_Contig1	---NA---	547	0 -	-	
1269	Cl_clus101299_Contig1	hyriopsis cumingii 37 kda laminin receptor complete cds	605	1	1.90E-73	74.00%
1270	Cl_clus101300_Contig1	---NA---	429	0 -	-	
1271	Cl_clus101301_Contig1	---NA---	394	0 -	-	
1272	Cl_clus101302_Contig1	---NA---	629	0 -	-	
1273	Cl_clus101304_Contig1	---NA---	409	0 -	-	
1274	Cl_clus101305_Contig1	---NA---	362	0 -	-	
1275	Cl_clus101303_Contig1	---NA---	324	0 -	-	
1276	Cl_clus101306_Contig1	---NA---	274	0 -	-	
1277	Cl_clus101309_Contig1	---NA---	471	0 -	-	
1278	Cl_clus101311_Contig1	---NA---	427	0 -	-	
1279	Cl_clus101312_Contig1	---NA---	2111	0 -	-	
1280	Cl_clus101307_Contig1	plutella xylostella 40 protein complete cds	538	20	9.70E-102	79.85%
1281	Cl_clus101310_Contig1	---NA---	288	0 -	-	
1282	Cl_clus101313_Contig1	---NA---	571	0 -	-	
1283	Cl_clus101314_Contig1	---NA---	305	0 -	-	
1284	Cl_clus101308_Contig1	nematostella vectensis protein partial mrna	476	3	3.90E-68	74.00%
1285	Cl_clus101315_Contig1	---NA---	682	0 -	-	
1286	Cl_clus101316_Contig1	---NA---	291	0 -	-	
1287	Cl_clus101317_Contig1	---NA---	268	0 -	-	
1288	Cl_clus101318_Contig1	---NA---	460	0 -	-	
1289	Cl_clus101319_Contig1	---NA---	272	0 -	-	
1290	Cl_clus101320_Contig1	---NA---	266	0 -	-	
1291	Cl_clus101321_Contig1	---NA---	363	0 -	-	
1292	Cl_clus101323_Contig1	---NA---	239	0 -	-	
1293	Cl_clus101325_Contig1	---NA---	656	0 -	-	
1294	Cl_clus101326_Contig1	---NA---	294	0 -	-	
1295	Cl_clus101322_Contig1	---NA---	784	0 -	-	
1296	Cl_clus101327_Contig1	---NA---	426	0 -	-	
1297	Cl_clus101329_Contig1	---NA---	288	0 -	-	
1298	Cl_clus101324_Contig1	---NA---	430	0 -	-	
1299	Cl_clus101330_Contig1	---NA---	432	0 -	-	
1300	Cl_clus101328_Contig1	---NA---	374	0 -	-	
1301	Cl_clus101331_Contig1	---NA---	331	0 -	-	
1302	Cl_clus101332_Contig1	---NA---	215	0 -	-	
1303	Cl_clus101333_Contig1	---NA---	382	0 -	-	
1304	Cl_clus101334_Contig1	---NA---	264	0 -	-	
1305	Cl_clus101335_Contig1	---NA---	325	0 -	-	
1306	Cl_clus101338_Contig1	---NA---	497	0 -	-	
1307	Cl_clus101339_Contig1	---NA---	368	0 -	-	
1308	Cl_clus101340_Contig1	---NA---	671	0 -	-	
1309	Cl_clus101337_Contig1	---NA---	307	0 -	-	
1310	Cl_clus101341_Contig1	---NA---	291	0 -	-	
1311	Cl_clus101342_Contig1	---NA---	484	0 -	-	
1312	Cl_clus101343_Contig1	---NA---	223	0 -	-	
1313	Cl_clus101344_Contig1	---NA---	320	0 -	-	
1314	Cl_clus101345_Contig1	---NA---	574	0 -	-	
1315	Cl_clus101346_Contig1	---NA---	329	0 -	-	
1316	Cl_clus101347_Contig1	---NA---	355	0 -	-	

1317	Cl_clus101348_Contig1	---NA---	391	0 -	-	
1318	Cl_clus101349_Contig1	---NA---	540	0 -	-	
1319	Cl_clus101350_Contig1	---NA---	354	0 -	-	
1320	Cl_clus101336_Contig1	ixodes scapularis protein phosphatase 2a regulatory subunit mrna	482	1	2.00E-65	72.00%
1321	Cl_clus101352_Contig1	---NA---	290	0 -	-	
1322	Cl_clus101351_Contig1	ciona intestinalis gamma-aminobutyric acid receptor-associated mrna	1023	3	1.50E-20	82.33%
1323	Cl_clus101353_Contig1	---NA---	599	0 -	-	
1324	Cl_clus101354_Contig1	---NA---	720	0 -	-	
1325	Cl_clus101355_Contig1	---NA---	312	0 -	-	
1326	Cl_clus101357_Contig1	---NA---	425	0 -	-	
1327	Cl_clus101358_Contig1	---NA---	921	0 -	-	
1328	Cl_clus101356_Contig1	---NA---	318	0 -	-	
1329	Cl_clus101359_Contig1	---NA---	428	0 -	-	
1330	Cl_clus101360_Contig1	---NA---	285	0 -	-	
1331	Cl_clus101361_Contig1	---NA---	349	0 -	-	
1332	Cl_clus101362_Contig1	---NA---	690	0 -	-	
1333	Cl_clus101363_Contig1	---NA---	205	0 -	-	
1334	Cl_clus101364_Contig1	---NA---	253	0 -	-	
1335	Cl_clus101365_Contig1	---NA---	242	0 -	-	
1336	Cl_clus101367_Contig1	---NA---	207	0 -	-	
1337	Cl_clus101368_Contig1	---NA---	281	0 -	-	
1338	Cl_clus101366_Contig1	---NA---	331	0 -	-	
1339	Cl_clus101370_Contig1	---NA---	366	0 -	-	
1340	Cl_clus101369_Contig1	---NA---	611	0 -	-	
1341	Cl_clus101372_Contig1	---NA---	289	0 -	-	
1342	Cl_clus101373_Contig1	---NA---	309	0 -	-	
1343	Cl_clus101374_Contig1	---NA---	481	0 -	-	
1344	Cl_clus101375_Contig1	---NA---	259	0 -	-	
1345	Cl_clus101371_Contig1	---NA---	370	0 -	-	
1346	Cl_clus101376_Contig1	---NA---	371	0 -	-	
1347	Cl_clus101377_Contig1	---NA---	719	0 -	-	
1348	Cl_clus101378_Contig1	ixodes pacificus clone ip_7_60_92_99_clu 40s ribosomal protein s30 complete cds	510	2	2.50E-39	82.00%
1349	Cl_clus101379_Contig1	---NA---	517	0 -	-	
1350	Cl_clus101380_Contig1	drosophila melanogaster gasp transcript variant mrna	349	15	8.40E-75	78.20%
1351	Cl_clus101381_Contig1	---NA---	584	0 -	-	
1352	Cl_clus101382_Contig1	---NA---	593	0 -	-	
1353	Cl_clus101384_Contig1	---NA---	368	0 -	-	
1354	Cl_clus101385_Contig1	---NA---	229	0 -	-	
1355	Cl_clus101383_Contig1	---NA---	376	0 -	-	
1356	Cl_clus101386_Contig1	---NA---	353	0 -	-	
1357	Cl_clus101387_Contig1	---NA---	607	0 -	-	
1358	Cl_clus101389_Contig1	---NA---	269	0 -	-	
1359	Cl_clus101390_Contig1	gryllus bimaculatus gbcontig29054	448	3	3.60E-68	77.67%
1360	Cl_clus101391_Contig1	---NA---	449	0 -	-	
1361	Cl_clus101392_Contig1	---NA---	266	0 -	-	
1362	Cl_clus101393_Contig1	---NA---	225	0 -	-	
1363	Cl_clus101394_Contig1	---NA---	658	0 -	-	
1364	Cl_clus101395_Contig1	---NA---	266	0 -	-	
1365	Cl_clus101388_Contig1	---NA---	862	0 -	-	
1366	Cl_clus101396_Contig1	tribolium castaneum protein mrna	310	1	4.40E-65	78.00%
1367	Cl_clus101397_Contig1	---NA---	467	0 -	-	
1368	Cl_clus101398_Contig1	---NA---	268	0 -	-	
1369	Cl_clus101399_Contig1	---NA---	287	0 -	-	
1370	Cl_clus101400_Contig1	---NA---	544	0 -	-	
1371	Cl_clus101401_Contig1	---NA---	597	0 -	-	
1372	Cl_clus101402_Contig1	---NA---	372	0 -	-	
1373	Cl_clus101407_Contig1	---NA---	453	0 -	-	
1374	Cl_clus101404_Contig1	---NA---	243	0 -	-	
1375	Cl_clus101408_Contig1	---NA---	314	0 -	-	

1376	Cl_clus101409_Contig1	---NA---	429	0	-	-
1377	Cl_clus101410_Contig1	---NA---	617	0	-	-
1378	Cl_clus101411_Contig1	---NA---	297	0	-	-
1379	Cl_clus101405_Contig1	setaria italica iron-sulfur cluster assembly protein 1-like mrna	356	11	5.50E-33	73.00%
1380	Cl_clus101412_Contig1	---NA---	623	0	-	-
1381	Cl_clus101403_Contig1	---NA---	388	0	-	-
1382	Cl_clus101414_Contig1	---NA---	432	0	-	-
1383	Cl_clus101415_Contig1	---NA---	425	0	-	-
1384	Cl_clus101406_Contig1	---NA---	603	0	-	-
1385	Cl_clus101416_Contig1	---NA---	202	0	-	-
1386	Cl_clus101417_Contig1	---NA---	436	0	-	-
1387	Cl_clus101418_Contig1	---NA---	288	0	-	-
1388	Cl_clus101413_Contig1	megachile rotundata ryanodine receptor 44f-like mrna	471	1	2.00E-84	75.00%
1389	Cl_clus101419_Contig1	---NA---	428	0	-	-
1390	Cl_clus101420_Contig1	---NA---	441	0	-	-
1391	Cl_clus101421_Contig1	---NA---	290	0	-	-
1392	Cl_clus101423_Contig1	---NA---	281	0	-	-
1393	Cl_clus101424_Contig1	---NA---	551	0	-	-
1394	Cl_clus101425_Contig1	---NA---	685	0	-	-
1395	Cl_clus101426_Contig1	---NA---	780	0	-	-
1396	Cl_clus101422_Contig1	---NA---	710	0	-	-
1397	Cl_clus101427_Contig1	drosophila pseudoobscura pseudoobscura ga10697 (dpse\ga10697) mrna	335	2	5.10E-52	83.00%
1398	Cl_clus101428_Contig1	---NA---	323	0	-	-
1399	Cl_clus101429_Contig1	---NA---	219	0	-	-
1400	Cl_clus101431_Contig1	---NA---	225	0	-	-
1401	Cl_clus101432_Contig1	---NA---	312	0	-	-
1402	Cl_clus101433_Contig1	---NA---	421	0	-	-
1403	Cl_clus101430_Contig1	---NA---	457	0	-	-
1404	Cl_clus101434_Contig1	bos taurus peroxiredoxin 5 mrna	450	2	3.00E-12	78.00%
1405	Cl_clus101435_Contig1	---NA---	259	0	-	-
1406	Cl_clus101437_Contig1	---NA---	370	0	-	-
1407	Cl_clus101439_Contig1	---NA---	345	0	-	-
1408	Cl_clus101440_Contig1	---NA---	456	0	-	-
1409	Cl_clus101436_Contig1	---NA---	537	0	-	-
1410	Cl_clus101438_Contig1	---NA---	377	0	-	-
1411	Cl_clus101441_Contig1	---NA---	424	0	-	-
1412	Cl_clus101442_Contig1	---NA---	633	0	-	-
1413	Cl_clus101443_Contig1	---NA---	267	0	-	-
1414	Cl_clus101444_Contig1	---NA---	348	0	-	-
1415	Cl_clus101447_Contig1	---NA---	615	0	-	-
1416	Cl_clus101448_Contig1	---NA---	487	0	-	-
1417	Cl_clus101449_Contig1	---NA---	380	0	-	-
1418	Cl_clus101450_Contig1	---NA---	369	0	-	-
1419	Cl_clus101445_Contig1	---NA---	361	0	-	-
1420	Cl_clus101451_Contig1	---NA---	283	0	-	-
1421	Cl_clus101446_Contig1	---NA---	453	0	-	-
1422	Cl_clus101453_Contig1	---NA---	929	0	-	-
1423	Cl_clus101454_Contig1	---NA---	416	0	-	-
1424	Cl_clus101455_Contig1	---NA---	455	0	-	-
1425	Cl_clus101456_Contig1	---NA---	288	0	-	-
1426	Cl_clus101460_Contig1	---NA---	233	0	-	-
1427	Cl_clus101459_Contig1	---NA---	294	0	-	-
1428	Cl_clus101461_Contig1	---NA---	221	0	-	-
1429	Cl_clus101463_Contig1	---NA---	537	0	-	-
1430	Cl_clus101462_Contig1	---NA---	579	0	-	-
1431	Cl_clus101465_Contig1	---NA---	348	0	-	-
1432	Cl_clus101464_Contig1	---NA---	376	0	-	-
1433	Cl_clus101457_Contig1	---NA---	561	0	-	-
1434	Cl_clus101458_Contig1	platynereis dumerilii est ib0aaa41ab04em1	445	8	8.50E-89	79.88%

1435	Cl_clus101466_Contig1	---NA---	356	0 -	-	
1436	Cl_clus101468_Contig1	---NA---	339	0 -	-	
1437	Cl_clus101467_Contig1	---NA---	441	0 -	-	
1438	Cl_clus101469_Contig1	---NA---	307	0 -	-	
1439	Cl_clus101470_Contig1	---NA---	520	0 -	-	
1440	Cl_clus101471_Contig1	---NA---	461	0 -	-	
1441	Cl_clus101472_Contig1	---NA---	223	0 -	-	
1442	Cl_clus101473_Contig1	plutella xylostella ryanodine receptor complete cds	820	16	0.00E+00	80.94%
1443	Cl_clus101474_Contig1	---NA---	272	0 -	-	
1444	Cl_clus101475_Contig1	---NA---	554	0 -	-	
1445	Cl_clus101476_Contig1	strongylocentrotus purpuratus nascent polypeptide-associated complex subunit alpha-like mrna	557	1	5.60E-29	76.00%
1446	Cl_clus101477_Contig1	---NA---	371	0 -	-	
1447	Cl_clus101478_Contig1	---NA---	355	0 -	-	
1448	Cl_clus101479_Contig1	---NA---	453	0 -	-	
1449	Cl_clus101481_Contig1	---NA---	415	0 -	-	
1450	Cl_clus101483_Contig1	---NA---	233	0 -	-	
1451	Cl_clus101484_Contig1	---NA---	368	0 -	-	
1452	Cl_clus101480_Contig1	---NA---	645	0 -	-	
1453	Cl_clus101486_Contig1	---NA---	230	0 -	-	
1454	Cl_clus101487_Contig1	---NA---	451	0 -	-	
1455	Cl_clus101482_Contig1	---NA---	598	0 -	-	
1456	Cl_clus101485_Contig1	---NA---	264	0 -	-	
1457	Cl_clus101488_Contig2	nasonia vitripennis transmembrane protein 35-like mrna	646	1	4.10E-25	67.00%
1458	Cl_clus101489_Contig1	---NA---	307	0 -	-	
1459	Cl_clus101490_Contig1	---NA---	292	0 -	-	
1460	Cl_clus101491_Contig1	---NA---	285	0 -	-	
1461	Cl_clus101492_Contig1	---NA---	374	0 -	-	
1462	Cl_clus101493_Contig1	---NA---	344	0 -	-	
1463	Cl_clus101494_Contig1	---NA---	600	0 -	-	
1464	Cl_clus101495_Contig1	---NA---	523	0 -	-	
1465	Cl_clus101496_Contig1	---NA---	381	0 -	-	
1466	Cl_clus101497_Contig1	---NA---	405	0 -	-	
1467	Cl_clus101498_Contig1	---NA---	353	0 -	-	
1468	Cl_clus101499_Contig1	---NA---	834	0 -	-	
1469	Cl_clus101500_Contig1	---NA---	339	0 -	-	
1470	Cl_clus101501_Contig1	---NA---	655	0 -	-	
1471	Cl_clus101502_Contig1	maylandia zebra ras-related protein rab-1a-like transcript variant mrna	753	7	1.00E-90	77.29%
1472	Cl_clus101504_Contig1	---NA---	298	0 -	-	
1473	Cl_clus101505_Contig1	---NA---	452	0 -	-	
1474	Cl_clus101506_Contig1	---NA---	398	0 -	-	
1475	Cl_clus101507_Contig1	---NA---	581	0 -	-	
1476	Cl_clus101508_Contig1	---NA---	435	0 -	-	
1477	Cl_clus101509_Contig1	anopheles gambiae pest agap005960-pa complete cds	318	3	9.30E-36	74.33%
1478	Cl_clus101503_Contig1	---NA---	294	0 -	-	
1479	Cl_clus101510_Contig1	---NA---	499	0 -	-	
1480	Cl_clus101511_Contig1	---NA---	500	0 -	-	
1481	Cl_clus101512_Contig1	---NA---	653	0 -	-	
1482	Cl_clus101513_Contig1	---NA---	534	0 -	-	
1483	Cl_clus101514_Contig1	---NA---	496	0 -	-	
1484	Cl_clus101515_Contig1	---NA---	328	0 -	-	
1485	Cl_clus101516_Contig1	---NA---	370	0 -	-	
1486	Cl_clus101517_Contig1	---NA---	580	0 -	-	
1487	Cl_clus101518_Contig1	rana catesbeiana clone rcat-evr-518-049 40s ribosomal protein s16 complete cds	373	4	7.50E-76	77.50%
1488	Cl_clus101519_Contig1	---NA---	305	0 -	-	
1489	Cl_clus101520_Contig1	---NA---	297	0 -	-	
1490	Cl_clus101521_Contig1	---NA---	453	0 -	-	
1491	Cl_clus101522_Contig1	---NA---	402	0 -	-	
1492	Cl_clus101523_Contig1	---NA---	319	0 -	-	

1493	Cl_clus101525_Contig1	---NA---	355	0 -	-	
1494	Cl_clus101526_Contig1	tetraodon nigroviridis full-length cdna	1012	20	1.50E-159	74.15%
1495	Cl_clus101527_Contig1	---NA---	608	0 -	-	
1496	Cl_clus101529_Contig1	---NA---	231	0 -	-	
1497	Cl_clus101530_Contig1	---NA---	363	0 -	-	
1498	Cl_clus101531_Contig1	gryllus bimaculatus gbcontig28025	416	20	1.20E-149	78.05%
1499	Cl_clus101532_Contig1	---NA---	495	0 -	-	
1500	Cl_clus101533_Contig1	---NA---	782	0 -	-	
1501	Cl_clus101524_Contig1	---NA---	556	0 -	-	
1502	Cl_clus101528_Contig1	---NA---	240	0 -	-	
1503	Cl_clus101534_Contig1	---NA---	201	0 -	-	
1504	Cl_clus101535_Contig1	---NA---	393	0 -	-	
1505	Cl_clus101536_Contig1	---NA---	339	0 -	-	
1506	Cl_clus101537_Contig1	---NA---	314	0 -	-	
1507	Cl_clus101538_Contig1	---NA---	537	0 -	-	
1508	Cl_clus101539_Contig1	---NA---	728	0 -	-	
1509	Cl_clus101540_Contig1	---NA---	517	0 -	-	
1510	Cl_clus101541_Contig1	---NA---	495	0 -	-	
1511	Cl_clus101542_Contig1	---NA---	337	0 -	-	
1512	Cl_clus101543_Contig1	---NA---	363	0 -	-	
1513	Cl_clus101544_Contig1	---NA---	662	0 -	-	
1514	Cl_clus101545_Contig1	---NA---	872	0 -	-	
1515	Cl_clus101546_Contig1	---NA---	322	0 -	-	
1516	Cl_clus101547_Contig1	---NA---	275	0 -	-	
1517	Cl_clus101548_Contig1	---NA---	639	0 -	-	
1518	Cl_clus101549_Contig1	---NA---	244	0 -	-	
1519	Cl_clus101550_Contig1	---NA---	403	0 -	-	
1520	Cl_clus101551_Contig1	---NA---	457	0 -	-	
1521	Cl_clus101552_Contig1	---NA---	362	0 -	-	
1522	Cl_clus101553_Contig1	---NA---	456	0 -	-	

1523	Cl_clus101554_Contig1	---NA---	238	0 -	-	
1524	Cl_clus101555_Contig1	---NA---	283	0 -	-	
1525	Cl_clus101557_Contig1	---NA---	411	0 -	-	
1526	Cl_clus101558_Contig1	nasonia vitripennis elongation factor 2-like mrna	508	6	4.10E-87	77.17%
1527	Cl_clus101559_Contig1	---NA---	380	0 -	-	
1528	Cl_clus101561_Contig1	arabidopsis thaliana at1g67090 complete cds	470	20	0.00E+00	97.00%
1529	Cl_clus101562_Contig1	---NA---	662	0 -	-	
1530	Cl_clus101563_Contig1	---NA---	968	0 -	-	
1531	Cl_clus101564_Contig1	---NA---	322	0 -	-	
1532	Cl_clus101556_Contig1	sipyloidea sipylus mitochondrial partial genome	231	20	5.60E-62	80.70%
1533	Cl_clus101560_Contig1	arabidopsis thaliana at1g29930 f1n18_23 complete cds	590	20	0.00E+00	93.30%
1534	Cl_clus101565_Contig1	---NA---	419	0 -	-	
1535	Cl_clus101566_Contig1	---NA---	359	0 -	-	
1536	Cl_clus101567_Contig1	---NA---	513	0 -	-	
1537	Cl_clus101568_Contig1	papilio polytes mrna for troponin complete sequence id: pp-0016	366	20	1.70E-39	84.70%
1538	Cl_clus101569_Contig1	drosophila melanogaster myosin heavy chain transcript variant mrna	596	20	0.00E+00	80.25%
1539	Cl_clus101570_Contig1	---NA---	285	0 -	-	
1540	Cl_clus101573_Contig2	---NA---	293	0 -	-	
1541	Cl_clus101575_Contig1	---NA---	439	0 -	-	
1542	Cl_clus101576_Contig1	---NA---	314	0 -	-	
1543	Cl_clus101577_Contig1	---NA---	247	0 -	-	
1544	Cl_clus101578_Contig1	---NA---	333	0 -	-	
1545	Cl_clus101571_Contig1	bombyx mori myosin light chain 2 complete cds	990	20	4.30E-160	79.00%
1546	Cl_clus101572_Contig1	---NA---	774	0 -	-	
1547	Cl_clus101579_Contig1	---NA---	218	0 -	-	
1548	Cl_clus101580_Contig1	---NA---	223	0 -	-	
1549	Cl_clus101581_Contig1	---NA---	268	0 -	-	
1550	Cl_clus101582_Contig1	---NA---	293	0 -	-	
1551	Cl_clus101583_Contig1	---NA---	335	0 -	-	
1552	Cl_clus101585_Contig1	salmo salar 6-phosphofructo-2-kinase fructose- -biphosphatase 2 mrna	476	1	3.20E-12	80.00%

1553	Cl_clus101586_Contig1	---NA---	272	0 -	-	
1554	Cl_clus101587_Contig1	---NA---	357	0 -	-	
1555	Cl_clus101584_Contig1	---NA---	234	0 -	-	
1556	Cl_clus101589_Contig1	---NA---	350	0 -	-	
1557	Cl_clus101590_Contig1	---NA---	202	0 -	-	
1558	Cl_clus101592_Contig1	---NA---	325	0 -	-	
1559	Cl_clus101593_Contig1	---NA---	423	0 -	-	
1560	Cl_clus101588_Contig1	---NA---	261	0 -	-	
1561	Cl_clus101594_Contig1	---NA---	249	0 -	-	
1562	Cl_clus101595_Contig1	---NA---	250	0 -	-	
1563	Cl_clus101596_Contig1	---NA---	328	0 -	-	
1564	Cl_clus101597_Contig1	---NA---	297	0 -	-	
1565	Cl_clus101598_Contig1	---NA---	235	0 -	-	
1566	Cl_clus101599_Contig1	---NA---	310	0 -	-	
1567	Cl_clus101600_Contig1	---NA---	259	0 -	-	
1568	Cl_clus101601_Contig1	---NA---	308	0 -	-	
1569	Cl_clus101602_Contig1	---NA---	340	0 -	-	
1570	Cl_clus101604_Contig1	---NA---	322	0 -	-	
1571	Cl_clus101608_Contig1	---NA---	332	0 -	-	
1572	Cl_clus101609_Contig1	---NA---	287	0 -	-	
1573	Cl_clus101605_Contig1	---NA---	239	0 -	-	
1574	Cl_clus101606_Contig1	---NA---	353	0 -	-	
1575	Cl_clus101610_Contig1	---NA---	376	0 -	-	
1576	Cl_clus101613_Contig1	---NA---	212	0 -	-	
1577	Cl_clus101614_Contig1	---NA---	458	0 -	-	
1578	Cl_clus101616_Contig1	---NA---	514	0 -	-	
1579	Cl_clus101618_Contig1	ovis aries iron-sulfur cluster assembly 1 mitochondrial-like mrna	598	14	2.60E-27	80.86%
1580	Cl_clus101619_Contig1	---NA---	281	0 -	-	
1581	Cl_clus101620_Contig1	---NA---	222	0 -	-	
1582	Cl_clus101621_Contig1	---NA---	391	0 -	-	
1583	Cl_clus101623_Contig1	---NA---	355	0 -	-	
1584	Cl_clus101617_Contig1	---NA---	205	0 -	-	
1585	Cl_clus101615_Contig1	---NA---	235	0 -	-	
1586	Cl_clus101622_Contig1	---NA---	262	0 -	-	
1587	Cl_clus101624_Contig1	---NA---	626	0 -	-	
1588	Cl_clus101625_Contig1	---NA---	356	0 -	-	
1589	Cl_clus101626_Contig1	---NA---	416	0 -	-	
1590	Cl_clus101627_Contig1	---NA---	380	0 -	-	
1591	Cl_clus101629_Contig1	---NA---	234	0 -	-	
1592	Cl_clus101630_Contig1	---NA---	329	0 -	-	
1593	Cl_clus101631_Contig1	---NA---	344	0 -	-	
1594	Cl_clus101632_Contig1	---NA---	297	0 -	-	
1595	Cl_clus101634_Contig1	---NA---	347	0 -	-	
1596	Cl_clus101636_Contig1	---NA---	838	0 -	-	
1597	Cl_clus101633_Contig1	---NA---	424	0 -	-	
1598	Cl_clus101635_Contig1	---NA---	393	0 -	-	
1599	Cl_clus101639_Contig1	---NA---	204	0 -	-	
1600	Cl_clus101637_Contig1	---NA---	201	0 -	-	
1601	Cl_clus101638_Contig1	---NA---	277	0 -	-	
1602	Cl_clus101642_Contig1	---NA---	451	0 -	-	
1603	Cl_clus101643_Contig1	---NA---	365	0 -	-	
1604	Cl_clus101644_Contig1	---NA---	351	0 -	-	
1605	Cl_clus101645_Contig1	---NA---	244	0 -	-	
1606	Cl_clus101648_Contig1	---NA---	309	0 -	-	
1607	Cl_clus101649_Contig1	---NA---	229	0 -	-	
1608	Cl_clus101640_Contig1	---NA---	232	0 -	-	
1609	Cl_clus101650_Contig1	---NA---	341	0 -	-	
1610	Cl_clus101652_Contig1	---NA---	281	0 -	-	
1611	Cl_clus101653_Contig1	---NA---	305	0 -	-	
1612	Cl_clus101654_Contig1	---NA---	275	0 -	-	

1613	Cl_clus101647_Contig1	---NA---	263	0 -	-	
1614	Cl_clus101655_Contig1	---NA---	304	0 -	-	
1615	Cl_clus101658_Contig1	---NA---	359	0 -	-	
1616	Cl_clus101660_Contig1	---NA---	236	0 -	-	
1617	Cl_clus101661_Contig1	---NA---	224	0 -	-	
1618	Cl_clus101657_Contig1	---NA---	295	0 -	-	
1619	Cl_clus101663_Contig1	---NA---	266	0 -	-	
1620	Cl_clus101664_Contig1	---NA---	218	0 -	-	
1621	Cl_clus101659_Contig1	---NA---	319	0 -	-	
1622	Cl_clus101666_Contig1	culex quinquefasciatus ryanodine mrna	471	4	3.10E-107	76.50%
1623	Cl_clus101667_Contig1	---NA---	295	0 -	-	
1624	Cl_clus101665_Contig1	---NA---	369	0 -	-	
1625	Cl_clus101668_Contig1	---NA---	271	0 -	-	
1626	Cl_clus101670_Contig1	---NA---	243	0 -	-	
1627	Cl_clus101671_Contig1	---NA---	378	0 -	-	
1628	Cl_clus101662_Contig1	---NA---	313	0 -	-	
1629	Cl_clus101673_Contig1	---NA---	223	0 -	-	
1630	Cl_clus101669_Contig1	acyrthosiphon pisum chloride intracellular channel exc-4-like mrna	288	1	2.20E-11	91.00%
1631	Cl_clus101674_Contig1	---NA---	215	0 -	-	
1632	Cl_clus101675_Contig1	---NA---	203	0 -	-	
1633	Cl_clus101676_Contig1	---NA---	389	0 -	-	
1634	Cl_clus101678_Contig1	---NA---	226	0 -	-	
1635	Cl_clus101677_Contig1	---NA---	237	0 -	-	
1636	Cl_clus101679_Contig1	---NA---	257	0 -	-	
1637	Cl_clus101680_Contig1	---NA---	308	0 -	-	
1638	Cl_clus101681_Contig1	---NA---	393	0 -	-	
1639	Cl_clus101682_Contig1	---NA---	210	0 -	-	
1640	Cl_clus101683_Contig1	---NA---	273	0 -	-	
1641	Cl_clus101684_Contig1	---NA---	350	0 -	-	
1642	Cl_clus101686_Contig1	---NA---	231	0 -	-	
1643	Cl_clus101688_Contig1	---NA---	258	0 -	-	
1644	Cl_clus101689_Contig1	---NA---	340	0 -	-	
1645	Cl_clus101690_Contig1	---NA---	246	0 -	-	
1646	Cl_clus101692_Contig1	---NA---	397	0 -	-	
1647	Cl_clus101694_Contig1	---NA---	533	0 -	-	
1648	Cl_clus101696_Contig1	---NA---	323	0 -	-	
1649	Cl_clus101698_Contig1	---NA---	438	0 -	-	
1650	Cl_clus101699_Contig1	---NA---	332	0 -	-	
1651	Cl_clus101700_Contig1	---NA---	231	0 -	-	
1652	Cl_clus101701_Contig1	---NA---	438	0 -	-	
1653	Cl_clus101704_Contig1	---NA---	234	0 -	-	
1654	Cl_clus101705_Contig1	---NA---	203	0 -	-	
1655	Cl_clus101697_Contig1	---NA---	336	0 -	-	
1656	Cl_clus101706_Contig1	---NA---	290	0 -	-	
1657	Cl_clus101708_Contig1	---NA---	345	0 -	-	
1658	Cl_clus101710_Contig1	---NA---	358	0 -	-	
1659	Cl_clus101709_Contig1	---NA---	203	0 -	-	
1660	Cl_clus101711_Contig1	---NA---	286	0 -	-	
1661	Cl_clus101713_Contig1	---NA---	301	0 -	-	
1662	Cl_clus101712_Contig1	---NA---	369	0 -	-	
1663	Cl_clus101714_Contig1	---NA---	209	0 -	-	
1664	Cl_clus101717_Contig1	---NA---	250	0 -	-	
1665	Cl_clus101720_Contig1	---NA---	266	0 -	-	
1666	Cl_clus101721_Contig1	---NA---	228	0 -	-	
1667	Cl_clus101722_Contig1	---NA---	495	0 -	-	
1668	Cl_clus101723_Contig1	---NA---	399	0 -	-	
1669	Cl_clus101724_Contig1	---NA---	254	0 -	-	
1670	Cl_clus101726_Contig1	---NA---	228	0 -	-	
1671	Cl_clus101727_Contig1	ficedula albicollis tubulin alpha-1c chain-like transcript variant mrna	280	20	8.60E-118	92.10%
1672	Cl_clus101728_Contig1	---NA---	203	0 -	-	

1673	Cl_clus101730_Contig1	---NA---	383	0 -	-	
1674	Cl_clus101732_Contig1	aedes aegypti protein disulfide isomerase partial mrna	237	4	3.00E-21	75.00%
1675	Cl_clus101733_Contig1	---NA---	276	0 -	-	
1676	Cl_clus101734_Contig1	octodon degus ca++ plasma membrane 4 transcript variant mrna	397	20	7.50E-32	76.70%
1677	Cl_clus101736_Contig1	---NA---	302	0 -	-	
1678	Cl_clus101737_Contig1	---NA---	267	0 -	-	
1679	Cl_clus101740_Contig1	---NA---	411	0 -	-	
1680	Cl_clus101741_Contig1	---NA---	445	0 -	-	
1681	Cl_clus101739_Contig1	---NA---	263	0 -	-	
1682	Cl_clus101742_Contig1	---NA---	377	0 -	-	
1683	Cl_clus101743_Contig1	---NA---	496	0 -	-	
1684	Cl_clus101738_Contig1	---NA---	488	0 -	-	
1685	Cl_clus101744_Contig1	---NA---	589	0 -	-	
1686	Cl_clus101745_Contig1	---NA---	342	0 -	-	
1687	Cl_clus101746_Contig1	drosophila melanogaster ryanodine receptor 44f (rya-r44f) transcript variant mrna	539	18	8.50E-147	79.17%
1688	Cl_clus101747_Contig1	---NA---	430	0 -	-	
1689	Cl_clus101748_Contig1	---NA---	270	0 -	-	
1690	Cl_clus101749_Contig1	---NA---	291	0 -	-	
1691	Cl_clus101751_Contig1	---NA---	486	0 -	-	
1692	Cl_clus101752_Contig1	---NA---	313	0 -	-	
1693	Cl_clus101754_Contig1	---NA---	305	0 -	-	
1694	Cl_clus101755_Contig1	bombus impatiens band 7 protein agap004871-like mrna	361	2	4.60E-15	73.00%
1695	Cl_clus101756_Contig1	---NA---	232	0 -	-	
1696	Cl_clus101757_Contig1	---NA---	241	0 -	-	
1697	Cl_clus101758_Contig1	---NA---	241	0 -	-	
1698	Cl_clus101760_Contig1	---NA---	271	0 -	-	
1699	Cl_clus101761_Contig1	---NA---	291	0 -	-	
1700	Cl_clus101762_Contig1	---NA---	365	0 -	-	
1701	Cl_clus101759_Contig1	---NA---	340	0 -	-	
1702	Cl_clus101763_Contig1	---NA---	331	0 -	-	
1703	Cl_clus101764_Contig1	---NA---	207	0 -	-	
1704	Cl_clus101765_Contig1	---NA---	230	0 -	-	
1705	Cl_clus101766_Contig1	---NA---	392	0 -	-	
1706	Cl_clus101768_Contig1	---NA---	246	0 -	-	
1707	Cl_clus101770_Contig1	---NA---	241	0 -	-	
1708	Cl_clus101769_Contig1	---NA---	357	0 -	-	
1709	Cl_clus101771_Contig1	---NA---	322	0 -	-	
1710	Cl_clus101772_Contig1	---NA---	422	0 -	-	
1711	Cl_clus101773_Contig1	---NA---	368	0 -	-	
1712	Cl_clus101774_Contig1	---NA---	233	0 -	-	
1713	Cl_clus101776_Contig1	---NA---	201	0 -	-	
1714	Cl_clus101777_Contig1	---NA---	241	0 -	-	
1715	Cl_clus101779_Contig1	oreochromis niloticus succinate dehydrogenase	420	4	1.20E-86	74.75%
1716	Cl_clus101780_Contig1	---NA---	244	0 -	-	
1717	Cl_clus101775_Contig1	---NA---	374	0 -	-	
1718	Cl_clus101782_Contig1	---NA---	301	0 -	-	
1719	Cl_clus101784_Contig1	---NA---	312	0 -	-	
1720	Cl_clus101781_Contig1	---NA---	361	0 -	-	
1721	Cl_clus101785_Contig1	---NA---	355	0 -	-	
1722	Cl_clus101767_Contig1	---NA---	304	0 -	-	
1723	Cl_clus101787_Contig1	---NA---	390	0 -	-	
1724	Cl_clus101788_Contig1	---NA---	309	0 -	-	
1725	Cl_clus101789_Contig1	---NA---	488	0 -	-	
1726	Cl_clus101790_Contig1	---NA---	252	0 -	-	
1727	Cl_clus101786_Contig1	anopheles gambiae pest agap003857-pa complete cds	290	3	2.10E-24	81.00%
1728	Cl_clus101791_Contig1	---NA---	351	0 -	-	
1729	Cl_clus101792_Contig1	---NA---	280	0 -	-	
1730	Cl_clus101793_Contig1	---NA---	357	0 -	-	
1731	Cl_clus101795_Contig1	---NA---	564	0 -	-	

1732	Cl_clus101796_Contig1	---NA---	230	0 -	-	
1733	Cl_clus101798_Contig1	---NA---	312	0 -	-	
1734	Cl_clus101799_Contig1	---NA---	252	0 -	-	
1735	Cl_clus101800_Contig1	---NA---	226	0 -	-	
1736	Cl_clus101803_Contig1	---NA---	301	0 -	-	
1737	Cl_clus101801_Contig1	---NA---	532	0 -	-	
1738	Cl_clus101802_Contig1	---NA---	254	0 -	-	
1739	Cl_clus101808_Contig1	---NA---	229	0 -	-	
1740	Cl_clus101804_Contig1	---NA---	297	0 -	-	
1741	Cl_clus101805_Contig1	---NA---	267	0 -	-	
1742	Cl_clus101810_Contig1	---NA---	325	0 -	-	
1743	Cl_clus101807_Contig1	---NA---	219	0 -	-	
1744	Cl_clus101812_Contig1	---NA---	321	0 -	-	
1745	Cl_clus101813_Contig1	---NA---	270	0 -	-	
1746	Cl_clus101814_Contig1	---NA---	390	0 -	-	
1747	Cl_clus101818_Contig1	---NA---	439	0 -	-	
1748	Cl_clus101819_Contig1	---NA---	420	0 -	-	
1749	Cl_clus101809_Contig1	---NA---	287	0 -	-	
1750	Cl_clus101817_Contig1	---NA---	366	0 -	-	
1751	Cl_clus101822_Contig1	---NA---	338	0 -	-	
1752	Cl_clus101824_Contig1	---NA---	239	0 -	-	
1753	Cl_clus101825_Contig1	---NA---	304	0 -	-	
1754	Cl_clus101826_Contig1	---NA---	407	0 -	-	
1755	Cl_clus101815_Contig1	oncomelania hupensis hupensis thioredoxin peroxidase complete cds	424	1	5.10E-28	70.00%
1756	Cl_clus101827_Contig1	---NA---	308	0 -	-	
1757	Cl_clus101828_Contig1	---NA---	206	0 -	-	
1758	Cl_clus101829_Contig1	---NA---	590	0 -	-	
1759	Cl_clus101830_Contig1	---NA---	289	0 -	-	
1760	Cl_clus101831_Contig1	---NA---	423	0 -	-	
1761	Cl_clus101832_Contig1	---NA---	215	0 -	-	

1762	Cl_clus101833_Contig1	coptotermes formosanus clone cfsni971 serine-arginine protein 55-like protein partial cds	220	8	1.50E-62	92.50%
1763	Cl_clus101834_Contig1	---NA---	362	0 -	-	
1764	Cl_clus101835_Contig1	---NA---	275	0 -	-	
1765	Cl_clus101836_Contig1	---NA---	219	0 -	-	
1766	Cl_clus101838_Contig1	---NA---	281	0 -	-	
1767	Cl_clus101839_Contig1	---NA---	242	0 -	-	
1768	Cl_clus101840_Contig1	---NA---	378	0 -	-	
1769	Cl_clus101841_Contig1	---NA---	373	0 -	-	
1770	Cl_clus101842_Contig1	---NA---	318	0 -	-	
1771	Cl_clus101843_Contig1	setaria italica cysteine desulfurase mitochondrial-like transcript variant mrna	289	3	4.60E-20	81.33%
1772	Cl_clus101844_Contig1	---NA---	266	0 -	-	
1773	Cl_clus101845_Contig1	---NA---	201	0 -	-	
1774	Cl_clus101846_Contig1	---NA---	584	0 -	-	
1775	Cl_clus101847_Contig1	---NA---	634	0 -	-	
1776	Cl_clus101848_Contig1	---NA---	422	0 -	-	
1777	Cl_clus101850_Contig1	---NA---	446	0 -	-	
1778	Cl_clus101851_Contig1	---NA---	327	0 -	-	
1779	Cl_clus101852_Contig1	---NA---	277	0 -	-	
1780	Cl_clus101853_Contig1	---NA---	408	0 -	-	
1781	Cl_clus101854_Contig1	xenopus laevis succinate dehydrogenase subunit flavoprotein mrna	257	1	1.60E-12	71.00%
1782	Cl_clus101856_Contig1	---NA---	408	0 -	-	
1783	Cl_clus101857_Contig1	---NA---	476	0 -	-	
1784	Cl_clus101858_Contig1	---NA---	204	0 -	-	
1785	Cl_clus101860_Contig1	---NA---	264	0 -	-	
1786	Cl_clus101861_Contig1	---NA---	319	0 -	-	
1787	Cl_clus101862_Contig1	---NA---	266	0 -	-	
1788	Cl_clus101859_Contig1	---NA---	378	0 -	-	
1789	Cl_clus101863_Contig1	---NA---	393	0 -	-	

1790	Cl_clus101864_Contig1	---NA---	326	0 -	-	
1791	Cl_clus101865_Contig1	---NA---	258	0 -	-	
1792	Cl_clus101866_Contig1	---NA---	233	0 -	-	
1793	Cl_clus101868_Contig1	---NA---	287	0 -	-	
1794	Cl_clus101870_Contig1	---NA---	412	0 -	-	
1795	Cl_clus101871_Contig1	---NA---	464	0 -	-	
1796	Cl_clus101873_Contig1	---NA---	313	0 -	-	
1797	Cl_clus101875_Contig1	---NA---	338	0 -	-	
1798	Cl_clus101867_Contig1	---NA---	280	0 -	-	
1799	Cl_clus101874_Contig1	---NA---	498	0 -	-	
1800	Cl_clus101876_Contig1	---NA---	433	0 -	-	
1801	Cl_clus101877_Contig1	---NA---	476	0 -	-	
1802	Cl_clus101880_Contig1	---NA---	239	0 -	-	
1803	Cl_clus101881_Contig1	---NA---	294	0 -	-	
1804	Cl_clus101882_Contig1	armigeres subalbatus asap id: 40935 single-stranded dna binding mrna sequence	312	2	9.70E-42	72.00%
1805	Cl_clus101883_Contig1	---NA---	397	0 -	-	
1806	Cl_clus101884_Contig1	---NA---	205	0 -	-	
1807	Cl_clus101885_Contig1	---NA---	520	0 -	-	
1808	Cl_clus101886_Contig1	---NA---	396	0 -	-	
1809	Cl_clus101889_Contig1	---NA---	248	0 -	-	
1810	Cl_clus101887_Contig1	---NA---	400	0 -	-	
1811	Cl_clus101890_Contig1	---NA---	299	0 -	-	
1812	Cl_clus101891_Contig1	---NA---	246	0 -	-	
1813	Cl_clus101892_Contig1	---NA---	257	0 -	-	
1814	Cl_clus101893_Contig1	---NA---	350	0 -	-	
1815	Cl_clus101894_Contig1	---NA---	429	0 -	-	
1816	Cl_clus101895_Contig1	---NA---	308	0 -	-	
1817	Cl_clus101896_Contig1	---NA---	301	0 -	-	
1818	Cl_clus101897_Contig1	---NA---	370	0 -	-	
1819	Cl_clus101898_Contig1	---NA---	351	0 -	-	
1820	Cl_clus101899_Contig1	---NA---	218	0 -	-	
1821	Cl_clus101900_Contig1	gallus gallus tubulin alpha-4a chain-like transcript variant mrna	298	20	4.80E-58	86.00%
1822	Cl_clus101901_Contig1	maylandia zebra cytoplasmic 2-like transcript variant mrna	246	20	6.10E-43	84.20%
1823	Cl_clus101902_Contig1	---NA---	357	0 -	-	
1824	Cl_clus101904_Contig1	---NA---	417	0 -	-	
1825	Cl_clus101905_Contig1	---NA---	354	0 -	-	
1826	Cl_clus101906_Contig1	drosophila mojavensis gi23713 (dmoj\gi23713) mrna	354	6	5.80E-20	90.33%
1827	Cl_clus101907_Contig1	---NA---	251	0 -	-	
1828	Cl_clus101908_Contig1	---NA---	397	0 -	-	
1829	Cl_clus101909_Contig1	---NA---	229	0 -	-	
1830	Cl_clus101910_Contig1	---NA---	334	0 -	-	
1831	Cl_clus101911_Contig1	---NA---	222	0 -	-	
1832	Cl_clus101912_Contig1	---NA---	277	0 -	-	
1833	Cl_clus101913_Contig1	---NA---	371	0 -	-	
1834	Cl_clus101914_Contig1	---NA---	200	0 -	-	
1835	Cl_clus101916_Contig1	---NA---	207	0 -	-	
1836	Cl_clus101917_Contig1	---NA---	288	0 -	-	
1837	Cl_clus101919_Contig1	---NA---	618	0 -	-	
1838	Cl_clus101920_Contig1	---NA---	214	0 -	-	
1839	Cl_clus101918_Contig1	---NA---	277	0 -	-	
1840	Cl_clus101921_Contig1	lycorea halia na+ + atpase alpha-subunit 1 complete cds	371	1	3.60E-67	76.00%
1841	Cl_clus101922_Contig1	---NA---	403	0 -	-	
1842	Cl_clus101923_Contig1	---NA---	277	0 -	-	
1843	Cl_clus101924_Contig1	---NA---	327	0 -	-	
1844	Cl_clus101925_Contig1	---NA---	258	0 -	-	
1845	Cl_clus101926_Contig1	gryllus bimaculatus gbcontig22909	434	1	3.80E-36	91.00%
1846	Cl_clus101928_Contig1	---NA---	367	0 -	-	
1847	Cl_clus101929_Contig1	---NA---	456	0 -	-	
1848	Cl_clus101930_Contig1	---NA---	246	0 -	-	

1849	Cl_clus101903_Contig1	---NA---	310	0 -	-	
1850	Cl_clus101931_Contig1	---NA---	299	0 -	-	
1851	Cl_clus101933_Contig1	---NA---	245	0 -	-	
1852	Cl_clus101934_Contig1	---NA---	358	0 -	-	
1853	Cl_clus101935_Contig1	---NA---	409	0 -	-	
1854	Cl_clus101932_Contig1	---NA---	285	0 -	-	
1855	Cl_clus101936_Contig1	---NA---	211	0 -	-	
1856	Cl_clus101937_Contig1	---NA---	215	0 -	-	
1857	Cl_clus101938_Contig1	---NA---	449	0 -	-	
1858	Cl_clus101939_Contig1	---NA---	481	0 -	-	
1859	Cl_clus101940_Contig1	---NA---	466	0 -	-	
1860	Cl_clus101941_Contig1	---NA---	359	0 -	-	
1861	Cl_clus101942_Contig1	---NA---	230	0 -	-	
1862	Cl_clus101943_Contig1	---NA---	280	0 -	-	
1863	Cl_clus101944_Contig1	---NA---	405	0 -	-	
1864	Cl_clus101945_Contig1	---NA---	306	0 -	-	
1865	Cl_clus101946_Contig1	---NA---	396	0 -	-	
1866	Cl_clus101948_Contig1	---NA---	205	0 -	-	
1867	Cl_clus101949_Contig1	---NA---	343	0 -	-	
1868	Cl_clus101950_Contig1	---NA---	353	0 -	-	
1869	Cl_clus101951_Contig1	---NA---	214	0 -	-	
1870	Cl_clus101952_Contig1	uroptychus scambus sodium potassium atpase alpha subunit partial cds	258	20	3.10E-53	75.65%
1871	Cl_clus101953_Contig1	---NA---	349	0 -	-	
1872	Cl_clus101954_Contig1	---NA---	483	0 -	-	
1873	Cl_clus101955_Contig1	---NA---	312	0 -	-	
1874	Cl_clus101956_Contig1	---NA---	218	0 -	-	
1875	Cl_clus101957_Contig1	---NA---	586	0 -	-	
1876	Cl_clus101958_Contig1	---NA---	230	0 -	-	
1877	Cl_clus101959_Contig1	---NA---	530	0 -	-	
1878	Cl_clus101963_Contig1	meleagris gallopavo phosphoglycerate kinase-like mrna	612	6	6.60E-73	69.33%
1879	Cl_clus101964_Contig1	---NA---	307	0 -	-	
1880	Cl_clus101960_Contig1	---NA---	404	0 -	-	
1881	Cl_clus101965_Contig1	---NA---	307	0 -	-	
1882	Cl_clus101966_Contig1	---NA---	273	0 -	-	
1883	Cl_clus101967_Contig1	---NA---	251	0 -	-	
1884	Cl_clus101968_Contig1	---NA---	257	0 -	-	
1885	Cl_clus101969_Contig1	---NA---	246	0 -	-	
1886	Cl_clus101970_Contig1	---NA---	386	0 -	-	
1887	Cl_clus101971_Contig1	---NA---	313	0 -	-	
1888	Cl_clus101972_Contig1	---NA---	316	0 -	-	
1889	Cl_clus101973_Contig1	---NA---	400	0 -	-	
1890	Cl_clus101974_Contig1	---NA---	423	0 -	-	
1891	Cl_clus101975_Contig1	---NA---	275	0 -	-	
1892	Cl_clus101976_Contig1	---NA---	269	0 -	-	
1893	Cl_clus101978_Contig1	---NA---	250	0 -	-	
1894	Cl_clus101979_Contig1	---NA---	202	0 -	-	
1895	Cl_clus101981_Contig1	---NA---	621	0 -	-	
1896	Cl_clus101982_Contig1	anas platyrhynchos polymerase ii (dna directed) polypeptide h partial mrna	430	3	1.10E-55	76.67%
1897	Cl_clus101977_Contig1	---NA---	405	0 -	-	
1898	Cl_clus101983_Contig1	---NA---	271	0 -	-	
1899	Cl_clus101984_Contig1	---NA---	207	0 -	-	
1900	Cl_clus101985_Contig1	---NA---	409	0 -	-	
1901	Cl_clus101986_Contig1	---NA---	231	0 -	-	
1902	Cl_clus101987_Contig1	---NA---	215	0 -	-	
1903	Cl_clus101988_Contig1	---NA---	304	0 -	-	
1904	Cl_clus101989_Contig1	---NA---	276	0 -	-	
1905	Cl_clus101990_Contig1	---NA---	259	0 -	-	
1906	Cl_clus101991_Contig1	---NA---	243	0 -	-	

1907	Cl_clus101993_Contig1	---NA---	426	0	-	-	
1908	Cl_clus101994_Contig1	---NA---	299	0	-	-	
1909	Cl_clus101995_Contig1	---NA---	536	0	-	-	
1910	Cl_clus101996_Contig1	---NA---	324	0	-	-	
1911	Cl_clus101997_Contig1	---NA---	393	0	-	-	
1912	Cl_clus101998_Contig1	---NA---	335	0	-	-	
1913	Cl_clus102000_Contig1	---NA---	525	0	-	-	
1914	Cl_clus102001_Contig1	---NA---	596	0	-	-	
1915	Cl_clus102004_Contig1	---NA---	318	0	-	-	
1916	Cl_clus102002_Contig1	---NA---	291	0	-	-	
1917	Cl_clus102005_Contig1	cnaphalocrocis medinalis ryanodine receptor partial cds	297	2	4.20E-84		83.00%
1918	Cl_clus102006_Contig1	---NA---	274	0	-	-	
1919	Cl_clus102008_Contig1	---NA---	407	0	-	-	
1920	Cl_clus102009_Contig1	---NA---	488	0	-	-	
1921	Cl_clus102010_Contig1	---NA---	326	0	-	-	
1922	Cl_clus102007_Contig1	---NA---	342	0	-	-	
1923	Cl_clus102011_Contig1	---NA---	235	0	-	-	
1924	Cl_clus102012_Contig1	---NA---	300	0	-	-	
1925	Cl_clus101999_Contig1	---NA---	468	0	-	-	
1926	Cl_clus102013_Contig1	---NA---	245	0	-	-	
1927	Cl_clus102014_Contig1	---NA---	418	0	-	-	
1928	Cl_clus102016_Contig1	---NA---	748	0	-	-	
1929	Cl_clus102017_Contig1	---NA---	304	0	-	-	
1930	Cl_clus102018_Contig1	---NA---	456	0	-	-	
1931	Cl_clus102020_Contig1	---NA---	451	0	-	-	
1932	Cl_clus102021_Contig1	---NA---	210	0	-	-	
1933	Cl_clus102019_Contig1	---NA---	207	0	-	-	
1934	Cl_clus102022_Contig1	---NA---	437	0	-	-	
1935	Cl_clus102023_Contig1	---NA---	284	0	-	-	
1936	Cl_clus102024_Contig1	---NA---	565	0	-	-	
1937	Cl_clus102026_Contig1	---NA---	229	0	-	-	
1938	Cl_clus102027_Contig1	---NA---	363	0	-	-	
1939	Cl_clus102028_Contig1	---NA---	276	0	-	-	
1940	Cl_clus102029_Contig1	---NA---	248	0	-	-	
1941	Cl_clus102030_Contig1	---NA---	373	0	-	-	
1942	Cl_clus102032_Contig1	---NA---	206	0	-	-	
1943	Cl_clus102033_Contig1	---NA---	399	0	-	-	
1944	Cl_clus102034_Contig1	---NA---	322	0	-	-	
1945	Cl_clus102035_Contig1	---NA---	360	0	-	-	
1946	Cl_clus102036_Contig1	---NA---	298	0	-	-	
1947	Cl_clus102038_Contig1	---NA---	374	0	-	-	
1948	Cl_clus102039_Contig1	---NA---	445	0	-	-	
1949	Cl_clus102031_Contig1	---NA---	311	0	-	-	
1950	Cl_clus102037_Contig1	---NA---	257	0	-	-	
1951	Cl_clus102040_Contig1	---NA---	248	0	-	-	
1952	Cl_clus102041_Contig1	---NA---	210	0	-	-	
1953	Cl_clus102043_Contig1	---NA---	343	0	-	-	
1954	Cl_clus102044_Contig1	---NA---	210	0	-	-	
1955	Cl_clus102045_Contig1	---NA---	245	0	-	-	
1956	Cl_clus102046_Contig1	---NA---	250	0	-	-	
1957	Cl_clus102047_Contig1	---NA---	283	0	-	-	
1958	Cl_clus102048_Contig1	---NA---	379	0	-	-	
1959	Cl_clus102049_Contig1	---NA---	496	0	-	-	
1960	Cl_clus102050_Contig1	---NA---	389	0	-	-	
1961	Cl_clus102051_Contig1	---NA---	477	0	-	-	
1962	Cl_clus102053_Contig1	---NA---	788	0	-	-	
1963	Cl_clus102054_Contig1	---NA---	355	0	-	-	
1964	Cl_clus102056_Contig1	---NA---	314	0	-	-	
1965	Cl_clus102057_Contig1	---NA---	397	0	-	-	
1966	Cl_clus102058_Contig1	---NA---	379	0	-	-	

1967	Cl_clus102059_Contig1	---NA---	277	0 -	-	
1968	Cl_clus102060_Contig1	---NA---	390	0 -	-	
1969	Cl_clus102061_Contig1	---NA---	295	0 -	-	
1970	Cl_clus102062_Contig1	---NA---	441	0 -	-	
1971	Cl_clus102063_Contig1	---NA---	316	0 -	-	
1972	Cl_clus102064_Contig1	megachile rotundata 40s ribosomal protein s28-like mrna	362	1	4.00E-41	79.00%
1973	Cl_clus102065_Contig1	---NA---	206	0 -	-	
1974	Cl_clus102066_Contig1	---NA---	324	0 -	-	
1975	Cl_clus102067_Contig1	arabidopsis thaliana at4g21960 t8o5_170 complete cds	276	20	1.30E-121	93.05%
1976	Cl_clus102068_Contig1	---NA---	212	0 -	-	
1977	Cl_clus102069_Contig1	ictalurus furcatus clone cbzc20143 ras-related c3 botulinum toxin substrate 3 complete cds	333	3	1.70E-64	76.67%
1978	Cl_clus102070_Contig1	---NA---	267	0 -	-	
1979	Cl_clus102071_Contig1	---NA---	284	0 -	-	
1980	Cl_clus102072_Contig1	---NA---	492	0 -	-	
1981	Cl_clus102074_Contig1	---NA---	334	0 -	-	
1982	Cl_clus102075_Contig1	---NA---	392	0 -	-	
1983	Cl_clus102076_Contig1	---NA---	567	0 -	-	
1984	Cl_clus102077_Contig1	---NA---	431	0 -	-	
1985	Cl_clus102078_Contig1	---NA---	466	0 -	-	
1986	Cl_clus102079_Contig1	---NA---	421	0 -	-	
1987	Cl_clus102080_Contig1	---NA---	330	0 -	-	
1988	Cl_clus102081_Contig1	---NA---	288	0 -	-	
1989	Cl_clus102082_Contig1	---NA---	245	0 -	-	
1990	Cl_clus102083_Contig1	---NA---	254	0 -	-	
1991	Cl_clus102084_Contig1	---NA---	354	0 -	-	
1992	Cl_clus102086_Contig1	---NA---	510	0 -	-	
1993	Cl_clus102087_Contig1	---NA---	224	0 -	-	
1994	Cl_clus102088_Contig1	---NA---	457	0 -	-	
1995	Cl_clus102089_Contig1	---NA---	623	0 -	-	

1996	Cl_clus102090_Contig1	---NA---	437	0 -	-	
1997	Cl_clus102091_Contig1	---NA---	204	0 -	-	
1998	Cl_clus102092_Contig1	---NA---	366	0 -	-	
1999	Cl_clus102093_Contig1	---NA---	415	0 -	-	
2000	Cl_clus102094_Contig1	---NA---	210	0 -	-	
2001	Cl_clus102095_Contig1	---NA---	283	0 -	-	
2002	Cl_clus102096_Contig1	---NA---	471	0 -	-	
2003	Cl_clus102097_Contig1	---NA---	331	0 -	-	
2004	Cl_clus102100_Contig1	---NA---	461	0 -	-	
2005	Cl_clus102102_Contig1	---NA---	278	0 -	-	
2006	Cl_clus102103_Contig1	---NA---	315	0 -	-	
2007	Cl_clus102099_Contig2	---NA---	503	0 -	-	
2008	Cl_clus102101_Contig1	---NA---	307	0 -	-	
2009	Cl_clus102104_Contig1	---NA---	258	0 -	-	
2010	Cl_clus102105_Contig1	---NA---	349	0 -	-	
2011	Cl_clus102106_Contig1	---NA---	255	0 -	-	
2012	Cl_clus102107_Contig1	---NA---	216	0 -	-	
2013	Cl_clus102108_Contig1	---NA---	348	0 -	-	
2014	Cl_clus102109_Contig1	---NA---	264	0 -	-	
2015	Cl_clus102110_Contig1	---NA---	222	0 -	-	
2016	Cl_clus102113_Contig1	---NA---	216	0 -	-	
2017	Cl_clus102114_Contig1	---NA---	234	0 -	-	
2018	Cl_clus102115_Contig1	---NA---	414	0 -	-	
2019	Cl_clus102112_Contig1	---NA---	403	0 -	-	
2020	Cl_clus102116_Contig1	---NA---	286	0 -	-	
2021	Cl_clus102111_Contig1	---NA---	254	0 -	-	
2022	Cl_clus102117_Contig1	---NA---	258	0 -	-	
2023	Cl_clus102118_Contig1	---NA---	408	0 -	-	
2024	Cl_clus102119_Contig1	---NA---	488	0 -	-	
2025	Cl_clus102120_Contig1	---NA---	310	0 -	-	

2026	Cl_clus102121_Contig1	---NA---	308	0 -	-	
2027	Cl_clus102122_Contig1	---NA---	377	0 -	-	
2028	Cl_clus102123_Contig1	---NA---	248	0 -	-	
2029	Cl_clus102124_Contig1	---NA---	377	0 -	-	
2030	Cl_clus102125_Contig1	---NA---	343	0 -	-	
2031	Cl_clus102126_Contig1	---NA---	307	0 -	-	
2032	Cl_clus102127_Contig1	---NA---	377	0 -	-	
2033	Cl_clus102129_Contig1	---NA---	273	0 -	-	
2034	Cl_clus102130_Contig1	---NA---	329	0 -	-	
2035	Cl_clus102131_Contig1	---NA---	360	0 -	-	
2036	Cl_clus102132_Contig1	---NA---	224	0 -	-	
2037	Cl_clus102136_Contig1	---NA---	288	0 -	-	
2038	Cl_clus102133_Contig1	---NA---	400	0 -	-	
2039	Cl_clus102135_Contig1	---NA---	285	0 -	-	
2040	Cl_clus102137_Contig1	---NA---	251	0 -	-	
2041	Cl_clus102138_Contig1	---NA---	226	0 -	-	
2042	Cl_clus102140_Contig1	---NA---	214	0 -	-	
2043	Cl_clus102141_Contig1	---NA---	205	0 -	-	
2044	Cl_clus102142_Contig1	---NA---	277	0 -	-	
2045	Cl_clus102144_Contig1	---NA---	384	0 -	-	
2046	Cl_clus102139_Contig1	---NA---	376	0 -	-	
2047	Cl_clus102145_Contig1	---NA---	277	0 -	-	
2048	Cl_clus102147_Contig1	---NA---	283	0 -	-	
2049	Cl_clus102148_Contig1	---NA---	361	0 -	-	
2050	Cl_clus102149_Contig1	---NA---	286	0 -	-	
2051	Cl_clus102153_Contig1	---NA---	436	0 -	-	
2052	Cl_clus102146_Contig1	---NA---	414	0 -	-	
2053	Cl_clus102150_Contig1	---NA---	824	0 -	-	
2054	Cl_clus102151_Contig1	---NA---	283	0 -	-	
2055	Cl_clus102152_Contig1	---NA---	212	0 -	-	
2056	Cl_clus102154_Contig1	---NA---	242	0 -	-	
2057	Cl_clus102155_Contig1	---NA---	258	0 -	-	
2058	Cl_clus102156_Contig1	---NA---	319	0 -	-	
2059	Cl_clus102157_Contig1	---NA---	301	0 -	-	
2060	Cl_clus102158_Contig1	---NA---	329	0 -	-	
2061	Cl_clus102159_Contig1	---NA---	288	0 -	-	
2062	Cl_clus102161_Contig1	---NA---	458	0 -	-	
2063	Cl_clus102160_Contig1	---NA---	403	0 -	-	
2064	Cl_clus102162_Contig1	maylandia zebra ras-related protein rab-10-like transcript variant mrna	485	20	2.00E-122	74.70%
2065	Cl_clus102163_Contig1	---NA---	276	0 -	-	
2066	Cl_clus102164_Contig1	---NA---	255	0 -	-	
2067	Cl_clus102166_Contig1	---NA---	409	0 -	-	
2068	Cl_clus102165_Contig1	---NA---	756	0 -	-	
2069	Cl_clus102167_Contig1	---NA---	351	0 -	-	
2070	Cl_clus102169_Contig1	---NA---	298	0 -	-	
2071	Cl_clus102171_Contig1	---NA---	250	0 -	-	
2072	Cl_clus102173_Contig1	---NA---	321	0 -	-	
2073	Cl_clus102175_Contig1	ficedula albicollis nadh dehydrogenase fe-s protein 20kda (nadh-coenzyme q reductase) transcript variant mrna	453	4	6.20E-78	80.50%
2074	Cl_clus102176_Contig1	---NA---	384	0 -	-	
2075	Cl_clus102172_Contig1	---NA---	311	0 -	-	
2076	Cl_clus102174_Contig1	salpingoeca atcc 50818 atp-dependent rna helicase dbp2 complete cds	229	4	5.20E-37	76.75%
2077	Cl_clus102177_Contig1	---NA---	451	0 -	-	
2078	Cl_clus102178_Contig1	---NA---	460	0 -	-	
2079	Cl_clus102179_Contig1	---NA---	236	0 -	-	
2080	Cl_clus102180_Contig1	---NA---	272	0 -	-	
2081	Cl_clus102181_Contig1	---NA---	212	0 -	-	
2082	Cl_clus102182_Contig1	drosophila willistoni gk23721 (dwil\gk23721) mrna	512	5	1.30E-74	76.60%
2083	Cl_clus102183_Contig1	---NA---	290	0 -	-	
2084	Cl_clus102184_Contig1	---NA---	556	0 -	-	

2085 Cl_clus102186_Contig1	---NA---	510	0 -	-	
2086 Cl_clus102187_Contig1	ictalurus punctatus v-type proton atpase 16 kda proteolipid subunit mrna	245	6	6.90E-36	81.00%
2087 Cl_clus102188_Contig1	---NA---	490	0 -	-	
2088 Cl_clus102189_Contig1	---NA---	201	0 -	-	
2089 Cl_clus102191_Contig1	---NA---	271	0 -	-	
2090 Cl_clus102192_Contig1	oryctolagus cuniculus guanine nucleotide-binding beta-4 subunit-like mrna	279	2	3.40E-53	76.50%
2091 Cl_clus102194_Contig1	---NA---	402	0 -	-	
2092 Cl_clus102195_Contig1	---NA---	326	0 -	-	
2093 Cl_clus102196_Contig1	---NA---	262	0 -	-	
2094 Cl_clus102197_Contig1	---NA---	456	0 -	-	
2095 Cl_clus102198_Contig1	---NA---	413	0 -	-	
2096 Cl_clus102199_Contig1	---NA---	313	0 -	-	
2097 Cl_clus102200_Contig1	---NA---	269	0 -	-	
2098 Cl_clus102201_Contig1	---NA---	343	0 -	-	
2099 Cl_clus102202_Contig1	---NA---	260	0 -	-	
2100 Cl_clus102203_Contig1	---NA---	238	0 -	-	
2101 Cl_clus102204_Contig1	---NA---	309	0 -	-	
2102 Cl_clus102205_Contig1	---NA---	293	0 -	-	
2103 Cl_clus102207_Contig1	---NA---	350	0 -	-	
2104 Cl_clus102208_Contig1	---NA---	320	0 -	-	
2105 Cl_clus102209_Contig1	---NA---	268	0 -	-	
2106 Cl_clus102210_Contig1	---NA---	318	0 -	-	
2107 Cl_clus102211_Contig1	---NA---	206	0 -	-	
2108 Cl_clus102213_Contig1	---NA---	556	0 -	-	
2109 Cl_clus102214_Contig1	---NA---	230	0 -	-	
2110 Cl_clus102215_Contig1	---NA---	327	0 -	-	
2111 Cl_clus102216_Contig1	---NA---	293	0 -	-	
2112 Cl_clus102217_Contig1	---NA---	427	0 -	-	
2113 Cl_clus102218_Contig1	suberites fuscus isolate toa57 chaperonin complex component tcp-1 beta subunit partial cds	328	3	4.40E-40	72.00%
2114 Cl_clus102219_Contig1	---NA---	401	0 -	-	
2115 Cl_clus102220_Contig1	---NA---	339	0 -	-	
2116 Cl_clus102221_Contig1	---NA---	389	0 -	-	
2117 Cl_clus102222_Contig1	setaria italica adp-ribosylation factor-like transcript variant mrna	351	20	3.00E-55	77.95%
2118 Cl_clus102224_Contig1	---NA---	317	0 -	-	
2119 Cl_clus102225_Contig1	---NA---	343	0 -	-	
2120 Cl_clus102226_Contig1	---NA---	223	0 -	-	
2121 Cl_clus102227_Contig1	paracyclopina nana heat shock protein 70 complete cds	287	2	4.90E-26	72.00%
2122 Cl_clus102229_Contig1	---NA---	349	0 -	-	
2123 Cl_clus102230_Contig1	---NA---	437	0 -	-	
2124 Cl_clus102231_Contig1	---NA---	276	0 -	-	
2125 Cl_clus102232_Contig1	---NA---	645	0 -	-	
2126 Cl_clus102233_Contig1	---NA---	251	0 -	-	
2127 Cl_clus102234_Contig1	---NA---	340	0 -	-	
2128 Cl_clus102235_Contig1	---NA---	296	0 -	-	
2129 Cl_clus102236_Contig1	---NA---	208	0 -	-	
2130 Cl_clus102237_Contig1	---NA---	439	0 -	-	
2131 Cl_clus102239_Contig1	drosophila ananassae gf13398 (dana\gf13398) mrna	223	1	4.70E-50	85.00%
2132 Cl_clus102238_Contig1	---NA---	277	0 -	-	
2133 Cl_clus102240_Contig1	---NA---	418	0 -	-	
2134 Cl_clus102241_Contig1	---NA---	227	0 -	-	
2135 Cl_clus102242_Contig1	---NA---	270	0 -	-	
2136 Cl_clus102243_Contig1	---NA---	445	0 -	-	
2137 Cl_clus102244_Contig1	---NA---	342	0 -	-	
2138 Cl_clus102245_Contig1	---NA---	272	0 -	-	
2139 Cl_clus102246_Contig1	---NA---	265	0 -	-	
2140 Cl_clus102247_Contig1	---NA---	375	0 -	-	
2141 Cl_clus102248_Contig1	---NA---	437	0 -	-	

2142	Cl_clus102249_Contig1	---NA---	307	0 -	-	
2143	Cl_clus102251_Contig1	---NA---	303	0 -	-	
2144	Cl_clus102250_Contig1	---NA---	277	0 -	-	
2145	Cl_clus102252_Contig1	---NA---	200	0 -	-	
2146	Cl_clus102253_Contig1	---NA---	413	0 -	-	
2147	Cl_clus102255_Contig1	---NA---	377	0 -	-	
2148	Cl_clus102257_Contig1	---NA---	536	0 -	-	
2149	Cl_clus102258_Contig1	---NA---	287	0 -	-	
2150	Cl_clus102260_Contig1	---NA---	311	0 -	-	
2151	Cl_clus102261_Contig1	---NA---	548	0 -	-	
2152	Cl_clus102262_Contig1	---NA---	346	0 -	-	
2153	Cl_clus102263_Contig1	---NA---	558	0 -	-	
2154	Cl_clus102264_Contig1	---NA---	334	0 -	-	
2155	Cl_clus102265_Contig1	---NA---	478	0 -	-	
2156	Cl_clus102266_Contig1	---NA---	208	0 -	-	
2157	Cl_clus102267_Contig1	---NA---	341	0 -	-	
2158	Cl_clus102268_Contig1	---NA---	315	0 -	-	
2159	Cl_clus102269_Contig1	---NA---	325	0 -	-	
2160	Cl_clus102270_Contig1	---NA---	616	0 -	-	
2161	Cl_clus102271_Contig1	---NA---	265	0 -	-	
2162	Cl_clus102272_Contig1	---NA---	453	0 -	-	
2163	Cl_clus102273_Contig1	---NA---	284	0 -	-	
2164	Cl_clus102274_Contig1	---NA---	415	0 -	-	
2165	Cl_clus102276_Contig1	---NA---	371	0 -	-	
2166	Cl_clus102277_Contig1	---NA---	316	0 -	-	
2167	Cl_clus102278_Contig1	---NA---	262	0 -	-	
2168	Cl_clus102279_Contig1	ficedula albicollis tubulin alpha-1c chain-like transcript variant mrna	257	17	1.30E-51	82.41%
2169	Cl_clus102280_Contig1	---NA---	248	0 -	-	
2170	Cl_clus102282_Contig1	---NA---	506	0 -	-	
2171	Cl_clus102283_Contig1	---NA---	516	0 -	-	
2172	Cl_clus102284_Contig1	---NA---	276	0 -	-	
2173	Cl_clus102285_Contig1	---NA---	439	0 -	-	
2174	Cl_clus102286_Contig1	---NA---	252	0 -	-	
2175	Cl_clus102287_Contig1	---NA---	267	0 -	-	
2176	Cl_clus102288_Contig1	---NA---	210	0 -	-	
2177	Cl_clus102289_Contig1	---NA---	347	0 -	-	
2178	Cl_clus102290_Contig1	---NA---	465	0 -	-	
2179	Cl_clus102291_Contig1	---NA---	351	0 -	-	
2180	Cl_clus102292_Contig1	---NA---	225	0 -	-	
2181	Cl_clus102293_Contig1	capsaspora owczarzaki atcc 30864 superoxide dismutase cu-zn complete cds	393	6	2.40E-25	73.33%
2182	Cl_clus102294_Contig1	---NA---	416	0 -	-	
2183	Cl_clus102297_Contig1	---NA---	421	0 -	-	
2184	Cl_clus102298_Contig1	---NA---	404	0 -	-	
2185	Cl_clus102300_Contig1	---NA---	420	0 -	-	
2186	Cl_clus102295_Contig1	---NA---	571	0 -	-	
2187	Cl_clus102301_Contig1	---NA---	297	0 -	-	
2188	Cl_clus102302_Contig1	---NA---	434	0 -	-	
2189	Cl_clus102303_Contig1	---NA---	373	0 -	-	
2190	Cl_clus102304_Contig1	---NA---	400	0 -	-	
2191	Cl_clus102305_Contig1	---NA---	350	0 -	-	
2192	Cl_clus102306_Contig1	---NA---	251	0 -	-	
2193	Cl_clus102308_Contig1	---NA---	424	0 -	-	
2194	Cl_clus102309_Contig1	---NA---	248	0 -	-	
2195	Cl_clus102310_Contig1	---NA---	533	0 -	-	
2196	Cl_clus102311_Contig1	---NA---	584	0 -	-	
2197	Cl_clus102312_Contig1	---NA---	366	0 -	-	
2198	Cl_clus102313_Contig1	---NA---	235	0 -	-	
2199	Cl_clus102314_Contig1	---NA---	286	0 -	-	
2200	Cl_clus102315_Contig1	---NA---	221	0 -	-	

2201	Cl_clus102316_Contig1	---NA---	310	0 -	-	
2202	Cl_clus102317_Contig1	---NA---	290	0 -	-	
2203	Cl_clus102318_Contig1	---NA---	391	0 -	-	
2204	Cl_clus102321_Contig1	---NA---	227	0 -	-	
2205	Cl_clus102322_Contig1	---NA---	281	0 -	-	
2206	Cl_clus102323_Contig1	---NA---	339	0 -	-	
2207	Cl_clus102324_Contig1	---NA---	328	0 -	-	
2208	Cl_clus102325_Contig1	---NA---	382	0 -	-	
2209	Cl_clus102326_Contig1	---NA---	642	0 -	-	
2210	Cl_clus102327_Contig1	---NA---	413	0 -	-	
2211	Cl_clus102328_Contig1	---NA---	246	0 -	-	
2212	Cl_clus102329_Contig1	---NA---	273	0 -	-	
2213	Cl_clus102330_Contig1	---NA---	350	0 -	-	
2214	Cl_clus102331_Contig1	---NA---	599	0 -	-	
2215	Cl_clus102332_Contig1	---NA---	494	0 -	-	
2216	Cl_clus102333_Contig1	---NA---	341	0 -	-	
2217	Cl_clus102334_Contig1	---NA---	331	0 -	-	
2218	Cl_clus102335_Contig1	---NA---	207	0 -	-	
2219	Cl_clus102336_Contig1	---NA---	294	0 -	-	
2220	Cl_clus102338_Contig1	---NA---	243	0 -	-	
2221	Cl_clus102340_Contig1	oreochromis niloticus utp--glucose-1-phosphate uridylyltransferase-like mrna	362	5	4.00E-22	71.00%
2222	Cl_clus102341_Contig1	---NA---	213	0 -	-	
2223	Cl_clus102343_Contig1	---NA---	431	0 -	-	
2224	Cl_clus102339_Contig1	---NA---	223	0 -	-	
2225	Cl_clus102344_Contig1	---NA---	352	0 -	-	
2226	Cl_clus102345_Contig1	---NA---	262	0 -	-	
2227	Cl_clus102346_Contig1	---NA---	247	0 -	-	
2228	Cl_clus102347_Contig1	---NA---	240	0 -	-	
2229	Cl_clus102348_Contig1	---NA---	228	0 -	-	
2230	Cl_clus102349_Contig1	---NA---	377	0 -	-	
2231	Cl_clus102337_Contig1	---NA---	302	0 -	-	
2232	Cl_clus102350_Contig1	---NA---	308	0 -	-	
2233	Cl_clus102351_Contig1	---NA---	513	0 -	-	
2234	Cl_clus102352_Contig1	---NA---	462	0 -	-	
2235	Cl_clus102353_Contig1	---NA---	308	0 -	-	
2236	Cl_clus102354_Contig1	---NA---	483	0 -	-	
2237	Cl_clus102355_Contig1	osmerus mordax clone omor-eva-518-227 40s ribosomal protein s3 complete cds	257	20	1.60E-69	81.00%
2238	Cl_clus102356_Contig1	---NA---	397	0 -	-	
2239	Cl_clus102357_Contig1	---NA---	338	0 -	-	
2240	Cl_clus102358_Contig1	---NA---	327	0 -	-	
2241	Cl_clus102359_Contig1	---NA---	346	0 -	-	
2242	Cl_clus102360_Contig1	---NA---	338	0 -	-	
2243	Cl_clus102361_Contig1	---NA---	255	0 -	-	
2244	Cl_clus102362_Contig1	---NA---	478	0 -	-	
2245	Cl_clus102364_Contig1	---NA---	239	0 -	-	
2246	Cl_clus102365_Contig1	---NA---	476	0 -	-	
2247	Cl_clus102366_Contig1	---NA---	344	0 -	-	
2248	Cl_clus102367_Contig1	---NA---	781	0 -	-	
2249	Cl_clus102368_Contig1	---NA---	333	0 -	-	
2250	Cl_clus102370_Contig1	arabidopsis thaliana photosystem ii type i chlorophyll a b binding protein complete cds	224	20	2.80E-97	95.00%
2251	Cl_clus102371_Contig1	ixodes pacificus clone ip_7_60_92_475_clu ribosomal protein l41 complete cds	293	3	9.10E-23	92.00%
2252	Cl_clus102372_Contig1	---NA---	259	0 -	-	
2253	Cl_clus102373_Contig1	---NA---	362	0 -	-	
2254	Cl_clus102374_Contig1	---NA---	240	0 -	-	
2255	Cl_clus102375_Contig1	---NA---	205	0 -	-	
2256	Cl_clus102376_Contig1	---NA---	276	0 -	-	

2257	Cl_clus102377_Contig1	---NA---	303	0	-	-
2258	Cl_clus102378_Contig1	---NA---	530	0	-	-
2259	Cl_clus102379_Contig1	---NA---	341	0	-	-
2260	Cl_clus102381_Contig1	---NA---	229	0	-	-
2261	Cl_clus102382_Contig1	---NA---	656	0	-	-
2262	Cl_clus102383_Contig1	---NA---	205	0	-	-
2263	Cl_clus102384_Contig1	---NA---	287	0	-	-
2264	Cl_clus102385_Contig1	---NA---	320	0	-	-
2265	Cl_clus102386_Contig1	---NA---	210	0	-	-
2266	Cl_clus102387_Contig1	---NA---	270	0	-	-
2267	Cl_clus102388_Contig1	---NA---	256	0	-	-
2268	Cl_clus102389_Contig1	---NA---	393	0	-	-
2269	Cl_clus102390_Contig1	---NA---	221	0	-	-
2270	Cl_clus102391_Contig1	---NA---	327	0	-	-
2271	Cl_clus102392_Contig1	---NA---	336	0	-	-
2272	Cl_clus102393_Contig1	---NA---	811	0	-	-
2273	Cl_clus102394_Contig1	---NA---	484	0	-	-
2274	Cl_clus102395_Contig1	---NA---	498	0	-	-
2275	Cl_clus102396_Contig1	---NA---	258	0	-	-
2276	Cl_clus102398_Contig1	---NA---	308	0	-	-
2277	Cl_clus102401_Contig1	---NA---	476	0	-	-
2278	Cl_clus102403_Contig1	---NA---	251	0	-	-
2279	Cl_clus102404_Contig1	---NA---	259	0	-	-
2280	Cl_clus102405_Contig1	---NA---	374	0	-	-
2281	Cl_clus102406_Contig1	---NA---	403	0	-	-
2282	Cl_clus102407_Contig1	---NA---	206	0	-	-
2283	Cl_clus102408_Contig1	---NA---	384	0	-	-
2284	Cl_clus102409_Contig1	---NA---	394	0	-	-
2285	Cl_clus102410_Contig1	---NA---	327	0	-	-
2286	Cl_clus102411_Contig1	---NA---	214	0	-	-
2287	Cl_clus102412_Contig1	---NA---	355	0	-	-
2288	Cl_clus102414_Contig1	---NA---	396	0	-	-
2289	Cl_clus102416_Contig1	---NA---	205	0	-	-
2290	Cl_clus102413_Contig1	---NA---	382	0	-	-
2291	Cl_clus102418_Contig1	---NA---	221	0	-	-
2292	Cl_clus102419_Contig1	---NA---	333	0	-	-
2293	Cl_clus102422_Contig1	---NA---	396	0	-	-
2294	Cl_clus102424_Contig1	---NA---	333	0	-	-
2295	Cl_clus102425_Contig1	---NA---	248	0	-	-
2296	Cl_clus102427_Contig1	---NA---	533	0	-	-
2297	Cl_clus102428_Contig1	---NA---	464	0	-	-
2298	Cl_clus102429_Contig1	---NA---	276	0	-	-
2299	Cl_clus102431_Contig1	---NA---	329	0	-	-
2300	Cl_clus102432_Contig1	---NA---	218	0	-	-
2301	Cl_clus102430_Contig1	---NA---	282	0	-	-
2302	Cl_clus102433_Contig1	---NA---	277	0	-	-
2303	Cl_clus102434_Contig1	---NA---	201	0	-	-
2304	Cl_clus102436_Contig1	---NA---	761	0	-	-
2305	Cl_clus102437_Contig1	---NA---	224	0	-	-
2306	Cl_clus102439_Contig1	---NA---	418	0	-	-
2307	Cl_clus102440_Contig1	---NA---	306	0	-	-
2308	Cl_clus102438_Contig1	---NA---	202	0	-	-
2309	Cl_clus102442_Contig1	megachile rotundata f-box only protein 28-like mrna	391	1	2.90E-43	84.00%
2310	Cl_clus102443_Contig1	---NA---	448	0	-	-
2311	Cl_clus102444_Contig1	---NA---	316	0	-	-
2312	Cl_clus102445_Contig1	drosophila melanogaster heat shock protein cognate 3 (hsc70-3) transcript variant mrna	526	20	8.90E-134	79.30%
2313	Cl_clus102446_Contig1	---NA---	255	0	-	-
2314	Cl_clus102447_Contig1	---NA---	342	0	-	-
2315	Cl_clus102449_Contig1	---NA---	215	0	-	-

2316	Cl_clus102450_Contig1	---NA---	457	0 -	-	
2317	Cl_clus102452_Contig1	---NA---	425	0 -	-	
2318	Cl_clus102453_Contig1	---NA---	490	0 -	-	
2319	Cl_clus102454_Contig1	---NA---	395	0 -	-	
2320	Cl_clus102455_Contig1	---NA---	313	0 -	-	
2321	Cl_clus102451_Contig1	---NA---	256	0 -	-	
2322	Cl_clus102456_Contig1	---NA---	312	0 -	-	
2323	Cl_clus102458_Contig1	---NA---	215	0 -	-	
2324	Cl_clus102461_Contig1	---NA---	261	0 -	-	
2325	Cl_clus102462_Contig1	---NA---	616	0 -	-	
2326	Cl_clus102463_Contig1	---NA---	345	0 -	-	
2327	Cl_clus102464_Contig1	---NA---	260	0 -	-	
2328	Cl_clus102465_Contig1	---NA---	477	0 -	-	
2329	Cl_clus102466_Contig1	---NA---	376	0 -	-	
2330	Cl_clus102467_Contig1	---NA---	456	0 -	-	
2331	Cl_clus102468_Contig1	---NA---	312	0 -	-	
2332	Cl_clus102469_Contig1	---NA---	404	0 -	-	
2333	Cl_clus102470_Contig1	---NA---	257	0 -	-	
2334	Cl_clus102472_Contig1	---NA---	337	0 -	-	
2335	Cl_clus102473_Contig1	---NA---	424	0 -	-	
2336	Cl_clus102474_Contig1	---NA---	354	0 -	-	
2337	Cl_clus102475_Contig1	---NA---	358	0 -	-	
2338	Cl_clus102476_Contig1	---NA---	512	0 -	-	
2339	Cl_clus102477_Contig1	---NA---	453	0 -	-	
2340	Cl_clus102478_Contig1	---NA---	352	0 -	-	
2341	Cl_clus102479_Contig1	---NA---	355	0 -	-	
2342	Cl_clus102480_Contig1	---NA---	392	0 -	-	
2343	Cl_clus102481_Contig1	---NA---	262	0 -	-	
2344	Cl_clus102482_Contig1	---NA---	366	0 -	-	
2345	Cl_clus102484_Contig1	---NA---	427	0 -	-	
2346	Cl_clus102486_Contig1	---NA---	382	0 -	-	
2347	Cl_clus102488_Contig1	---NA---	320	0 -	-	
2348	Cl_clus102487_Contig1	---NA---	322	0 -	-	
2349	Cl_clus102489_Contig1	---NA---	586	0 -	-	
2350	Cl_clus102490_Contig1	---NA---	376	0 -	-	
2351	Cl_clus102491_Contig1	acartia pacifica clone acapa-cdna37-1 60s ribosomal protein l15 complete cds	363	10	4.60E-34	74.40%
2352	Cl_clus102492_Contig1	---NA---	384	0 -	-	
2353	Cl_clus102493_Contig1	---NA---	341	0 -	-	
2354	Cl_clus102495_Contig1	anopheles gambiae pest agap012048-pa complete cds	534	2	9.60E-140	77.50%
2355	Cl_clus102496_Contig1	---NA---	400	0 -	-	
2356	Cl_clus102494_Contig1	---NA---	393	0 -	-	
2357	Cl_clus102497_Contig1	---NA---	498	0 -	-	
2358	Cl_clus102498_Contig1	---NA---	561	0 -	-	
2359	Cl_clus102502_Contig1	---NA---	502	0 -	-	
2360	Cl_clus102501_Contig1	---NA---	343	0 -	-	
2361	Cl_clus102503_Contig1	---NA---	247	0 -	-	
2362	Cl_clus102504_Contig1	aedes aegypti mitochondrial processing peptidase beta subunit partial mrna	455	5	1.60E-66	73.40%
2363	Cl_clus102505_Contig1	---NA---	584	0 -	-	
2364	Cl_clus102506_Contig1	---NA---	386	0 -	-	
2365	Cl_clus102507_Contig1	---NA---	356	0 -	-	
2366	Cl_clus102508_Contig1	---NA---	476	0 -	-	
2367	Cl_clus102509_Contig1	---NA---	265	0 -	-	
2368	Cl_clus102510_Contig1	---NA---	356	0 -	-	
2369	Cl_clus102511_Contig1	maylandia zebra catalase-like mrna	340	6	2.40E-56	76.50%
2370	Cl_clus102513_Contig1	---NA---	393	0 -	-	
2371	Cl_clus102512_Contig1	---NA---	276	0 -	-	
2372	Cl_clus102515_Contig1	---NA---	387	0 -	-	
2373	Cl_clus102516_Contig1	---NA---	240	0 -	-	

2374	Cl_clus102517_Contig1	---NA---	352	0 -	-	
2375	Cl_clus102518_Contig1	---NA---	282	0 -	-	
2376	Cl_clus102519_Contig1	---NA---	254	0 -	-	
2377	Cl_clus102514_Contig1	---NA---	320	0 -	-	
2378	Cl_clus102499_Contig1	---NA---	242	0 -	-	
2379	Cl_clus102483_Contig1	---NA---	321	0 -	-	
2380	Cl_clus102471_Contig1	---NA---	290	0 -	-	
2381	Cl_clus102520_Contig1	---NA---	241	0 -	-	
2382	Cl_clus102521_Contig1	---NA---	230	0 -	-	
2383	Cl_clus102522_Contig1	aplysia californica nadh dehydrogenase	682	1	5.70E-87	74.00%
2384	Cl_clus102523_Contig1	---NA---	553	0 -	-	
2385	Cl_clus102525_Contig1	homarus americanus troponin c isoform 2a complete cds	713	1	2.70E-34	73.00%
2386	Cl_clus102526_Contig1	---NA---	381	0 -	-	
2387	Cl_clus102528_Contig1	---NA---	474	0 -	-	
2388	Cl_clus102529_Contig1	jakoba bahamiensis strain atcc 50695 complete genome	249	2	8.60E-35	75.00%
2389	Cl_clus102530_Contig1	---NA---	430	0 -	-	
2390	Cl_clus102531_Contig1	---NA---	286	0 -	-	
2391	Cl_clus102532_Contig1	---NA---	599	0 -	-	
2392	Cl_clus102533_Contig1	---NA---	283	0 -	-	
2393	Cl_clus102534_Contig1	---NA---	471	0 -	-	
2394	Cl_clus102535_Contig1	---NA---	483	0 -	-	
2395	Cl_clus102536_Contig1	---NA---	401	0 -	-	
2396	Cl_clus102537_Contig1	---NA---	232	0 -	-	
2397	Cl_clus102539_Contig1	---NA---	266	0 -	-	
2398	Cl_clus102540_Contig1	---NA---	443	0 -	-	
2399	Cl_clus102538_Contig1	---NA---	231	0 -	-	
2400	Cl_clus102541_Contig1	---NA---	298	0 -	-	
2401	Cl_clus102542_Contig1	---NA---	543	0 -	-	
2402	Cl_clus102543_Contig1	---NA---	250	0 -	-	
2403	Cl_clus102544_Contig1	---NA---	302	0 -	-	
2404	Cl_clus102545_Contig1	---NA---	536	0 -	-	
2405	Cl_clus102546_Contig1	---NA---	367	0 -	-	
2406	Cl_clus102547_Contig1	---NA---	208	0 -	-	
2407	Cl_clus102548_Contig1	---NA---	482	0 -	-	
2408	Cl_clus102549_Contig1	---NA---	296	0 -	-	
2409	Cl_clus102550_Contig1	---NA---	216	0 -	-	
2410	Cl_clus102551_Contig1	---NA---	206	0 -	-	
2411	Cl_clus102552_Contig1	---NA---	410	0 -	-	
2412	Cl_clus102553_Contig1	---NA---	460	0 -	-	
2413	Cl_clus102554_Contig1	---NA---	1501	0 -	-	
2414	Cl_clus102557_Contig1	---NA---	286	0 -	-	
2415	Cl_clus102558_Contig1	---NA---	386	0 -	-	
2416	Cl_clus102559_Contig1	---NA---	283	0 -	-	
2417	Cl_clus102560_Contig1	---NA---	367	0 -	-	
2418	Cl_clus102561_Contig1	---NA---	431	0 -	-	
2419	Cl_clus102563_Contig1	---NA---	234	0 -	-	
2420	Cl_clus102562_Contig1	---NA---	346	0 -	-	
2421	Cl_clus102564_Contig1	---NA---	232	0 -	-	
2422	Cl_clus102565_Contig1	---NA---	353	0 -	-	
2423	Cl_clus102566_Contig1	---NA---	423	0 -	-	
2424	Cl_clus102567_Contig1	---NA---	346	0 -	-	
2425	Cl_clus102568_Contig1	---NA---	325	0 -	-	
2426	Cl_clus102570_Contig1	---NA---	241	0 -	-	
2427	Cl_clus102571_Contig1	---NA---	329	0 -	-	
2428	Cl_clus102572_Contig1	---NA---	434	0 -	-	
2429	Cl_clus102573_Contig1	nomascus leucogenys s-phase kinase-associated protein 1 mrna	217	20	2.10E-54	82.00%
2430	Cl_clus102574_Contig1	---NA---	332	0 -	-	
2431	Cl_clus102575_Contig1	---NA---	341	0 -	-	
2432	Cl_clus102576_Contig1	---NA---	315	0 -	-	
2433	Cl_clus102577_Contig1	---NA---	257	0 -	-	

2434	Cl_clus102578_Contig1	---NA---	313	0	-	-
2435	Cl_clus102579_Contig1	---NA---	210	0	-	-
2436	Cl_clus102580_Contig1	---NA---	363	0	-	-
2437	Cl_clus102581_Contig1	plathemis lydia voucher atp synthase partial cds	334	14	6.60E-95	80.86%
2438	Cl_clus102582_Contig1	---NA---	392	0	-	-
2439	Cl_clus102583_Contig1	---NA---	309	0	-	-
2440	Cl_clus102584_Contig1	---NA---	615	0	-	-
2441	Cl_clus102585_Contig1	---NA---	397	0	-	-
2442	Cl_clus102586_Contig1	---NA---	276	0	-	-
2443	Cl_clus102587_Contig1	---NA---	275	0	-	-
2444	Cl_clus102588_Contig1	---NA---	224	0	-	-
2445	Cl_clus102589_Contig1	---NA---	437	0	-	-
2446	Cl_clus102591_Contig1	---NA---	319	0	-	-
2447	Cl_clus102592_Contig1	---NA---	210	0	-	-
2448	Cl_clus102593_Contig1	---NA---	291	0	-	-
2449	Cl_clus102594_Contig1	---NA---	271	0	-	-
2450	Cl_clus102590_Contig1	---NA---	460	0	-	-
2451	Cl_clus102595_Contig1	---NA---	288	0	-	-
2452	Cl_clus102596_Contig1	---NA---	269	0	-	-
2453	Cl_clus102597_Contig1	---NA---	499	0	-	-
2454	Cl_clus102598_Contig1	---NA---	502	0	-	-
2455	Cl_clus102599_Contig1	---NA---	315	0	-	-
2456	Cl_clus102600_Contig1	drosophila melanogaster desat1 transcript variant mrna	397	17	2.40E-44	74.24%
2457	Cl_clus102601_Contig1	---NA---	445	0	-	-
2458	Cl_clus102603_Contig1	---NA---	272	0	-	-
2459	Cl_clus102604_Contig1	---NA---	235	0	-	-
2460	Cl_clus102605_Contig1	---NA---	376	0	-	-
2461	Cl_clus102606_Contig1	---NA---	343	0	-	-
2462	Cl_clus102607_Contig1	---NA---	303	0	-	-
2463	Cl_clus102608_Contig1	---NA---	222	0	-	-
2464	Cl_clus102609_Contig1	---NA---	202	0	-	-
2465	Cl_clus102610_Contig1	---NA---	303	0	-	-
2466	Cl_clus102611_Contig1	---NA---	205	0	-	-
2467	Cl_clus102612_Contig1	---NA---	420	0	-	-
2468	Cl_clus102613_Contig1	---NA---	233	0	-	-
2469	Cl_clus102614_Contig1	---NA---	398	0	-	-
2470	Cl_clus102615_Contig1	---NA---	274	0	-	-
2471	Cl_clus102616_Contig1	---NA---	362	0	-	-
2472	Cl_clus102617_Contig1	---NA---	801	0	-	-
2473	Cl_clus102618_Contig1	---NA---	620	0	-	-
2474	Cl_clus102619_Contig1	---NA---	311	0	-	-
2475	Cl_clus102620_Contig1	---NA---	339	0	-	-
2476	Cl_clus102621_Contig1	---NA---	415	0	-	-
2477	Cl_clus102622_Contig1	---NA---	471	0	-	-
2478	Cl_clus102623_Contig1	---NA---	427	0	-	-
2479	Cl_clus102624_Contig1	---NA---	453	0	-	-
2480	Cl_clus102625_Contig1	---NA---	300	0	-	-
2481	Cl_clus102626_Contig1	---NA---	497	0	-	-
2482	Cl_clus102627_Contig1	---NA---	522	0	-	-
2483	Cl_clus102628_Contig1	---NA---	219	0	-	-
2484	Cl_clus102629_Contig1	---NA---	287	0	-	-
2485	Cl_clus102630_Contig1	---NA---	306	0	-	-
2486	Cl_clus102632_Contig1	---NA---	452	0	-	-
2487	Cl_clus102633_Contig1	---NA---	440	0	-	-
2488	Cl_clus102634_Contig1	---NA---	725	0	-	-
2489	Cl_clus102635_Contig1	arabidopsis thaliana at1g29930 f1n18_23 complete cds	300	20	2.20E-138	92.95%
2490	Cl_clus102637_Contig1	---NA---	500	0	-	-
2491	Cl_clus102639_Contig1	---NA---	276	0	-	-
2492	Cl_clus102640_Contig1	---NA---	217	0	-	-
2493	Cl_clus102641_Contig1	---NA---	254	0	-	-

2494	Cl_clus102642_Contig1	---NA---	202	0 -	-	
2495	Cl_clus102643_Contig1	---NA---	277	0 -	-	
2496	Cl_clus102644_Contig1	---NA---	343	0 -	-	
2497	Cl_clus102645_Contig1	---NA---	440	0 -	-	
2498	Cl_clus102646_Contig1	---NA---	329	0 -	-	
2499	Cl_clus102647_Contig1	---NA---	330	0 -	-	
2500	Cl_clus102649_Contig1	---NA---	340	0 -	-	
2501	Cl_clus102650_Contig1	---NA---	281	0 -	-	
2502	Cl_clus102652_Contig1	---NA---	321	0 -	-	
2503	Cl_clus102651_Contig1	---NA---	323	0 -	-	
2504	Cl_clus102654_Contig1	---NA---	422	0 -	-	
2505	Cl_clus102653_Contig1	---NA---	215	0 -	-	
2506	Cl_clus102655_Contig1	---NA---	353	0 -	-	
2507	Cl_clus102658_Contig1	---NA---	246	0 -	-	
2508	Cl_clus102659_Contig1	---NA---	248	0 -	-	
2509	Cl_clus102660_Contig1	---NA---	547	0 -	-	
2510	Cl_clus102662_Contig1	---NA---	244	0 -	-	
2511	Cl_clus102663_Contig1	---NA---	488	0 -	-	
2512	Cl_clus102661_Contig1	---NA---	224	0 -	-	
2513	Cl_clus102664_Contig1	---NA---	332	0 -	-	
2514	Cl_clus102665_Contig1	---NA---	240	0 -	-	
2515	Cl_clus102666_Contig1	---NA---	350	0 -	-	
2516	Cl_clus102668_Contig1	---NA---	364	0 -	-	
2517	Cl_clus102669_Contig1	---NA---	364	0 -	-	
2518	Cl_clus102671_Contig1	---NA---	509	0 -	-	
2519	Cl_clus102672_Contig1	---NA---	443	0 -	-	
2520	Cl_clus102673_Contig1	---NA---	242	0 -	-	
2521	Cl_clus102675_Contig1	---NA---	305	0 -	-	
2522	Cl_clus102676_Contig1	---NA---	407	0 -	-	
2523	Cl_clus102677_Contig1	---NA---	270	0 -	-	
2524	Cl_clus102678_Contig1	fenneropenaeus merguiensis tropomyosin complete cds	453	9	1.50E-117	80.67%
2525	Cl_clus102679_Contig1	megachile rotundata muscle lim protein mlp84b-like mrna	436	5	3.50E-68	79.00%
2526	Cl_clus102680_Contig1	---NA---	299	0 -	-	
2527	Cl_clus102681_Contig1	bacillus atticus partial genome	327	20	2.40E-81	77.05%
2528	Cl_clus102682_Contig1	phobaeticus serratipes mitochondrial complete genome	225	20	4.20E-38	86.25%
2529	Cl_clus102683_Contig1	---NA---	241	0 -	-	
2530	Cl_clus102685_Contig1	---NA---	362	0 -	-	
2531	Cl_clus102686_Contig1	aedes aegypti tropomyosin invertebrate partial mrna	331	20	5.70E-102	83.95%
2532	Cl_clus102687_Contig1	---NA---	644	0 -	-	
2533	Cl_clus102688_Contig1	---NA---	306	0 -	-	
2534	Cl_clus102689_Contig1	---NA---	485	0 -	-	
2535	Cl_clus102684_Contig1	---NA---	304	0 -	-	
2536	Cl_clus102690_Contig1	papilio xuthus mrna for muscle myosin heavy complete clone: fsg31	232	1	9.00E-47	79.00%
2537	Cl_clus102691_Contig2	drosophila melanogaster myosin heavy chain transcript variant mrna	283	20	4.20E-90	84.30%
2538	Cl_clus102692_Contig1	---NA---	203	0 -	-	
2539	Cl_clus102693_Contig1	clitarchus hookeri voucher clh36 trna-leu partial sequence and cytochrome oxidase subunit ii partial cds mitochondrial	774	20	0.00E+00	98.75%
2540	Cl_clus102694_Contig1	nasonia vitripennis tropomyosin 1 transcript variant mrna	600	20	5.70E-23	83.55%
2541	Cl_clus102695_Contig1	extatosoma tiaratum mitochondrial complete genome	512	20	9.20E-140	76.85%
